# Visible light C–H amidation of heteroarenes with benzoyl azides†
†Electronic supplementary information (ESI) available: Characterization and synthesis of new compounds, general procedures, proton and carbon NMR spectra, crystal structure analysis, gas chromatographic analyses. CCDC 1017661. For ESI and crystallographic data in CIF or other electronic format see DOI: 10.1039/c4sc02365j



**DOI:** 10.1039/c4sc02365j

**Published:** 2014-10-30

**Authors:** E. Brachet, T. Ghosh, I. Ghosh, B. König

**Affiliations:** a University of Regensburg Faculty of Chemistry and Pharmacy , Institute of Organic Chemistry , Universitätsstraße 31 , 93053 Regensburg , Germany . Email: Burkhard.Koenig@chemie.uni-regensburg.de

## Abstract

Benzoyl azides were used for the direct and atom economic C–H amidation of electron rich heteroarenes in the presence of phosphoric acid, a photocatalyst and visible light. Hetero-aromatic amides are obtained in good yields at very mild reaction conditions with dinitrogen as the only by-product. The reaction allows the use of aryl-, heteroaryl- or alkenyl acyl azides and has a wide scope for heteroarenes, including pyrroles, indole, furan, benzofuran and thiophene giving good regio-selectivities and yields.

## Abstract 2

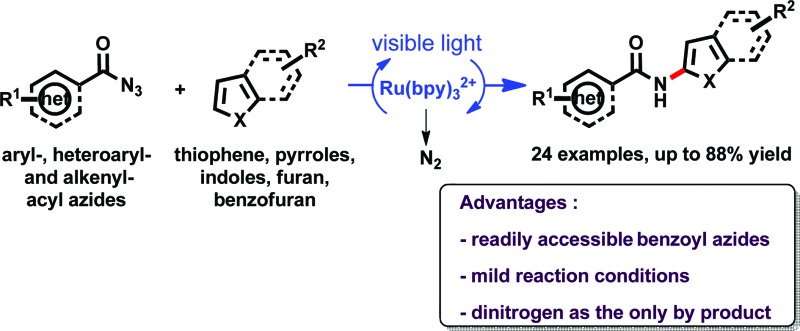
Benzoyl azides were used for the direct and atom economic C–H amidation of electron rich heteroarenes in the presence of phosphoric acid, a photocatalyst and visible light.

## Introduction

**Scheme 1:**
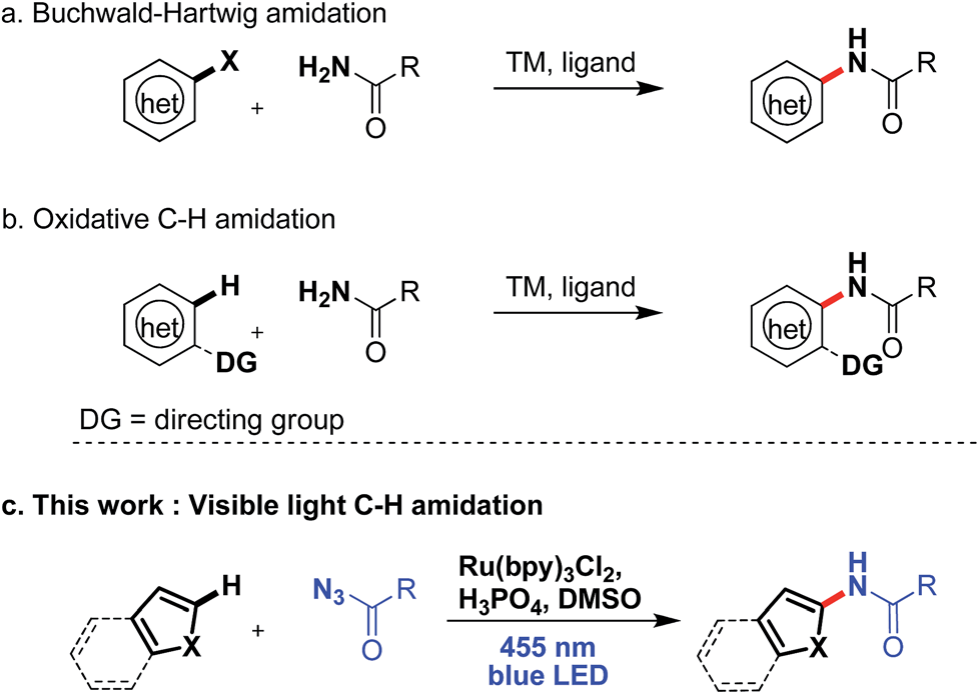
Catalytic aromatic amidation reactions.

Introducing amide moieties into organic molecules is a key transformation in organic chemistry due to the ubiquitous presence of this functional group in natural products, pharmaceuticals or functional materials.^1^ Amidation methods have been improved constantly in terms of functional group tolerance and conditions. Using transition metal catalysis, *e.g.* in Buchwald–Hartwig amidations, proved to be particularly advantageous.^2^ However, some limitations still exist: catalytic systems can be expensive, and often high reaction temperatures and pre-functionalized substrates, *e.g.* halides or pseudo-halides (Scheme 1a) are required. More recently, different groups have developed C–H amidations for heteroarenes or arenes with or without the use of a directing group (Scheme 1b).^3^ A drawback of this attractive strategy are the often required harsh reaction conditions. With the aim to improve direct C–H amidations towards milder conditions and a broader reaction scope we have developed a photocatalytic^4^ variant of the reaction (Scheme 1c).

Very few examples of photocatalyzed C–H amidations have been reported in the literature. MacMillan and coworkers^5^ described in 2013 the amidation of enamines yielding α-amino aldehydes with good yields and excellent stereocontrol. A second example was reported by Sanford *et al.* ^6^ exploring the reactivity of *N*-acyloxyphtalimides under visible light to generate phthalimide radicals that add to arenes and heteroarenes with good to excellent yields. This methods has been recently extended to *N*-chlorophthalimide as aminating agent under photocatalytic conditions.^7^ Yu *et al.* used activated hydroxylamines introducing amine groups into heteroarenes.^8^ The reported methods require a functionalized aminating reagent^5,8^ or yield phthalimide moieties.^6,7^

Inspired by this work we developed an alternative photocatalytic method for the functionalization of heteroarenes with aromatic amides, which may be particularly useful in synthesis. Many bioactive compounds show this structural motif and typical examples are found among analgestic and anti-parasitic compounds or hepatitis C drugs.^9^ A key advantage of our method is the introduction of the amide at a late stage of a synthesis of a more complex structure.

As aminating agents we choose benzoyl azides, which are easily accessible and allow for the introduction of simple benzamides into a substrate, which was so far not possible using visible light photocatalysis. Dinitrogen is the only stoichiometric by-product. Benzoyl azides have been used previously in transition metal catalysis, but the reaction requires the presence of a directing group.^10^ Exposed to UV irradiation^11^ benzoyl azides give oxazolines, dioxazoles or aziridines, but no conversion of heterocycles or formal amidation was observed under these conditions.^12^ We activate benzoyl azides using visible light and a photocatalyst allowing a direct C–H amidation of heteroarenes.

## Results and discussion

### Optimization of the reaction conditions

The reaction of benzoyl azide **1a** with *N*-methylpyrrole **2a** was used to establish and optimize the conditions. The results of the optimization are summarized in Table 1. Using ruthenium(ii)trisbipyridine dichloride (**A**) as the photosensitizer in DMSO with blue light irradiation did not yield any amidation product. Part of the benzoyl azide is converted into the corresponding isocyanate by Curtius rearrangement and benzamide, as the nitrene hydrogen abstraction product (see ESI† for GC-MS analyses). The addition of an acid as additive is essential for the formation of the desired amidation product. Isocyanates are not stable under these conditions and only their decomposition products are observed. Initial low amidation product yields of 10% were improved to 40% after 12 h of irradiation using phosphoric acid, while changing the photocatalyst or the solvent had no significant effect on the product yield. Working at lower concentrations gave 65% product yield already after 4 h of blue light irradiation. Control experiments showed that the photocatalyst, irradiation, and acid are required to get full conversion of the starting material (entries 1, 15 and 16). The amount of ruthenium catalyst can be reduced to 1 mol% to provide an acceptable product yield of 51%.

**Table 1:** Optimization of the reaction conditions

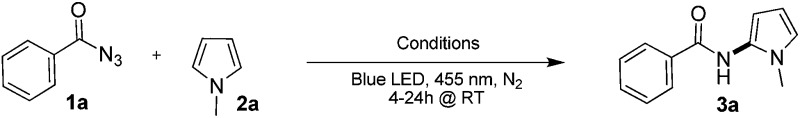					
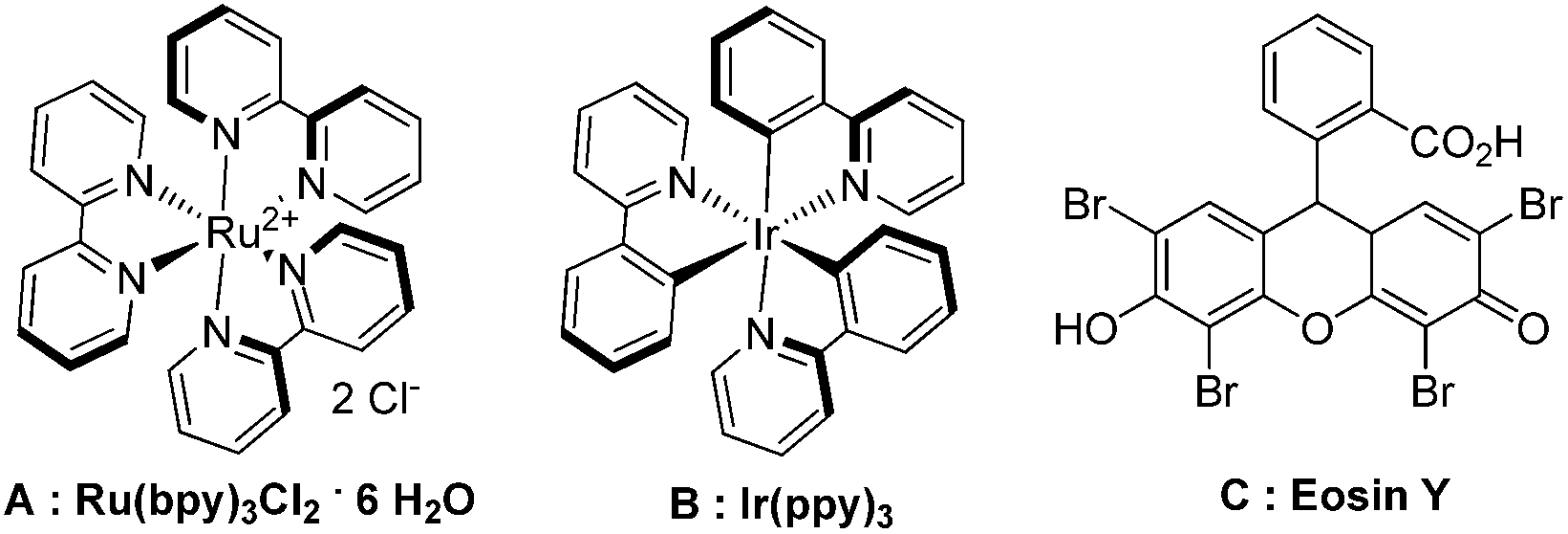					
Entry	Cat^*a*^	Additive^*b*^	Solvent	Conv. [%]	Yield^*c*^
1	A	—	DMSO (0.34 M)	∼35%	—^*d*^
2	A	(BnO)_2_P(O)OH	DMSO (0.34 M)	100	10^*a*^%
3	A	(BnO)_2_P(O)OH	DMSO (0.34 M)	100	30%
4	A	Benzoic acid	DMSO (0.34 M)	100	8%
5	A	Acetic acid	DMSO (0.34 M)	100	10%
6	A	PTSA	DMSO (0.34 M)	100	0%
7	A	H_3_PO_4_	DMSO (0.34 M)	100	40%
8	B	H_3_PO_4_	DMSO (0.34 M)	60	—
9	C	H_3_PO_4_	DMSO (0.34 M)	75	—
10	A	H_3_PO_4_	DMF (0.34 M)	60	—
11	A	H_3_PO_4_	ACN (0.34 M)	20	—
12	A	H_3_PO_4_	DMSO (0.09 M)	100	65^*e*^%
13	A	H_3_PO_4_	DMSO (0.09 M)	100	51^*f*^%
14	A	H_3_PO_4_	DMSO (0.09 M)	95	30^*g*^%
15	—	H_3_PO_4_	DMSO (0.09 M)	0	—
16	A	H_3_PO_4_	DMSO (0.09 M)	0	—^*h*^

^*a*^2.5 mol% of catalyst.

^*b*^2 equiv. of additive with respect to **1a**.

^*c*^Isolated yields.

^*d*^Phenylisocyanate and benzamide are obtained.

^*e*^Reaction conditions: **1a** (0.34 mmol, 1 equiv.), **2a** (1.7 mmol, 5 equiv.), additives (0.68 mmol, 2 equiv.) and photocatalyst (8.5 μmol, 2.5 mol%) in dry solvent (0.09 M) under N_2_ was irradiated with blue light for 4 h.

^*f*^1 mol% of the catalyst used.

^*g*^1 equiv. of *N*-methylpyrrole.

^*h*^Without light.

### Scope of the reaction

Having established the reaction conditions we explored the scope of benzoyl azides (Table 2). Benzoyl azides (**1a–i**) are readily obtained from their corresponding benzoyl chlorides and sodium azide. Gratifyingly, differently substituted benzoyl azides react with *N*-methylpyrrole **2a** in good yields and a perfect selectivity for position 2 as confirmed by the crystal structure analysis of compound **3b** (Table 2, entry 2). Benzoyl azides bearing electron donating groups or electron withdrawing groups are tolerated. The sterically hindered 1-naphthoyl azide **1c** reacts, albeit with a lower yield of 47%. Sensitive groups like cyano or ester moieties are well tolerated (compound **3e** and **3f** respectively). It was reported that benzoyl azides react under light irradiation with cyanide groups to give oxadiazole,^11,12^ but in our conditions we only obtained the desired product in 63% yield. Product **3g** bearing a chlorine atom allows for subsequent functionalization by transition metal mediated cross-coupling,^13^ and the use of heteroaryl acyl azide **1h** yields diheteroaryl amide **3h** in 49%. However, alkyl, phenyl, diphenylphosphoryl or benzyl acyl azides do not react under the photocatalytic conditions (entry 9). We explain this selectivity and limitation in substrate scope by the available energy of the long-lived [Ru(bpy)_3_^2+^]* triplet state (46 kcal mol^–1^), which is sufficient for energy transfer to acyl azides (first electronically excited triplet state ∼41 kcal mol^–1^), but not for *e.g.* phenyl azide (68 kcal mol^–1^) (*vide infra* for mechanistic proposal and ESI† for data and references).

**Table 2:** Scope of benzoyl azides for the photocatalyzed C–H amidation^*a*^

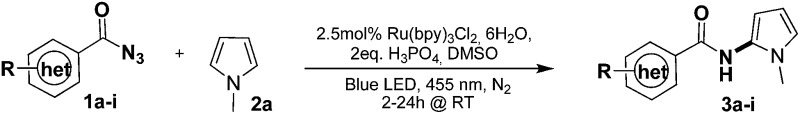			
Entry	Benzoyl azide	Product	Yield^*b*^ [%]
1	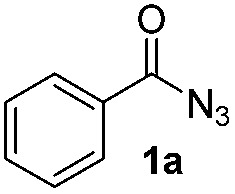	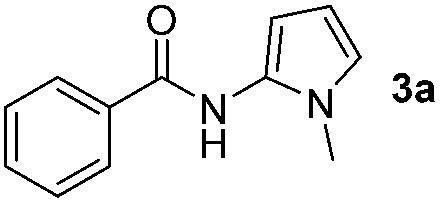	65%
2	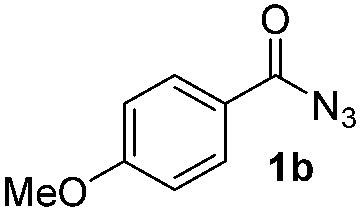	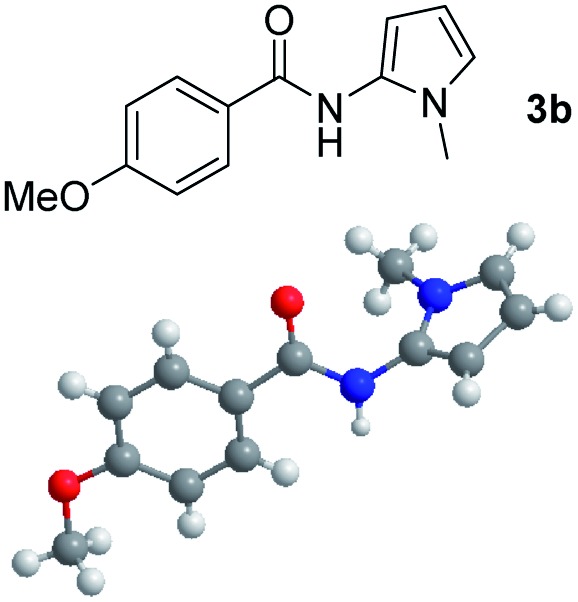	71%
3	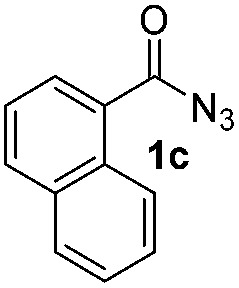	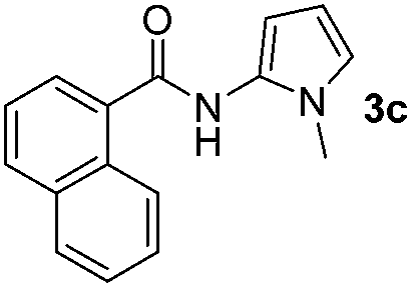	47%
4	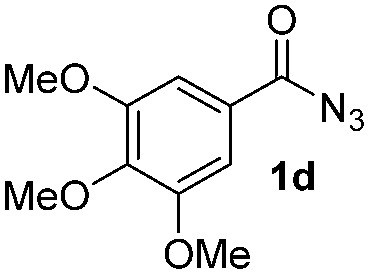	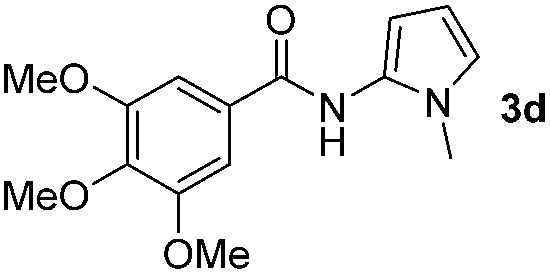	54%
5	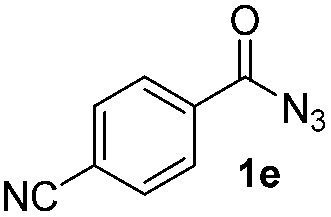	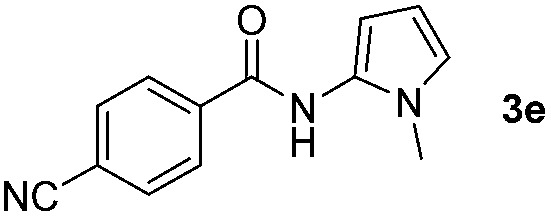	63%
6	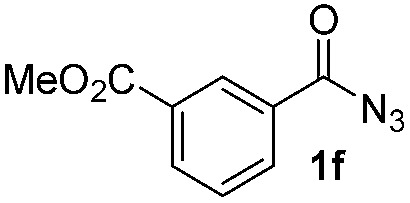	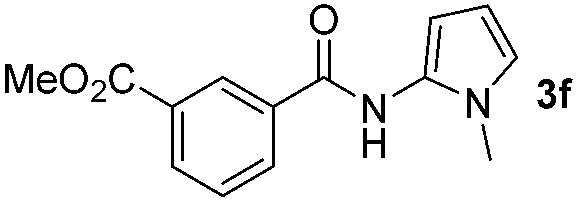	46%
7	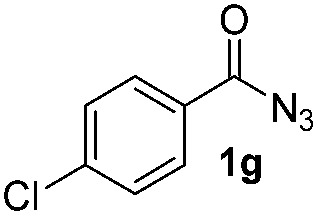	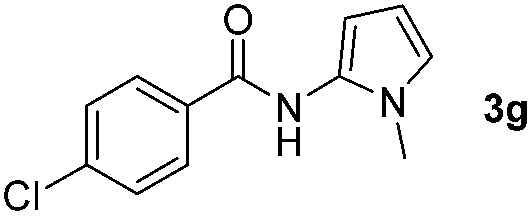	61%
8	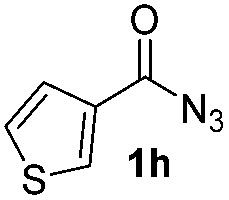	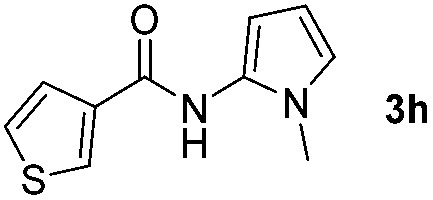	49%
9	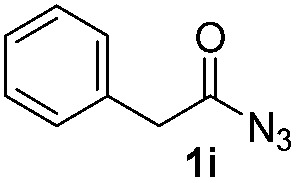	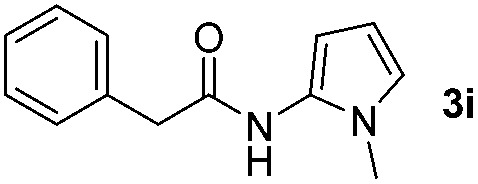	0%

^*a*^Reaction conditions: **1a–i** (0.34 mmol, 1 equiv.), **2a–i** (1.7 mmol, 5 equiv.), H_3_PO_4_ (0.68 mmol, 2 equiv.) and Ru(bpy)_3_Cl_2_·6H_2_O (8.5 μmol, 2.5 mol%) in dry DMSO (0.09 M) were irradiated with blue light under N_2_.

^*b*^Isolated yield, average of two reactions.

We then explored the scope of the reaction towards substituted pyrroles and other heterocycles like indole, furan, thiophene, and benzofuran (Table 3). Substituted pyrroles; either with electron withdrawing or electron donating groups gave the corresponding products in moderate to good yields of up to 88%.

**Table 3:** Scope of heteroarenes undergoing photocatalytic C–H amidation^*a*^

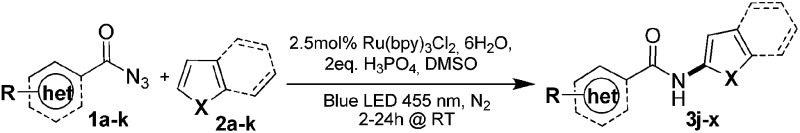				
Entry	Benzoyl azide	Heteroarene	Product	Yield^*b*^ [%]
1	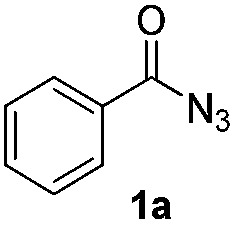	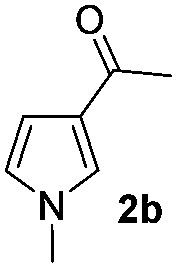	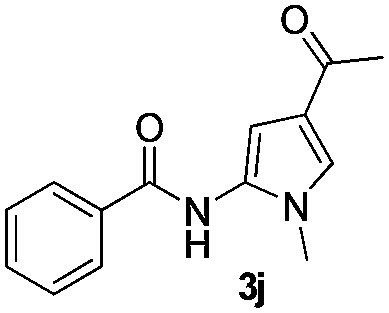	65%
2	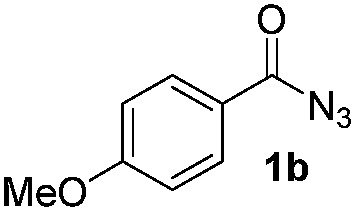	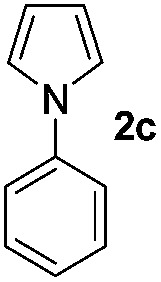	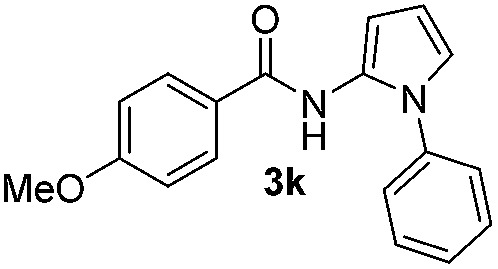	72%
3		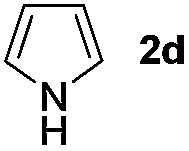	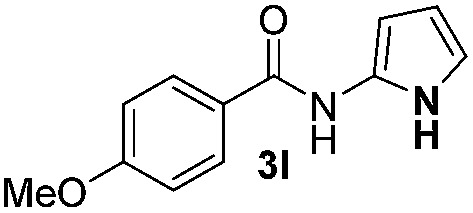	69%
4		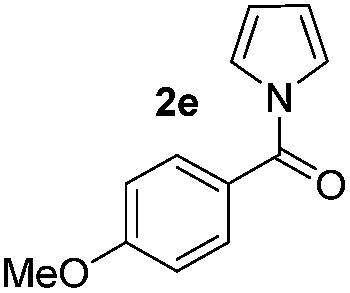	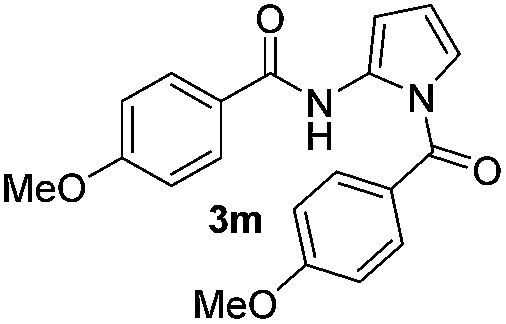	64%
5		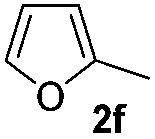	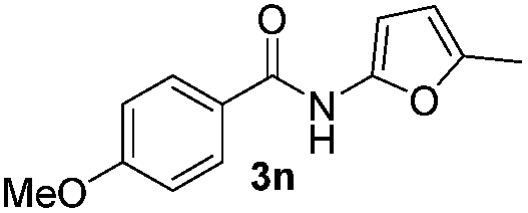	49%
6		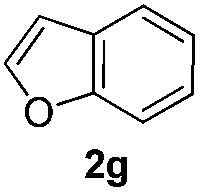	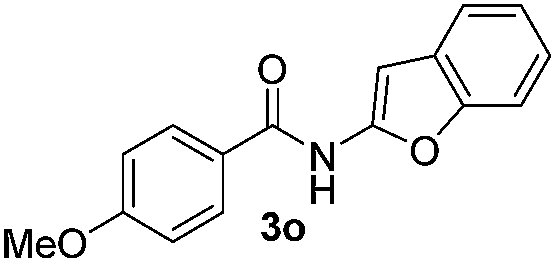	15%
7		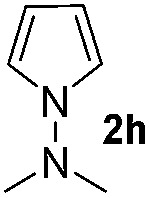	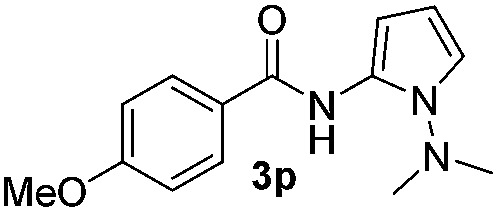	59%
8	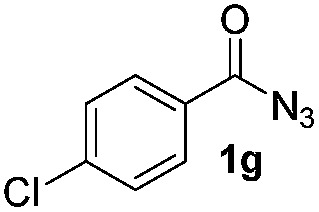	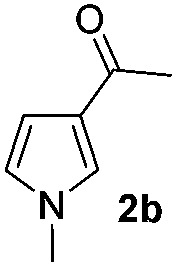	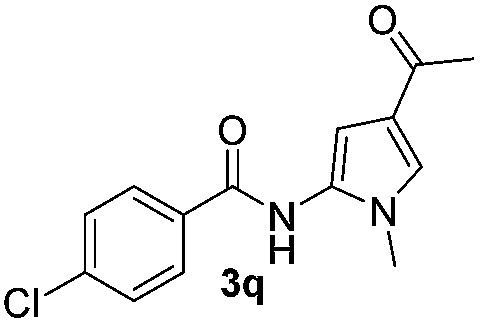	46%
9		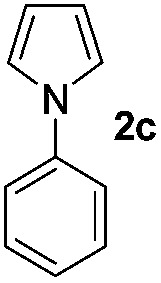	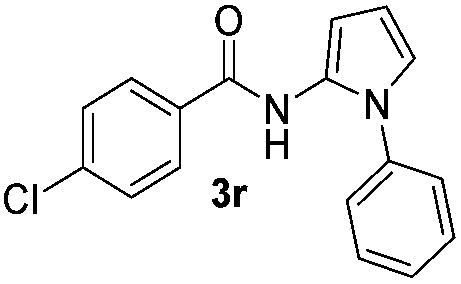	88%
10		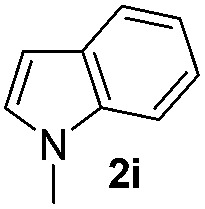	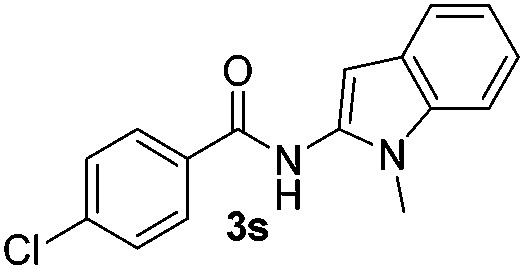	59%
11		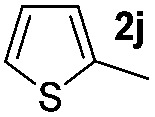	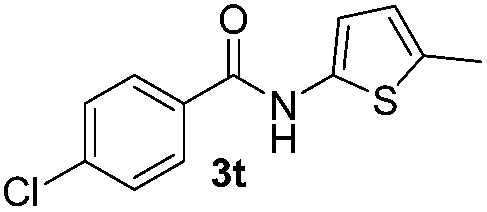	35%
12	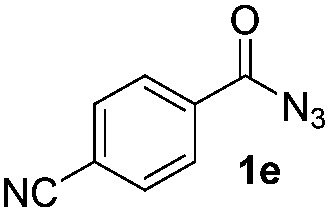	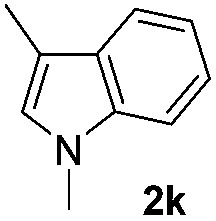	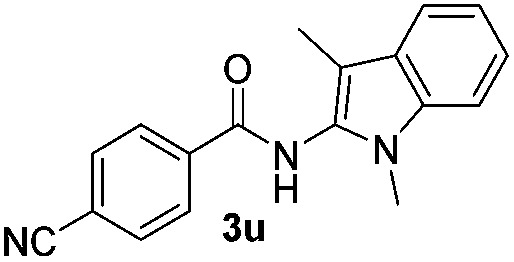	61%
13		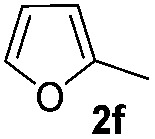	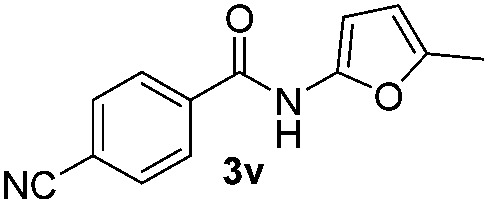	44%
14	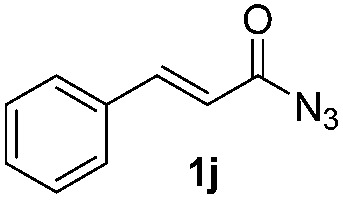	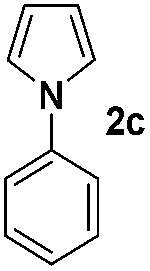	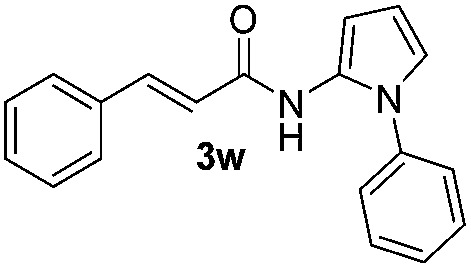	55%
15	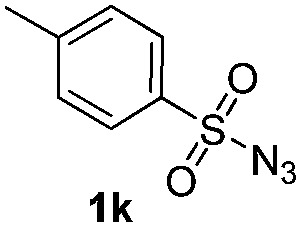	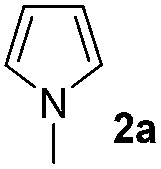	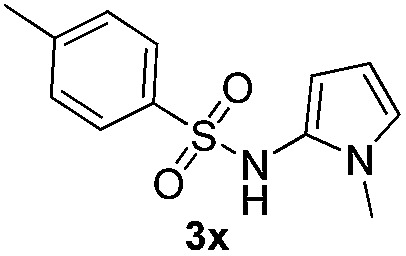	52%

^*a*^Reaction conditions: **1a–k** (0.34 mmol, 1 equiv.), **2a–k** (1.7 mmol, 5 equiv.), H_3_PO_4_ (0.68 mmol, 2 equiv.) and Ru(bpy)_3_Cl_2_·6H_2_O (8.5 μmol, 2.5 mol%) in dry DMSO (0.09 M) were irradiated with blue light under N_2_.

^*b*^Isolated yield, average of two reactions.

Protection of the N–H moiety of pyrrole is not necessary, which is advantageous for the functionalization of such heterocycles and their late stage amidation.^14^ (Table 3, entry 3). *N*-Aminoazoles are found in bioactive compounds,^15^ but their sensitive hydrazine group limits a direct functionalization in synthesis.^16^ Visible light photocatalysis allows the conversion with benzoyl azide **1b** yielding the substituted *N*-aminopyrrole **3p** in 59%. Indoles, furan and thiophene gave the expected amidation products in 35–61% yield. Analogous to benzoyl azides, we investigated the reaction with alkenyl acyl azide and sulfonylazide, which are cleanly converted to the corresponding products in more than 50% yield (Table 3, entries 14 and 15).

**Scheme 2:**
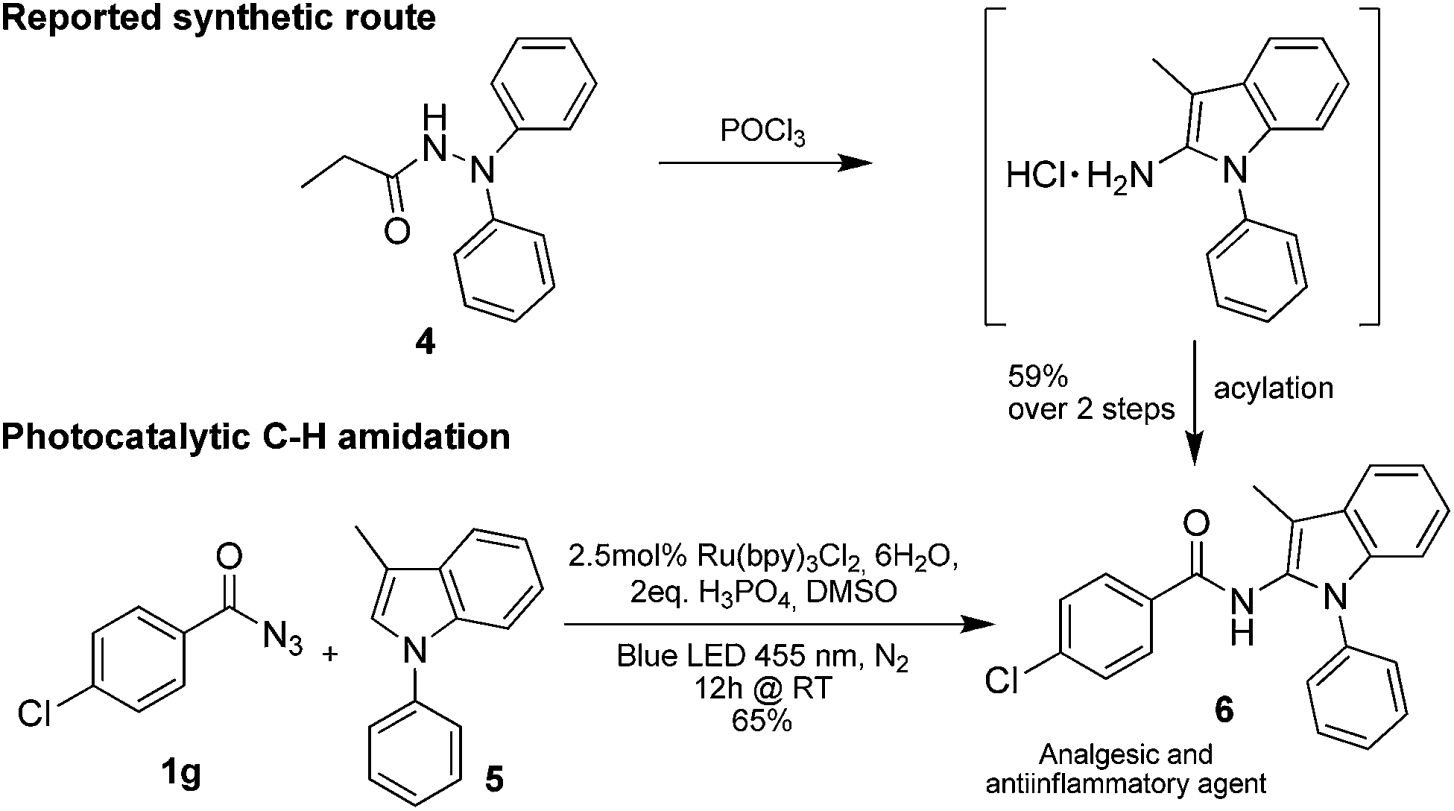
Synthesis of compound **6** using photocatalytic C–H amidation with benzoyl azide **1g**.

Only benzofuran reacts with a significantly lower yield of 15% (Table 3, entry 6), due to the formation of a side product (see mechanistic proposal). To illustrate the potential of the new method for synthesis, it was applied to the preparation of amide **6** having analgesic and anti-inflammatory properties (Scheme 2).^7^ The previously reported route used trifold substituted hydrazine **4**, which is cyclized to the indole core and acylated to give the desired product **6**. This route is limited to symmetrical diarylhydrazines to avoid product mixtures in the cyclization step. The photocatalytic C–H-amidation gave the target compound in one step with a slightly improved yield of 65% (Scheme 2).

**Scheme 3:**
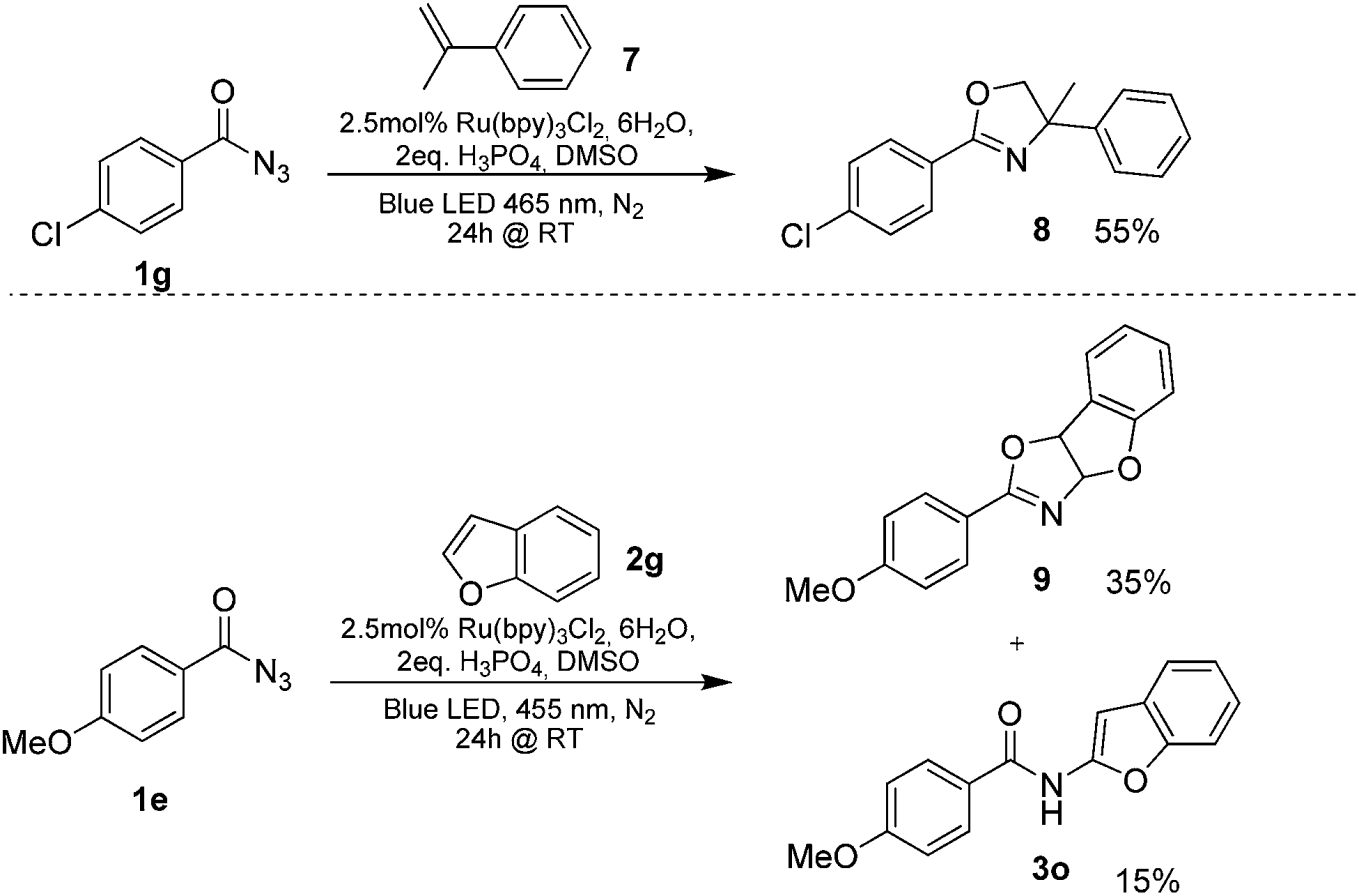
Isolated oxazolines from the photoreaction.

### Mechanistic investigations

**Scheme 4:**
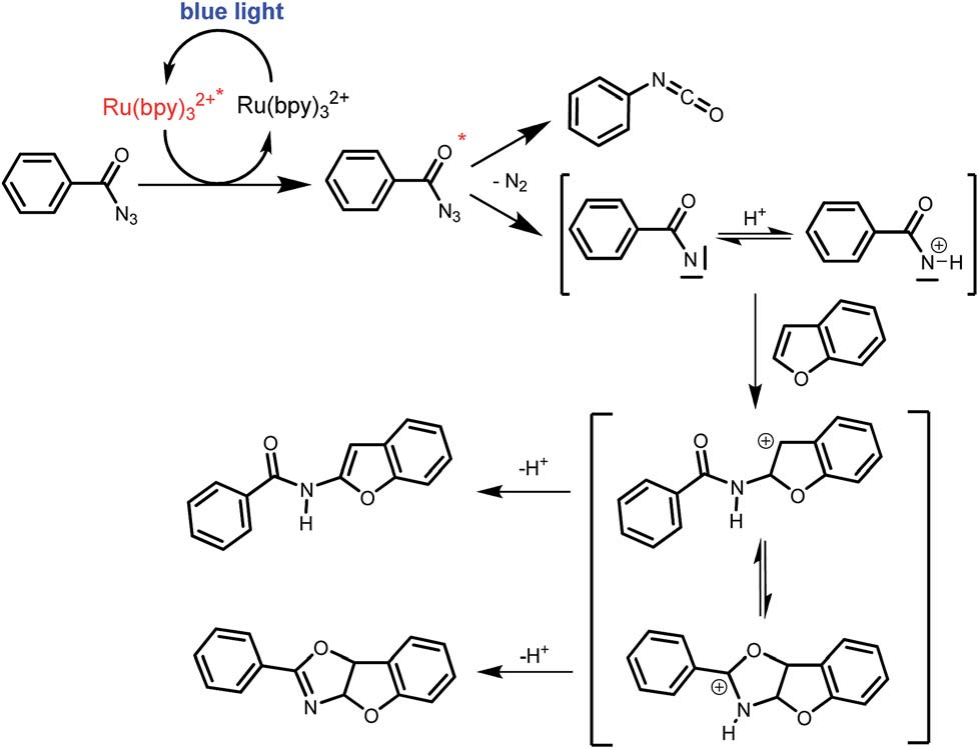
Proposed mechanism of the photo CH-amidation of benzofuran with benzoyl azide in the presence of acid, Ru(bpy)_3_Cl_2_ and blue light.

Benzoyl azides are known to loose dinitrogen upon sensitization yielding nitrenes, which convert into isocyanates by the Curtius rearrangement.^17^ In the presence of hydrogen donors benzamides are obtained and the reaction with double bonds yields aziridines or oxazolines.^11,12,18^ The ratio between isocyanate and nitrene formation from the excited benzoyl azide is reported to be independent of the presence and the amount of alkenes as trapping reagent;^19^ the photophysical mechanism of sensitized and direct benzoyl azide decomposition has been investigated in detail.^20^ Some recent examples using azides in photocatalyzed reactions suggest a nitrene intermediate from single electron transfer or by sensitization.^21^ Based on these findings and a series of control experiments, we propose the following mechanism for the described amidation reaction: Ru(bpy)_3_Cl_2_ acts upon blue light irradiation as a triplet sensitizer for benzoyl azides. The interaction of the excited ruthenium complex and the azide was confirmed by a Stern–Volmer experiment (see ESI† for data). An electron transfer pathway is excluded, as benzoyl azides are insufficiently electron deficient to be easily reduced by ruthenium photocatalysis: the reduction potentials of [Ru(bpy)_3_Cl_2_]* and benzoyl azide **1b**, respectively, are –0.89 and –1.49 V *versus* the saturated calomel electrode (SCE). Addition of TEMPO does not interfere with the reaction, which also indicates the absence of radical intermediates in this reaction. The energy transfer triggers the loss of dinitrogen yielding the benzoyl nitrene, which converts in part *via* the Curtius rearrangement to the corresponding isocyanate. GC-MS analysis of the reaction mixture in the absence of acid clearly identified the isocyanate. However, amidation products are only observed in the presence of an acid, *e.g.* H_3_PO_4_, in the reaction mixture. As neither the absorption of the ruthenium complex and the benzoyl azide, nor the emission of the ruthenium complex and the reduction potential of the benzoyl azide (from cyclic voltammetry) change by the addition of acid (see ESI† for data), the sensitization step is not affected.^22^ The benzoyl nitrene may be protonated under the strongly acidic conditions, as analogously reported for substituted aryl nitrenes,^23^ giving electrophilic nitrenium ions, which react with the electron rich heteroarene. The carbenium ion intermediate reacts under a loss of a proton and rearomatization to the amidation product.^24^ In the case of benzofurane and α-methylstyrene **7** the oxazoline formation^25^ is an alternative reaction pathway. Benzoyl azide **1g** reacts with α-methylstyrene **7** under blue light irradiation yielding oxazoline **8** in 55% yield (Scheme 3), while in the reaction with benzofurane, oxazoline **9** was obtained as the major product accompanied by benzofuran amide **3o** in 15%. Isolated oxazoline **9** does not convert into benzofuran **3o** when exposed to the reaction conditions, which indicates that its formation is irreversible and it is not a reaction intermediate (Scheme 4).

## Conclusions

We have reported the C–H amidation of heterocycles with benzoyl azides under very mild and practical conditions using visible light. The only byproduct of this atom economic process is dinitrogen. Heterocycles, such as pyrroles, indoles, furan, benzofuran or thiophene and substituted benzoyl azides give the corresponding amide coupling products in moderate to good yields in a single step. This first application of benzoyl azides in photocatalysis provides C–H amidation products of classic benzamides, which are typical structural motifs of many bioactive compounds. The photoreaction may therefore be a valuable alternative in the synthesis of bioactive heterocycles to the previously reported use of phthalimides or functionalized amines. A particular advantage of the new method is the amidation under mild conditions allowing for a late stage functionalization of complex heterocyclic molecules.

## Experimental section

### Standard procedure for the photocatalyzed CH amidation reaction

In a 5 mL snap vial equipped with magnetic stirring bar Ru(bpy)3Cl_2_·6H_2_O, (0.025 equiv.), benzoyl azide (1 equiv.), *o*-H_3_PO_4_ (2 equiv.) and the heteroarene (5 equiv.) were dissolved in dry DMSO (0.09 mmol mL^–1^) and the resulting mixture was degassed by “pump–freeze–thaw” cycles (×2) *via* a syringe needle and filled with nitrogen. The vial was irradiated through the vial's plane bottom side using blue LEDs. After complete conversion of the starting material, as monitored by TLC, the pressure in the vial was released by a needle and the reaction mixture was transferred into a separating funnel, diluted with ethyl acetate and washed with 15 mL of water. The aqueous layer was washed three times with ethyl acetate. The combined organic layers were dried over MgSO_4_, filtered, and concentrated in vacuum. Purification of the crude product was achieved by flash column chromatography using petrol ether/ethyl acetate as eluent.
