# Supplementary material for: Visible light C–H amidation of heteroarenes with benzoyl azides
Source: Chem Sci. 2014 Oct 30;6(2):987–92. doi: 10.1039/c4sc02365j (PMC5811135; doi:10.1039/c4sc02365j)
Supplement: Supplementary file 1 [file SC-006-C4SC02365J-s001.pdf]

Electronic Supplementary Material (ESI) for Chemical Science.
This journal is © The Royal Society of Chemistry 2014

Supporting Information

Visible Light C-H Amidation of Heteroarenes with Benzoyl Azides

Etienne Brachet, Tamal Ghosh, Indrajit Ghosh and Burkhard König*

Institut für Organische Chemie, Universität Regensburg, Universitätsstraße 31, D-93053 Regensburg, Germany

page

1) General Information 2
2) General Procedures 2
a) Preparation and characterization of acyl azides 2
b) General procedure for the reaction of acyl azide with heteroarenes 5
3) Proton and carbon NMR spectra of prepared compounds 13
4) Crystallographic data 51
5) Cyclic voltammetry of compound 1b 53
6) Stern-Volmer analysis for $Ru(bpy)_3Cl_2$ x 6 H_2O + compound 1b 55
7) Time profile of the reaction of 1b with 2a with and without blue light irradiation. 57
8) GC-MS analysis of crude reaction mixtures 58
9) References 62

1) General Information

^{1}H NMR spectra were recorded on a Bruker Avance 300 MHz spectrometer in $CDCl_3$ or acetone-d_6 solution with internal solvent signal as reference. ^{13}C NMR were recorded on a 75 MHz spectrometer in $CDCl_3$ or acetone-d_6 solution and referenced to the internal solvent signal. ^{1}H NMR data are reported as follows: chemical shift (ppm), multiplicity (s = singlet, d = doublet, t = triplet, q = quartet, quint = quintet, dd = doublet of doublets, ddd = doublet of doublet of doublets, td = triplet of doublets, qd = quartet of doublets, m = multiplet, br. s. = broad singlet), and coupling constants (Hz) and number of protons. All reactions were monitored by thin-layer chromatography using Merck silica gel plates 60 F_{254}; visualization was accomplished with short wavelength UV light (254 nm) and/or staining with appropriate stains (anisaldehyde, orthophosphomolybdic acid). Standard flash chromatography was performed using silica gel of particle size 40−63 µm. $Ru(bipy)_3Cl_2.6H_2O$ was purchased from Sigma Aldrich. All other commercially available reagents and solvents were used without further purification. The blue light irradiation was performed using high-power LEDs (Philips LUXEON® Rebel (1W, λ_{Ex} = 455 ± 15 nm, 3.5 V, 145 lm, 700mA)).

2) General Procedures

a) Preparation and characterization of acyl azides

General procedure for the preparation of acylazides.

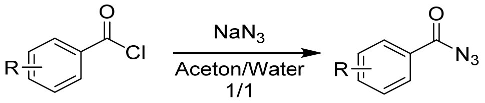

The appropriate benzoyl chloride (5 mmol, 1 equiv) was dissolved in 7 mL of acetone. After cooling the reaction mixture to 0 °C (using an ice bath) sodium azide (5.5 mmol, 1.1 equiv) in 7 mL of water was added dropwise at 0 °C. The resulting mixture was stirred for 2 hours. The resulting mixture was then transferred to a separating funnel and the organic layer was collected, dried over magnesium sulfate and evaporated (note that the temperature of the heating bath should not exceeded 30 °C) to give the corresponding acyl azide. **Caution: It is known that these reagents are potential explosives and must be therefore handled with care and stored in a fridge. It is also recommended to prepare acyl azides at a maximum of 10 mmol scale to avoid risks.**

Compound 1a: Benzoyl azide[1]

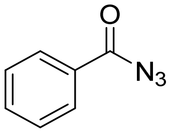

^{1}H NMR (300 MHz, acetone-d_6) δ 8.07 – 7.97 (m, 2H), 7.78 – 7.66 (m, 1H), 7.61 – 7.50 (m, 2H); ^{13}C NMR (75 MHz, acetone-d_6) δ 172.89, 135.39, 131.54, 130.03, 129.77.

Compound 1b: 4-Methoxybenzoyl azide[2]

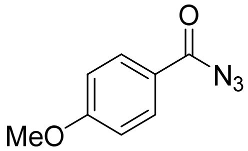

^{1}H NMR (300 MHz, acetone-d$_6$) δ 7.96 (d, J = 9.0 Hz, 2H), 7.05 (d, J = 9.0 Hz, 2H), 3.90 (s, 3H); ^{13}C NMR (75 MHz, acetone-d$_6$) δ 171.90, 165.77, 132.35, 123.83, 115.00, 56.10.

Compound 1c: 1-Naphthoyl azide[3]

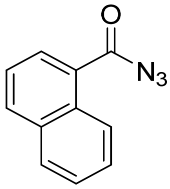

^{1}H NMR (300 MHz, acetone-d$_6$) δ 8.06 (dd, J = 5.1, 4.4 Hz, 1H), 7.90 (dd, J = 7.0, 2.3 Hz, 1H), 7.75 (d, J = 8.0 Hz, 1H), 7.62 – 7.49 (m, 2H), 7.45 – 7.34 (m, 2H); ^{13}C NMR (75 MHz, acetone-d$_6$) δ 161.34, 136.10, 135.06, 132.22, 129.03, 127.66, 127.57, 126.88, 126.56, 123.17, 123.02.

Compound 1d: 3,4,5-Trimethoxybenzoyl azide[4]

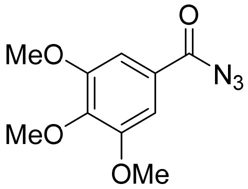

Colorless solid; m.p. = 85-87°C; ^{1}H NMR (300 MHz, acetone-d$_6$) δ 7.27 (s, 2H), 3.89 (s, 6H), 3.82 (s, 3H); ^{13}C NMR (75 MHz, acetone-d$_6$) δ 172.13, 154.29, 144.75, 126.30, 107.41, 60.76, 56.57.

Compound 1e: 4-Cyanobenzoyl azide[5]

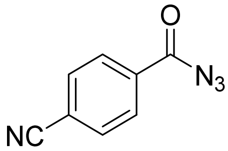

Colorless solid; m.p. = 82-84°C; ^{1}H NMR (300 MHz, acetone-d$_6$) δ 8.17 (d, J = 8.7 Hz, 2H), 7.98 (d, J = 8.6 Hz, 2H); ^{13}C NMR (75 MHz, acetone-d$_6$) δ 171.92, 135.12, 133.66, 130.60, 118.41, 118.20.

Compound 1f: Methyl 3-(azidocarbonyl)benzoate

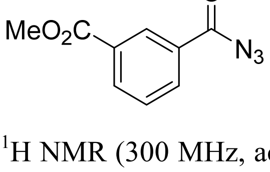

^{1}H NMR (300 MHz, acetone-d$_6$) δ 8.56 (t, J = 1.5 Hz, 1H), 8.33 – 8.26 (m, 1H), 8.26 – 8.18 (m, 1H), 7.71 (t, J = 7.8 Hz, 1H), 3.94 (s, 3H); ^{13}C NMR (75 MHz, acetone-d$_6$) δ 172.23, 166.16, 135.67, 134.13, 132.02, 131.90, 130.63, 130.36, 52.79.

Compound 1g: 4-Chlorobenzoyl azide[6]

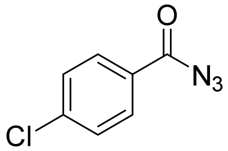

^{1}H NMR (300 MHz, acetone-d_6) δ 8.00 (d, J = 8.8 Hz, 2H), 7.59 (d, J = 8.8 Hz, 2H); ^{13}C NMR (75 MHz, acetone-d_6) δ 172.00, 141.18, 131.71, 130.26, 130.01.

Compound 1h: Thiophene-3-carbonyl azide[1]

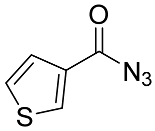

^{1}H NMR (300 MHz, acetone-d_6) δ 8.61 – 7.99 (m, 1H), 7.77 – 7.20 (m, 2H); ^{13}C NMR (75 MHz, acetone-d_6) δ 167.81, 135.67, 135.23, 128.60, 127.97.

Compound 1i: 2-Phenylacetyl azide[7]

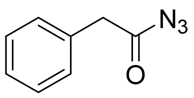

^{1}H NMR (300 MHz, acetone-d_6) δ 7.35 – 7.24 (m, 1H), 4.38 (d, J = 6.2 Hz, 1H); ^{13}C NMR (75 MHz, acetone-d_6) δ 156.98, 139.59, 129.28, 128.30, 128.05, 45.14.

Compound 1j: Cinnamoyl azide[1]

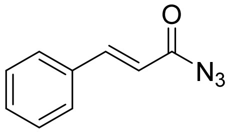

^{1}H NMR (300 MHz, acetone-d_6) δ 7.85 – 7.68 (m, 1H), 7.56 – 7.34 (m, 1H), 6.59 (d, J = 15.9 Hz, 1H); ^{13}C NMR (75 MHz, acetone-d_6) δ 172.34, 147.36, 134.83, 132.01, 129.93, 129.61, 119.89.

Compound 1k: 4-Methylbenzenesulfonyl azide[8]

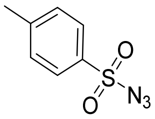

^{1}H NMR (300 MHz, acetone-d_6) δ 7.91 (d, J = 8.4 Hz, 2H), 7.57 (d, J = 8.6 Hz, 2H), 2.50 (s, 2H); ^{13}C NMR (75 MHz, acetone-d_6) δ 147.52, 136.40, 131.39, 128.33, 21.62.

b) General procedure for the reaction of acyl azide with heteroarenes

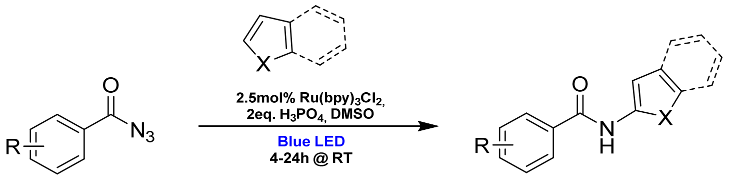

In a 5 mL snap vial equipped with magnetic stirring bar Ru(bipy)$_3$Cl$_2$.6H$_2$O, (0.025 equiv), benzoyl azide (1 equiv), H_3PO_4 (2 equiv) and the heteroarene (5 equiv) were dissolved in dry DMSO (0.09 mmol/mL) and the resulting mixture was degassed by "pump-freeze-thaw" cycles (×2) via a syringe needle and filled with nitrogen. The vial was irradiated through the vial's plane bottom side using blue LEDs. After complete conversion of the starting material (monitored by TLC) the pressure inside the vial was released by a syringe needle and the reaction mixture was transferred into a separating funnel, diluted with ethyl acetate and washed with 15 mL of water. The aqueous layer was washed three times with ethyl acetate. The combined organic layers were dried over $MgSO_4$, filtered and concentrated in vacuum. Purification of the crude product was achieved by flash column chromatography using petrol ether/ethyl acetate as eluent. NMR spectra of the compounds were typically recorded in acetone-d$_6$, because of their limited stability in $CDCl_3$.

Compound 3a: *N*-(1-Methyl-1H-pyrrol-2-yl)benzamide

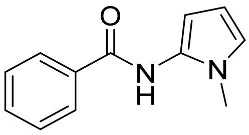

65%, R_f = 0.30 (PE:EA, 7:3); viscous oil; ^{1}H NMR (300 MHz, $CDCl_3$) δ 7.88 (d, *J* = 7.3 Hz, 2H), 7.61 – 7.53 (m, 2H), 7.48 (t, *J* = 7.4 Hz, 1H), 6.58 (m, 1H), 6.12 (t, *J* = 3.3 Hz, 1H), 6.05 (m, 1H), 3.52 (s, 3H); ^{13}C NMR (75 MHz, $CDCl_3$) δ 167.55, 133.75, 132.24, 128.89, 127.43, 124.84, 120.19, 106.78, 104.11, 33.12; HR-MS (ESI): m/z calculated for $C_{12}H_{13}N_2O$ 201,1028 obtained 201,1024.

Compound 3b: 4-Methoxy-*N*-(1-methyl-1H-pyrrol-2-yl)benzamide

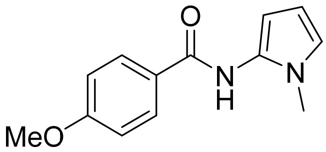

71%, R_f = 0.25 (PE:EA, 7:3); colorless solid; m.p. = 109-111°C; ^{1}H NMR (300 MHz, $CDCl_3$) δ 7.85 (d, *J* = 8.3 Hz, 2H), 7.47 (brs, 1H), 6.96 (d, *J* = 8.6 Hz, 2H), 6.57 (m, 1H), 6.11 (t, *J* = 3.3 Hz, 1H), 6.03 (m, 1H), 3.87 (s, 3H), 3.51 (s, 3H); ^{13}C NMR (75 MHz, $CDCl_3$) δ 167.03, 162.78, 129.36, 125.90, 125.05, 120.14, 114.06, 106.75, 104.09, 55.61, 33.13; HR-MS (ESI): m/z calculated for $C_{13}H_{15}N_2O_2$ 231,1128 obtained 231,1133.

Compound 3c: *N*-(1-Methyl-1H-pyrrol-2-yl)-1-naphthamide

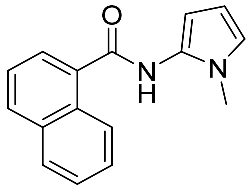

47%, R_f = 0.25 (PE:EA, 7:3); colorless solid; m.p. = 150-152°C; ^{1}H NMR (300 MHz, Aceton) δ 9.16 (brs, 1H), 8.47 – 8.40 (m, 1H), 8.09 – 7.96 (m, 2H), 7.89 (d, *J* = 6.8 Hz, 1H), 7.65 – 7.52 (m, 3H), 6.62 (s, 1H), 6.06 (s, 1H), 6.01 (t, *J* = 3.3 Hz, 1H), 3.65 (s, 3H); ^{13}C NMR (75 MHz, aceton) δ 169.30, 134.69, 131.41, 131.34, 130.72, 129.15, 127.74, 127.19, 127.05, 126.50, 126.47, 125.69, 119.95, 106.73, 103.80, 33.28; HR-MS (ESI): m/z calculated for $C_{16}H_{15}N_2O$ 251,1179 obtained 251,1178.

Compound 3d: 3,4,5-Trimethoxy-*N*-(1-methyl-1H-pyrrol-2-yl)benzamide

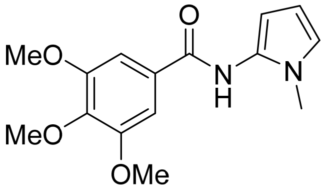

54%, R_f = 0.15 (PE:EA, 7:3); colorless solid; m.p. = 200-202°C; ^{1}H NMR (300 MHz, DMSO-d_6) δ 9.89 (s, 1H), 7.32 (s, 2H), 6.74 – 6.57 (m, 1H), 6.00 – 5.94 (m, 1H), 5.87 (dd, *J* = 3.4, 1.9 Hz, 1H), 3.85 (s, 6H), 3.72 (s, 3H), 3.42 (s, 3H); ^{13}C NMR (75 MHz, DMSO-d_6) δ 165.85, 152.69, 140.36, 128.78, 125.94, 119.44, 105.73, 105.19, 103.24, 60.14, 56.05, 32.62; HR-MS (ESI): m/z calculated for $C_{15}H_{19}N_2O_4$ 291,1339 obtained 291,1340.

Compound 3e: 4-Cyano-*N*-(1-methyl-1H-pyrrol-2-yl)benzamide

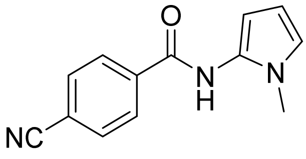

63%, R_f = 0.25 (PE:EA, 7:3); colorless solid; m.p. = 215-217°C; ^{1}H NMR (300 MHz, acetone-d_6) δ 9.40 (brs, 1H), 8.19 (d, *J* = 8.4 Hz, 2H), 7.95 (d, *J* = 8.5 Hz, 2H), 6.72 – 6.47 (m, 1H), 6.14 – 5.75 (m, 2H), 3.54 (s, 3H); ^{13}C NMR (75 MHz, acetone-d_6) δ 165.98, 164.36, 139.18, 133.28, 129.34, 120.24, 118.78, 115.79, 106.78, 104.02, 33.16; HR-MS (ESI): m/z calculated for $C_{13}H_{12}N_3O$ 226,0981 obtained 226,0975.

Compound 3f: Methyl 3-((1-methyl-1H-pyrrol-2-yl)carbamoyl)benzoate

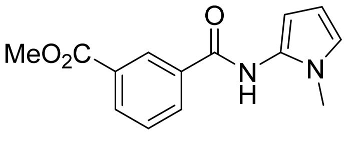

46%, R_f = 0.22 (PE:EA, 7:3); colorless solid; m.p. = 197-199°C; ^{1}H NMR (300 MHz, acetone-d_6) δ 9.42 (brs, 1H), 8.63 (s, 1H), 8.27 (m, 1H), 8.22 – 8.13 (m, 1H), 7.67 (t, *J* = 7.8 Hz, 1H), 6.66 – 6.46 (m, 1H), 5.98 (t, *J* = 3.3 Hz, 1H), 5.94 (m, 1H), 3.92 (s, 3H), 3.53 (s, 3H); ^{13}C NMR (75 MHz,

acetone-d_6) δ 166.66, 166.56, 135.64, 133.08, 132.87, 131.45, 129.77, 129.31, 126.72, 120.14, 106.70, 104.04, 52.62, 33.15; HR-MS (ESI): m/z calculated for $C_{14}H_{15}N_2O_3$ 259,1077 obtained 259,1079.

Compound 3g: 4-Chloro-*N*-(1-methyl-1H-pyrrol-2-yl)benzamide

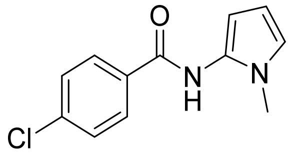

61%, R_f = 0.25 (PE:EA, 7:3); colorless solid; m.p. = 186-188°C; ^{1}H NMR (300 MHz, $CDCl_3$) δ 7.79 (d, *J* = 8.3 Hz, 2H), 7.73 (brs, 1H), 7.43 (d, *J* = 8.4 Hz, 2H), 6.56 (m, 1H), 6.10 (t, *J* = 3.2 Hz, 1H), 6.02 (m, 1H), 3.47 (s, 3H); ^{13}C NMR (75 MHz, $CDCl_3$) δ 166.63, 138.51, 132.02, 129.11, 128.92, 124.55, 120.28, 106.82, 104.22, 33.09; HR-MS (ESI): m/z calculated for $C_{12}H_{12}ClN_2O$ 235,0637 obtained 235,0633.

Compound 3h: *N*-(1-Methyl-1H-pyrrol-2-yl)thiophene-3-carboxamide

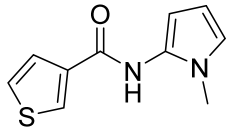

49%, R_f = 0.23 (PE:EA, 7:3); colorless solid; m.p. = 157-159°C; ^{1}H NMR (300 MHz, acetone-d_6) δ 9.04 (s, 1H), 8.32 – 8.15 (m, 1H), 7.64 (m, 1H), 7.56 (dd, *J* = 5.0, 2.9 Hz, 1H), 6.64 – 6.49 (m, 1H), 5.96 (t, *J* = 3.3 Hz, 1H), 5.88 (ddd, *J* = 3.6, 1.9, 0.6 Hz, 1H), 3.49 (s, 3H); ^{13}C NMR (75 MHz, acetone-d_6) δ 163.00, 138.32, 129.91, 127.90, 127.35, 126.77, 120.05, 106.65, 104.09, 33.09; HR-MS (ESI): m/z calculated for $C_{10}H_{11}N_2OS$ 207,0587 obtained 207,0588.

Compound 3j: *N*-(4-Acetyl-1-methyl-1H-pyrrol-2-yl)benzamide

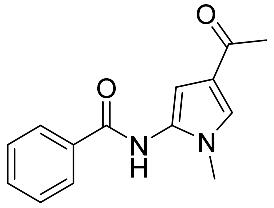

65%, R_f = 0.25 (PE:EA, 7:3); colorless solid; m.p. = 124-126°C; ^{1}H NMR (300 MHz, acetone-d_6) δ 9.85 (s, 1H), 8.08 (dd, *J* = 6.9, 1.6 Hz, 2H), 7.69 – 7.50 (m, 3H), 6.67 (d, *J* = 3.3 Hz, 1H), 6.53 (d, *J* = 3.3 Hz, 1H), 3.62 (s, 3H), 2.30 (s, 3H); ^{13}C NMR (75 MHz, acetone-d_6) δ 194.11, 167.06, 134.65, 133.09, 132.87, 129.59, 128.58, 120.55, 115.82, 108.75, 34.77, 27.70; HR-MS (ESI): m/z calculated for $C_{14}H_{14}N_2O_2$ 243,1128 obtained 243,1129.

Compound 3k: 4-Methoxy-*N*-(1-phenyl-1H-pyrrol-2-yl)benzamide

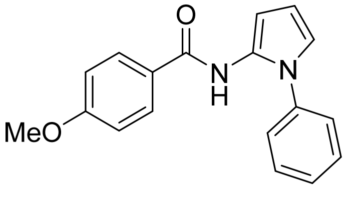

72%, R_f = 0.25 (PE:EA, 7:3); colorless solid; m.p. = 183-185°C; ^{1}H NMR (300 MHz, acetone-d$_6$) δ 8.95 (brs, 1H), 7.86 (d, *J* = 8.5 Hz, 2H), 7.46 – 7.35 (m, 4H), 7.33 – 7.25 (m, 1H), 6.97 (d, *J* = 8.8 Hz, 2H), 6.89 – 6.77 (m, 1H), 6.21 (t, *J* = 3.4 Hz, 1H), 6.17 (d, *J* = 1.1 Hz, 1H), 3.85 (s, 3H); ^{13}C NMR (75 MHz, acetone-d$_6$) δ 167.09, 163.37, 137.46, 130.21, 129.96, 127.61, 127.44, 125.69, 120.23, 114.44, 108.56, 107.01, 106.15, 55.82; HR-MS (ESI): m/z calculated for $C_{18}H_{17}N_2O_2$ 293,1285 obtained 293,1284.

Compound 3l: 4-Methoxy-*N*-(1H-pyrrol-2-yl)benzamide

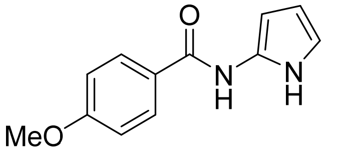

69%, R_f = 0.10 (PE:EA, 7:3); colorless solid; m.p. = 179-181°C; ^{1}H NMR (300 MHz, DMSO-d$_6$) δ 10.75 (brs, 1H), 10.42 (brs, 1H), 7.94 (d, *J* = 8.8 Hz, 2H), 7.05 (d, *J* = 8.9 Hz, 2H), 6.64 – 6.21 (m, 1H), 5.93 (dd, *J* = 5.6, 2.6 Hz, 1H), 5.83 (d, *J* = 1.4 Hz, 1H), 3.83 (s, 3H); ^{13}C NMR (75 MHz, DMSO-d$_6$) δ 163.44, 161.86, 129.32, 128.13, 126.20, 113.71, 112.63, 106.30, 96.06, 55.45; HR-MS (ESI): m/z calculated for $C_{12}H_{13}N_2O_2$ 217,0972 obtained 217,0975.

Compound 3m: 4-Methoxy-*N*-(1-(4-methoxybenzoyl)-1H-pyrrol-2-yl)benzamide

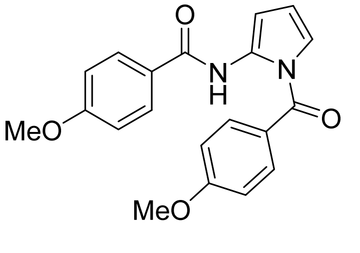

64%, R_f = 0.30 (PE:EA, 7:3); colorless solid; m.p. = 151-153°C; ^{1}H NMR (300 MHz, $CDCl_3$) δ 11.09 (s, 1H), 7.92 (d, *J* = 8.9 Hz, 2H), 7.77 (d, *J* = 8.9 Hz, 2H), 7.04 – 6.92 (m, 5H), 6.68 (dd, *J* = 3.6, 1.7 Hz, 1H), 6.24 (t, *J* = 3.6 Hz, 1H), 3.90 (s, 3H), 3.86 (s, 3H); ^{13}C NMR (75 MHz, $CDCl_3$) δ 171.58, 163.45, 163.12, 162.65, 132.22, 129.11, 126.26, 125.47, 117.48, 114.11, 114.03, 111.98, 111.97, 101.60, 55.72, 55.57; HR-MS (ESI): m/z calculated for $C_{20}H_{19}N_2O_4$ 351,1339 obtained 351,1339.

Compound 3n: 4-Methoxy-*N*-(5-methylfuran-2-yl)benzamide

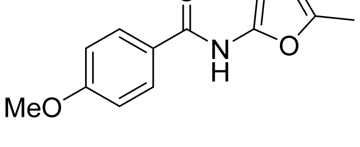

49%, R_f = 0.25 (PE:EA, 7:3); colorless solid; m.p. = 122-124°C; ^{1}H NMR (300 MHz, acetone-d$_6$) δ 9.89 (brs, 1H), 8.03 (d, *J* = 8.9 Hz, 2H), 7.19 – 6.92 (m, *J* = 8.9 Hz, 2H), 6.24 (d, *J* = 3.1 Hz, 1H), 6.00 (d, *J* = 2.9, 1H), 3.88 (s, 3H), 2.21 (s, 3H); ^{13}C NMR (75 MHz, acetone-d$_6$) δ 163.61, 163.49, 146.18,

145.31, 130.28, 126.95, 114.50, 107.61, 96.68, 55.83, 13.20; HR-MS (ESI): m/z calculated for $C_{13}H_{14}NO_3$ 232,0968 obtained 232,0969.

Compound 3o: *N*-(Benzofuran-2-yl)-4-methoxybenzamide[9]

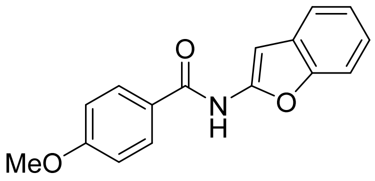

15%, R_f = 0.25 (PE:EA, 7:3); colorless solid; m.p. = 145-147°C; ^{1}H NMR (300 MHz, acetone-d$_6$) δ 7.94 (d, *J* = 9.0 Hz, 2H), 7.59 – 7.52 (m, 1H), 7.36 – 7.27 (m, 1H), 7.05 – 6.94 (m, 3H), 6.89 (d, *J* = 8.2 Hz, 1H), 6.73 (d, *J* = 6.7 Hz, 1H), 6.27 (d, *J* = 6.7 Hz, 1H), 3.86 (s, 3H); ^{13}C NMR (75 MHz, acetone-d$_6$) δ 164.76, 163.87, 163.63, 130.57, 129.00, 128.52, 126.51, 126.06, 124.02, 123.34, 120.88, 114.66, 110.78, 90.47, 55.91; HR-MS (ESI): m/z calculated for $C_{16}H_{14}NO_4$ 268,0968 obtained 268,0968.

Compound 3p: *N*-(1-(Dimethylamino)-1H-pyrrol-2-yl)-4-methoxybenzamide

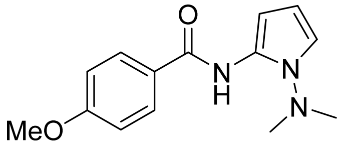

59% (75mg), R_f = 0.25 (PE:EA, 7:3); colorless solid; m.p. = 117-119°C; ^{1}H NMR (300 MHz, acetone-d$_6$) δ 9.05 (brs, 1H), 7.91 (d, *J* = 8.8 Hz, 2H), 7.04 (d, *J* = 8.8 Hz, 2H), 6.90 (dd, *J* = 3.3, 1.9 Hz, 1H), 6.23 (dd, *J* = 3.8, 1.8 Hz, 1H), 6.02 (t, *J* = 3.6 Hz, 1H), 3.87 (s, 3H), 2.82 (s, 3H); ^{13}C NMR (75 MHz, acetone-d$_6$) δ 163.24, 162.95, 129.79, 127.75, 127.43, 114.58, 108.53, 106.54, 94.59, 55.82, 47.72; HR-MS (ESI): m/z calculated for $C_{14}H_{18}N_3O_2$ 260,1394 obtained 260,1399.

Compound 3q: *N*-(4-Acetyl-1-methyl-1H-pyrrol-2-yl)-4-chlorobenzamide

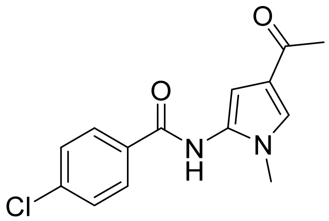

46%, R_f = 0.25 (PE:EA, 7:3); colorless solid; m.p. = 163-165°C; ^{1}H NMR (300 MHz, acetone-d$_6$) δ 9.85 (brs, 1H), 8.09 (d, *J* = 8.6 Hz, 2H), 7.61 (d, *J* = 8.6 Hz, 2H), 6.68 (d, *J* = 3.3 Hz, 1H), 6.53 (d, *J* = 3.3 Hz, 1H), 3.61 (s, 3H), 2.30 (s, 3H); ^{13}C NMR (75 MHz, acetone-d$_6$) δ 194.04, 166.15, 138.68, 133.38, 132.34, 130.41, 129.72, 120.63, 116.05, 108.82, 34.65, 27.73; HR-MS (ESI): m/z calculated for $C_{14}H_{14}ClN_2O_2$ 277,0738 obtained 277,0741.

Compound 3r: 4-Chloro-*N*-(1-phenyl-1H-pyrrol-2-yl)benzamide

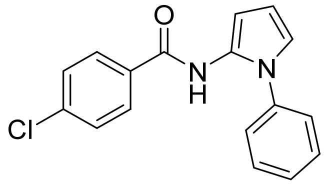

88%, R_f = 0.38 (PE:EA, 7:3); colorless solid; m.p. = 195-197°C; ^{1}H NMR (300 MHz, acetone-d$_6$) δ 9.22 (s, 1H), 7.88 (d, *J* = 8.5 Hz, 3H), 7.49 (d, *J* = 8.5 Hz, 3H), 7.42 (d, *J* = 4.4 Hz, 6H), 7.31 (dd, *J* = 8.5, 4.3 Hz, 2H), 6.92 – 6.82 (m, 2H), 6.27 – 6.17 (m, 3H); ^{13}C NMR (75 MHz, acetone-d$_6$) δ 166.66, 140.09, 138.07, 133.95, 130.16, 130.04, 129.46, 127.75, 126.24, 125.70, 120.50, 108.66, 107.07; HR-MS (ESI): m/z calculated for $C_{17}H_{14}ClN_2O$ 277,0789 obtained 277,0788.

Compound 3s: 4-Chloro-*N*-(1-methyl-1H-indol-2-yl)benzamide

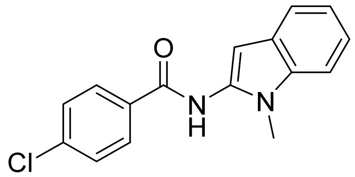

59%, R_f = 0.25 (PE:EA, 7:3); colorless solid; m.p. = 190-192°C; ^{1}H NMR (300 MHz, acetone-d$_6$) δ 9.74 (brs, 1H), 8.09 (d, J = 8.5 Hz, 2H), 7.59 (d, J = 8.6 Hz, 2H), 7.52 (d, J = 7.9 Hz, 2H), 7.37 (d, J = 8.2 Hz, 1H), 7.19 – 7.10 (m, 1H), 7.09 – 6.99 (m, 1H), 6.50 (s, 1H), 3.72 (s, 3H); ^{13}C NMR (75 MHz, acetone-d$_6$) δ 166.01, 143.07, 138.36, 135.90, 133.74, 130.46, 129.53, 127.92, 121.76, 120.77, 120.30, 110.05, 95.58, 29.53.
; HR-MS (ESI): m/z calculated for $C_{16}H_{14}ClN_2O$ 287,0764 obtained 287,0764.

Compound 3t: 4-Chloro-*N*-(5-methylthiophen-2-yl)benzamide

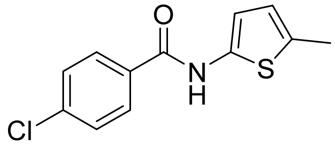

35%, R_f = 0.29 (PE:EA, 7:3); colorless solid; m.p. = 167-169°C; ^{1}H NMR (300 MHz, acetone-d$_6$) δ 10.64 (brs, 1H), 8.01 (d, *J* = 8.6 Hz, 2H), 7.55 (d, *J* = 8.6 Hz, 2H), 6.67 (d, *J* = 3.7 Hz, 1H), 6.54 (d, *J* = 3.6 Hz, 1H), 2.40 (s, 3H); ^{13}C NMR (75 MHz, acetone-d$_6$) δ 162.85, 138.45, 138.13, 133.29, 131.78, 130.09, 129.54, 122.56, 112.66, 14.74; HR-MS (ESI): m/z calculated for $C_{12}H_{11}ClNOS$ 252,0244 obtained 252,0249.

Compound 3u: 4-Cyano-*N*-(1,3-dimethyl-1H-indol-2-yl)benzamide

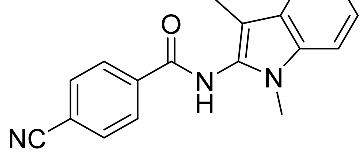

61%, R_f = 0.22 (PE:EA, 7:3); colorless solid; m.p. = 199-201°C; ^{1}H NMR (300 MHz, acetone-d$_6$) δ 9.78 (brs, 1H), 8.28 (d, *J* = 8.5 Hz, 2H), 7.99 (d, *J* = 8.5 Hz, 2H), 7.52 (d, *J* = 7.9 Hz, 1H), 7.36 (d, *J* =

8.2 Hz, 1H), 7.23 – 7.11 (m, 1H), 7.06 (dd, *J* = 11.0, 4.0 Hz, 1H), 3.66 (s, 3H), 2.21 (s, 3H); ^{13}C NMR (75 MHz, acetone-d$_6$) δ 166.16, 138.85, 135.82, 133.39, 130.23, 129.49, 127.92, 122.30, 119.62, 119.29, 118.76, 116.06, 109.98, 104.28, 30.35, 8.51; HR-MS (ESI): m/z calculated for $C_{18}H_{16}N_3O$ 290,1288 obtained 290,1284.

Compound 3v: 4-Cyano-*N*-(5-methylfuran-2-yl)benzamide

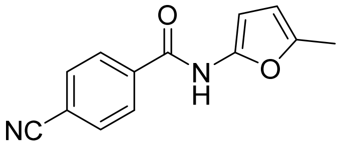

44%, R$_f$ = 0.25 (PE:EA, 7:3); colorless solid; m.p. = 139-141°C; ^{1}H NMR (300 MHz, CDCl$_3$) δ 8.42 (brs, 1H), 7.97 (d, *J* = 8.4 Hz, 2H), 7.76 (d, *J* = 8.3 Hz, 2H), 6.33 (d, *J* = 2.7 Hz, 1H), 5.99 (d, *J* = 2.1 Hz, 1H), 2.23 (s, 3H); ^{13}C NMR (75 MHz, CDCl$_3$) δ 161.78, 146.13, 142.85, 137.43, 132.74, 127.98, 117.99, 115.71, 107.42, 97.68, 13.42; HR-MS (ESI): m/z calculated for $C_{12}H_{12}N_2O_2$ 227,0742 obtained 227,0744.

Compound 3w: *N*-(1-Phenyl-1H-pyrrol-2-yl)cinnamamide

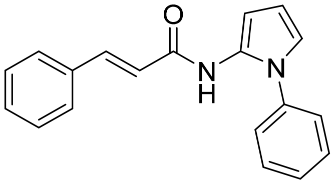

55%, R$_f$ = 0.25 (PE:EA, 7:3); colorless solid; m.p. = 213-215°C; ^{1}H NMR (300 MHz, acetone-d$_6$) δ 8.80 (brs, 1H), 7.58 (dd, *J* = 9.8, 5.8 Hz, 2H), 7.49 – 7.30 (m, 9H), 6.84 – 6.70 (m, 2H), 6.27 (d, *J* = 1.6 Hz, 1H), 6.20 (t, *J* = 3.4 Hz, 1H); ^{13}C NMR (75 MHz, acetone-d$_6$) δ 165.42, 143.01, 141.64, 139.89, 135.93, 130.52, 130.11, 129.74, 128.61, 127.80, 125.87, 121.88, 119.81, 108.64, 105.56; HR-MS (ESI): m/z calculated for $C_{19}H_{17}N_2O$ 289,1335 obtained 289,1336.

Compound 3x: 4-Methyl-*N*-(1-methyl-1H-pyrrol-2-yl)benzenesulfonamide

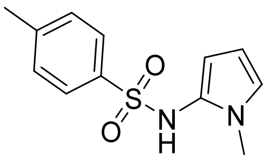

52%, R$_f$ = 0.23 (PE:EA, 7:3); colorless solid; m.p. = 157-159°C; ^{1}H NMR (300 MHz, acetone-d$_6$) δ 9.04 (s, 1H), 8.32 – 8.15 (m, 1H), 7.64 (m, 1H), 7.56 (dd, *J* = 5.0, 2.9 Hz, 1H), 6.64 – 6.49 (m, 1H), 5.96 (t, *J* = 3.3 Hz, 1H), 5.88 (ddd, *J* = 3.6, 1.9, 0.6 Hz, 1H), 3.49 (s, 3H); ^{13}C NMR (75 MHz, acetone-d$_6$) δ 163.00, 138.32, 129.91, 127.90, 127.35, 126.77, 120.05, 106.65, 104.09, 33.09; HR-MS (ESI): m/z calculated for $C_{12}H_{15}N_2O_2S$ 251,0849 obtained 251,0850.

Compound 6: 4-Chloro-*N*-(3-methyl-1-phenyl-1H-indol-2-yl)benzamide

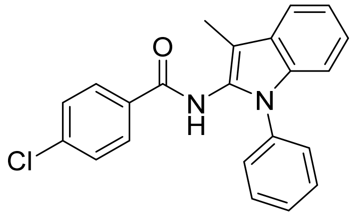

65%, R_f = 0.70 (PE:EA, 7:3); colorless solid; m.p. = 213-215°C; ^{1}H NMR (300 MHz, acetone-d_6) δ 9.51 (brs, 1H), 7.91 (d, *J* = 8.5 Hz, 2H), 7.65 – 7.56 (m, 1H), 7.53 – 7.33 (m, 7H), 7.24 – 7.10 (m, 3H), 2.27 (s, 3H); ^{13}C NMR (75 MHz, acetone-d_6) δ 166.58, 143.84, 138.24, 137.95, 136.08, 133.63, 131.90, 130.22, 130.14, 129.49, 128.47, 128.15, 127.98, 123.20, 120.69, 119.62, 110.67, 108.17, 8.55; HR-MS (ESI): m/z calculated for $C_{22}H_{18}ClN_2O$ 363,1081 obtained 363,1084.

Compound 8: 2-(4-Chlorophenyl)-4-methyl-4-phenyl-4,5-dihydrooxazole

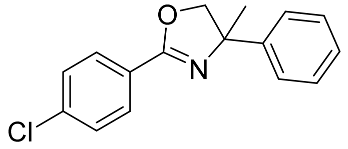

55%, R_f = 0.35 (PE:EA, 7:3); colorless oil, ^{1}H NMR (400 MHz, acetone-d_6) δ 8.08 (d, *J* = 8.7 Hz, 2H), 7.58 (d, *J* = 8.8 Hz, 2H), 7.54 – 7.49 (m, 2H), 7.46 – 7.39 (m, 2H), 7.38 – 7.30 (m, 1H), 4.20 (d, *J* = 14.9 Hz, 1H), 4.11 (d, *J* = 14.9 Hz, 1H), 1.82 (s, 3H); ^{13}C NMR (101 MHz, acetone-d_6) δ 162.04, 146.75, 137.71, 130.53, 129.55, 129.41, 128.18, 128.10, 125.11, 87.84, 69.76, 28.48; HR-MS (ESI): m/z calculated for $C_{16}H_{15}ClNO$ 273,0869 obtained 273,0875.

Compound 9: 2-(4-Methoxyphenyl)-3a,8b-dihydrobenzofuro[2,3-d]oxazole

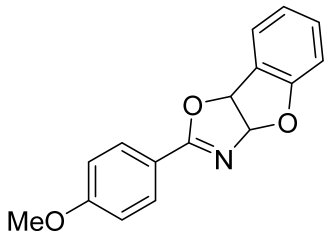

35%, R_f = 0.7 (PE:EA, 7:3); colorless solid; m.p. = 150-152°C; ^{1}H NMR (300 MHz, acetone-d_6) δ 7.94 (d, *J* = 9.0 Hz, 2H), 7.63 – 7.48 (m, 1H), 7.39 – 7.25 (m, 1H), 7.06 – 6.94 (m, 3H), 6.89 (d, *J* = 8.2 Hz, 1H), 6.73 (d, *J* = 6.7 Hz, 1H), 6.27 (d, *J* = 6.7 Hz, 1H), 3.86 (s, 3H); ^{13}C NMR (75 MHz, acetone-d_6) δ 167.50, 163.93, 160.01, 132.48, 131.46, 127.85, 124.89, 121.95, 120.01, 114.77, 111.48, 105.55, 83.63, 55.85; HR-MS (ESI): m/z calculated for $C_{16}H_{14}NO_3$ 269,1001 obtained 269,1001.

3) Proton and carbon NMR spectra of prepared compounds

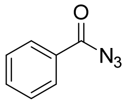

Compound 1a: benzoyl azide

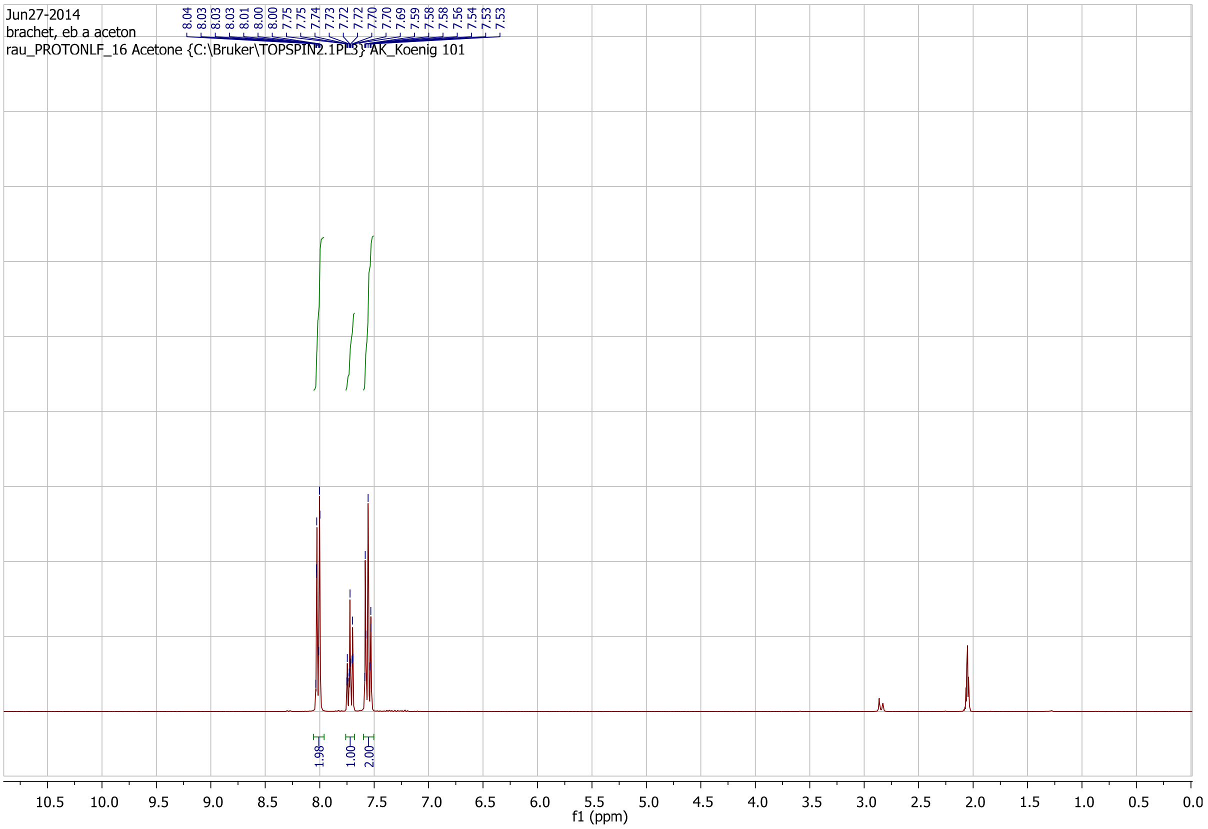

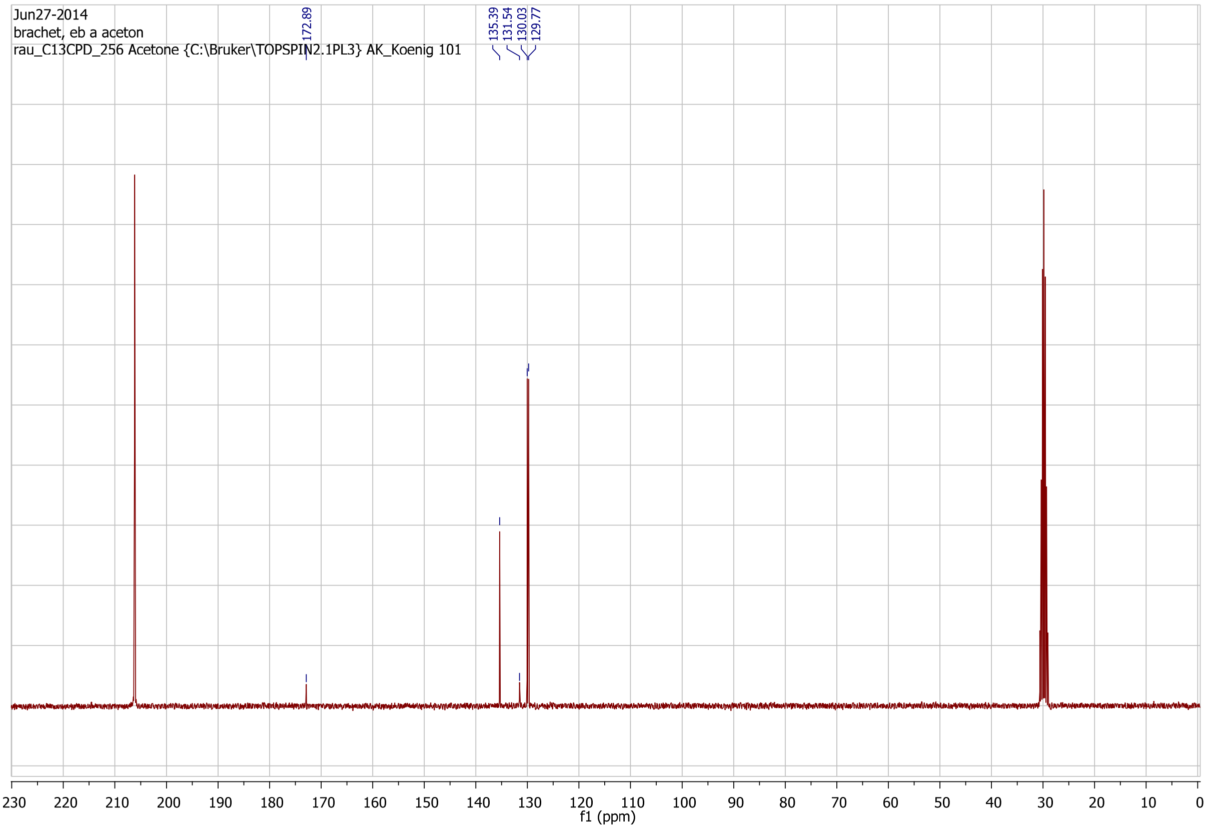

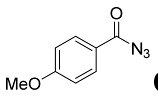

Compound 1b: 4-methoxybenzoyl azide

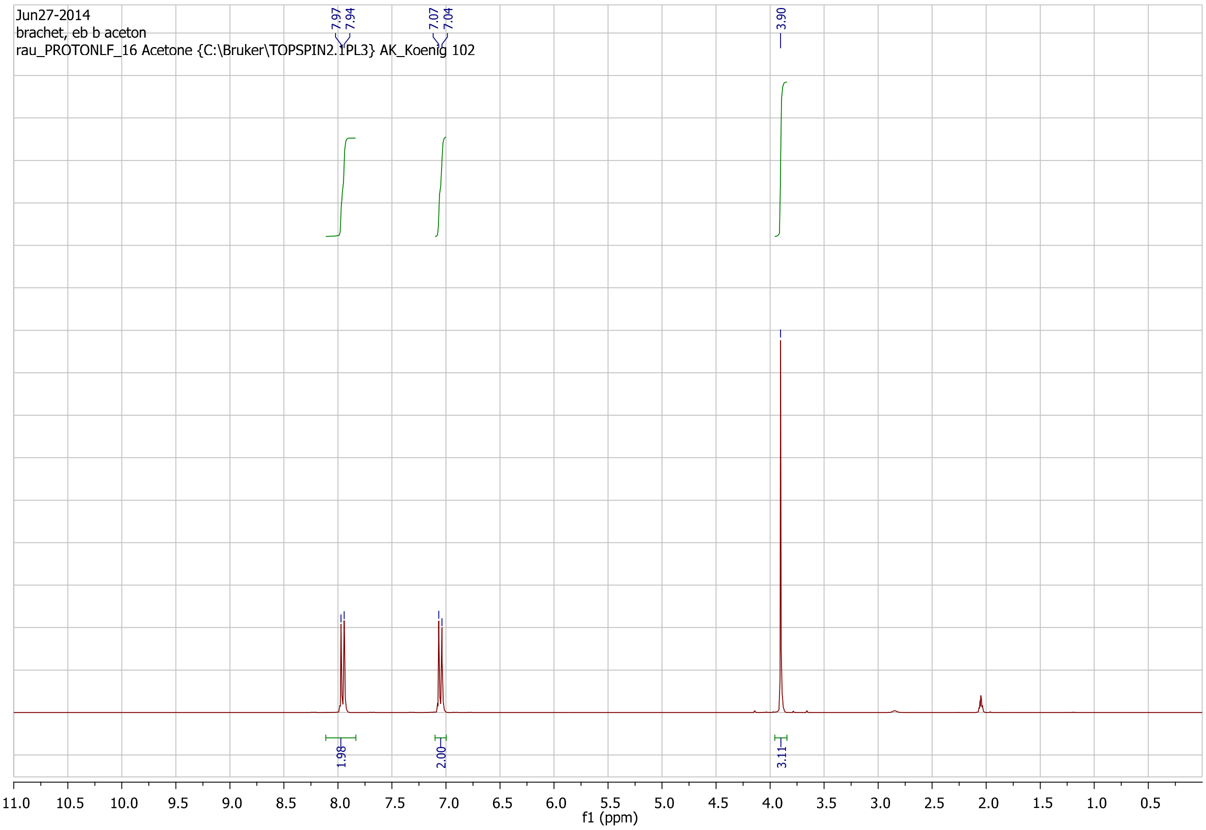

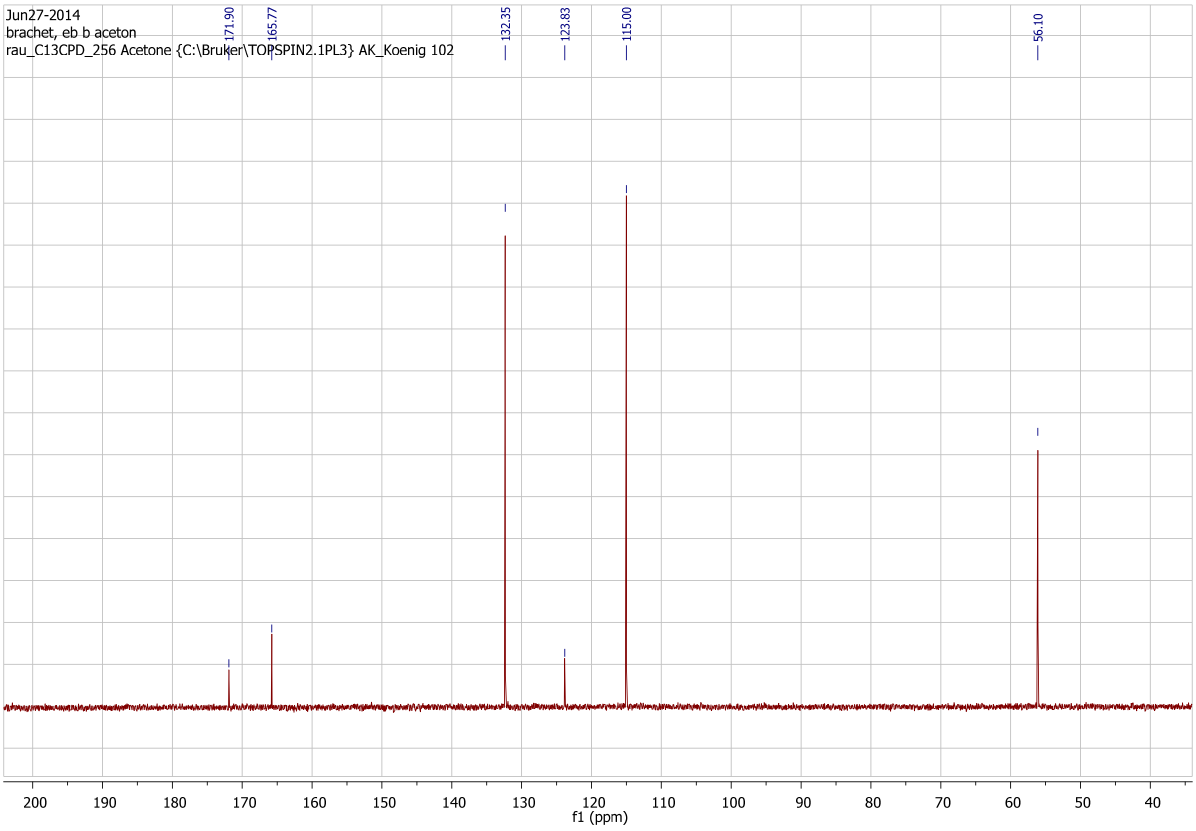

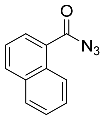

Compound 1c: 1-naphthoyl azide

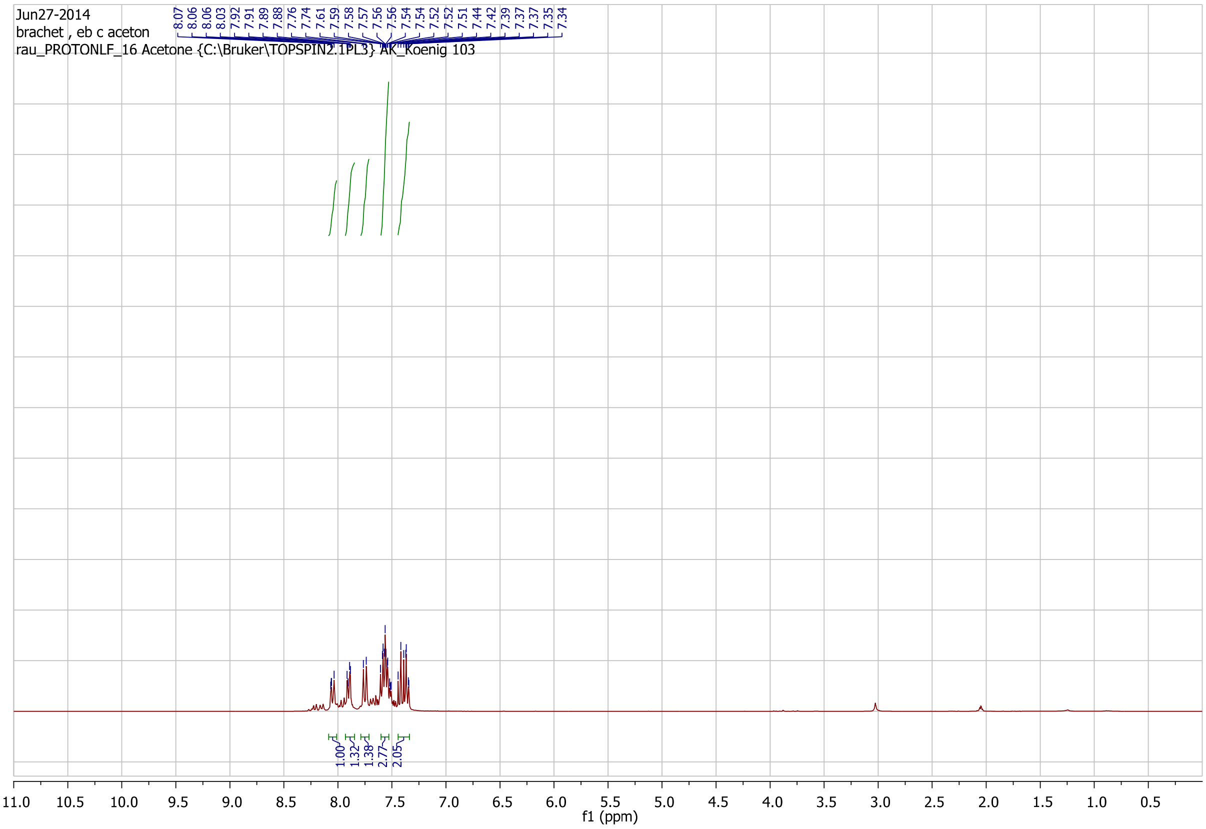

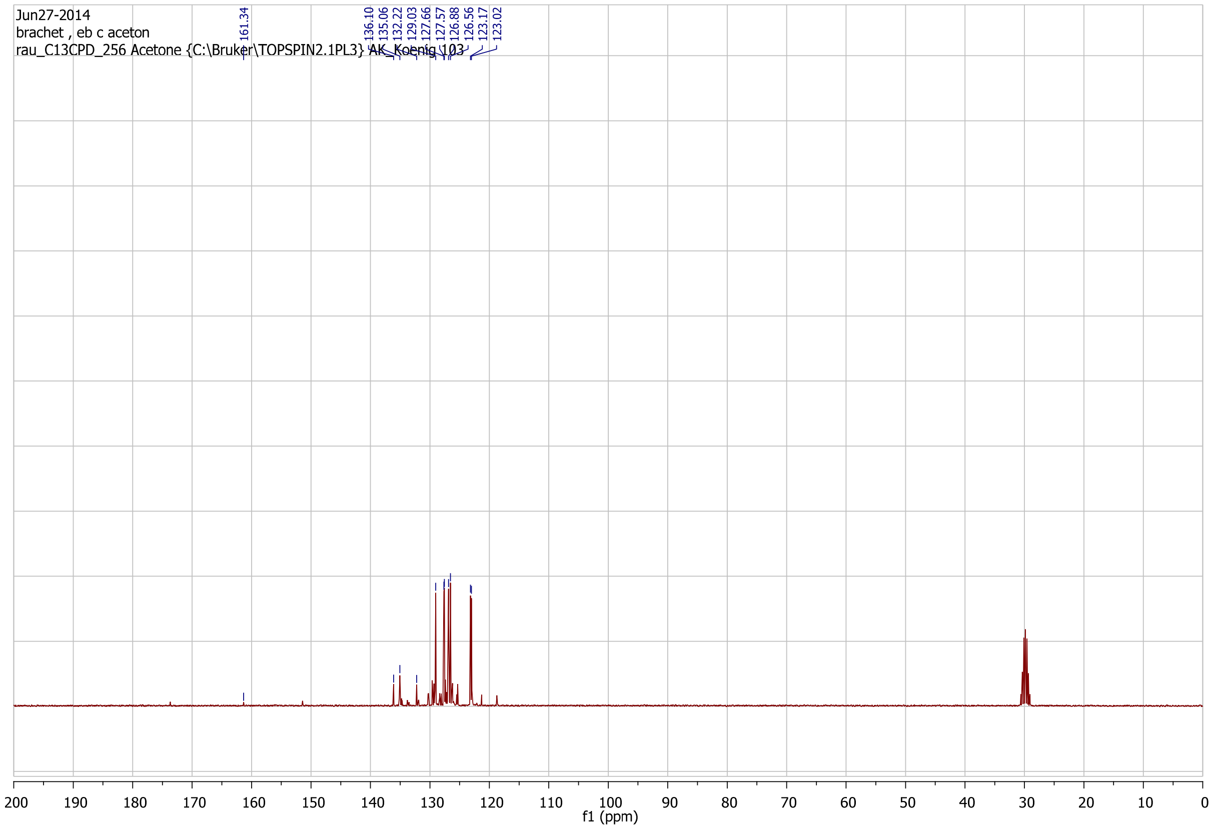

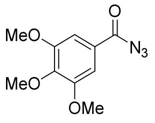

Compound 1d: 3,4,5-trimethoxybenzoyl azide

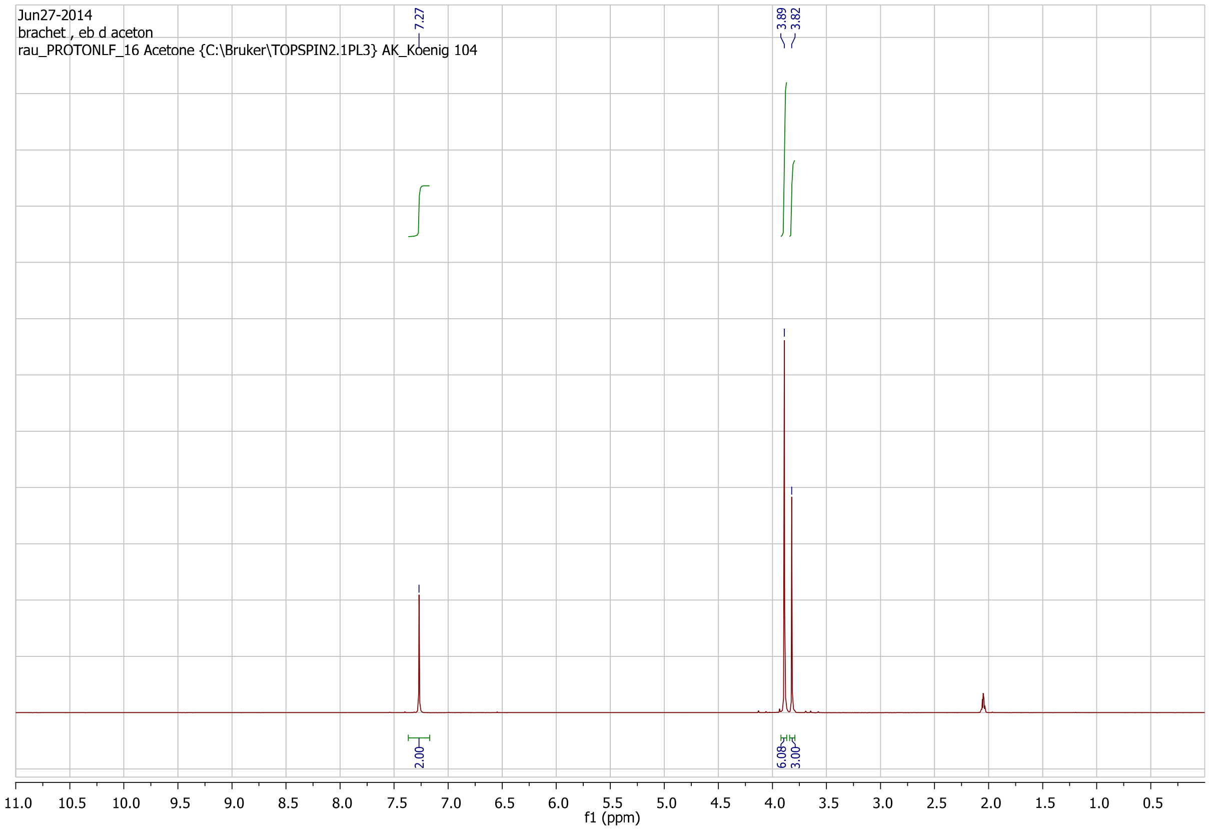

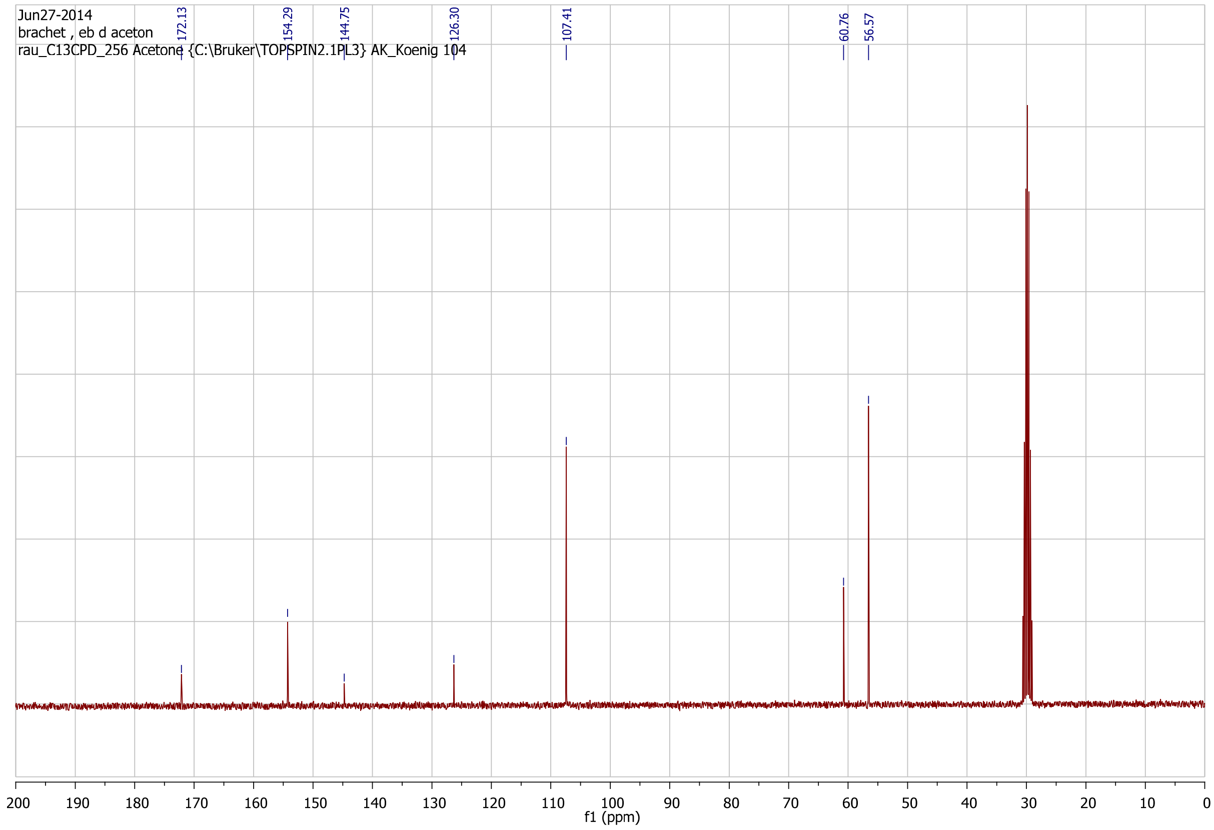

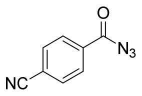

Compound 1e: 4-cyanobenzoyl azide

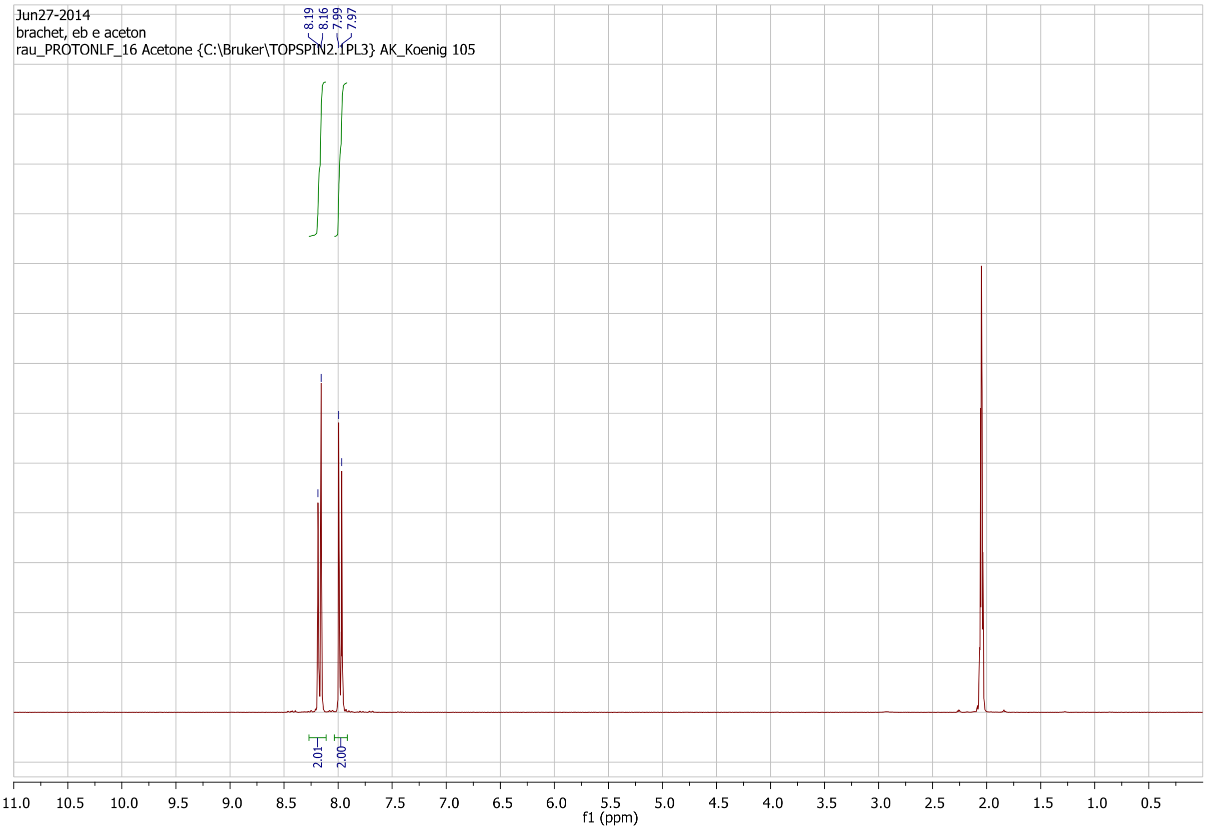

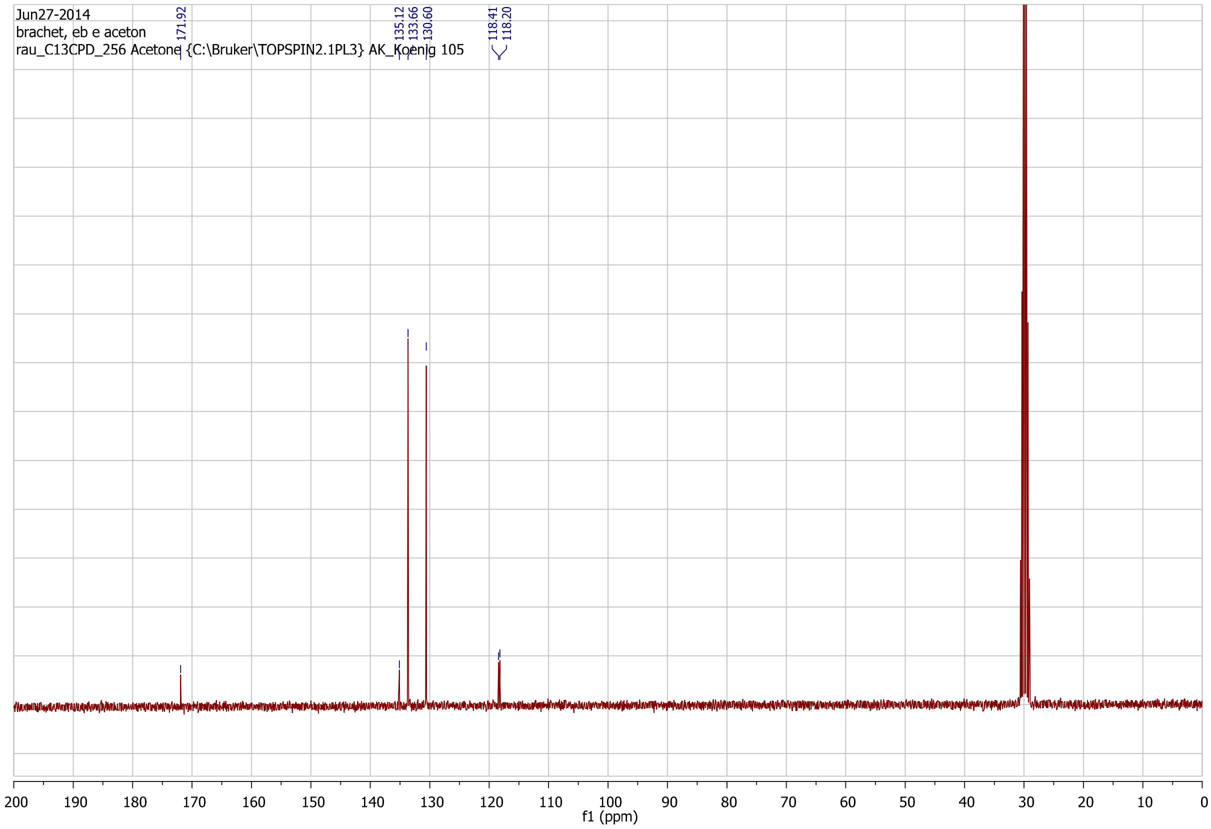

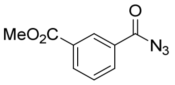

Compound 1f: methyl 3-(azidocarbonyl)benzoate

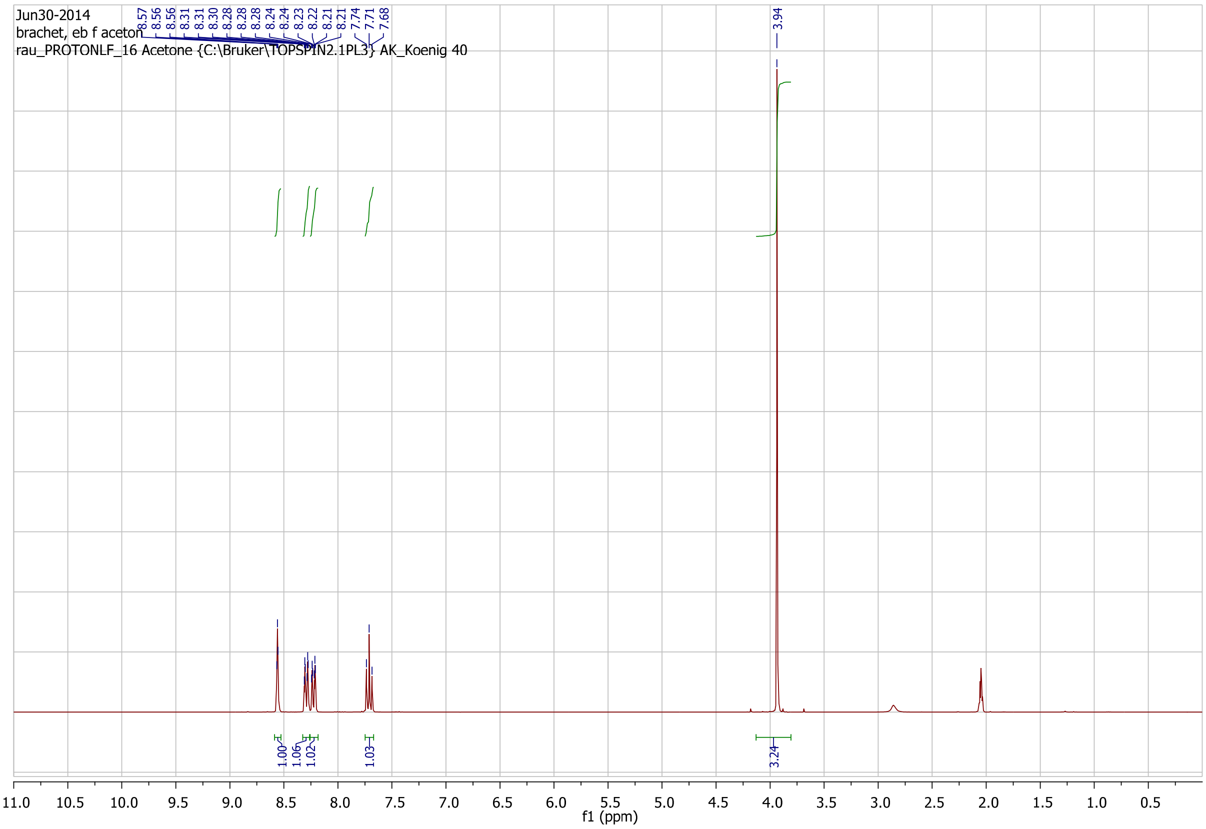

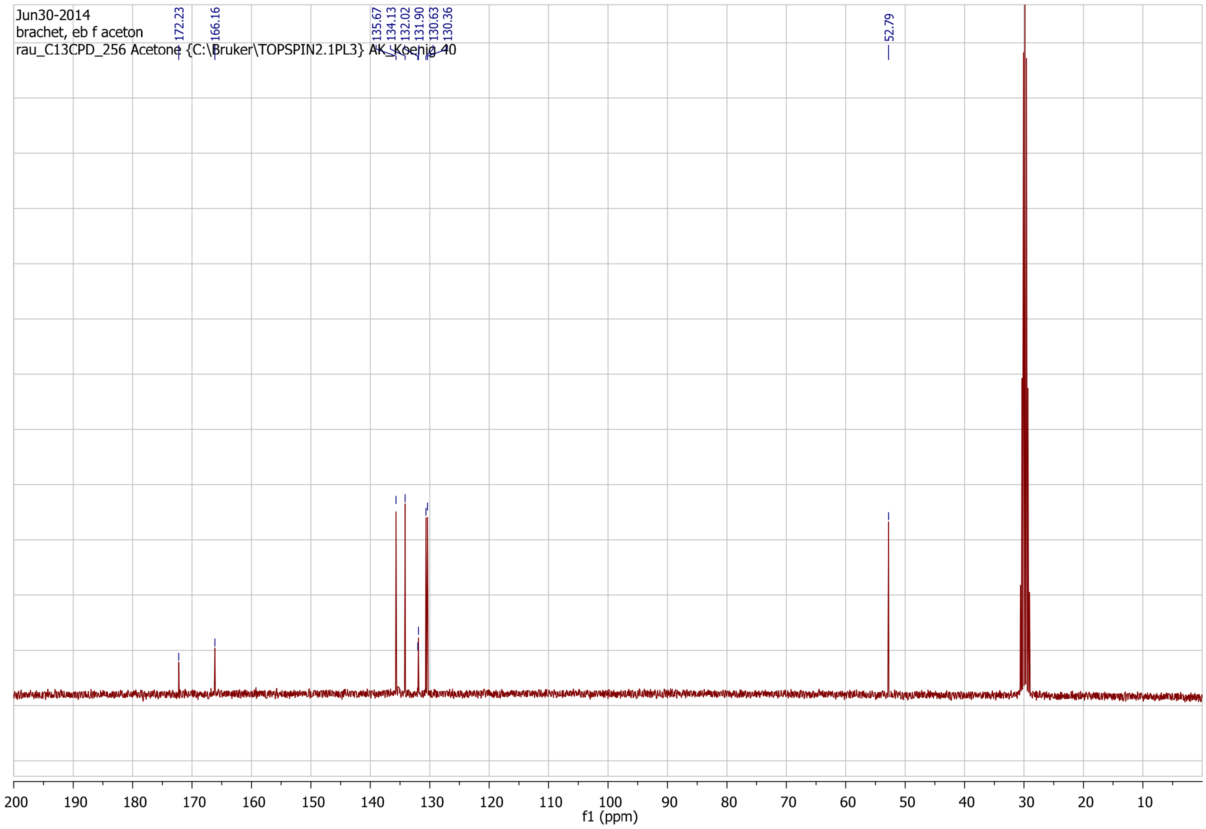

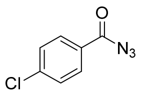

Compound 1g: 4-chlorobenzoyl azide

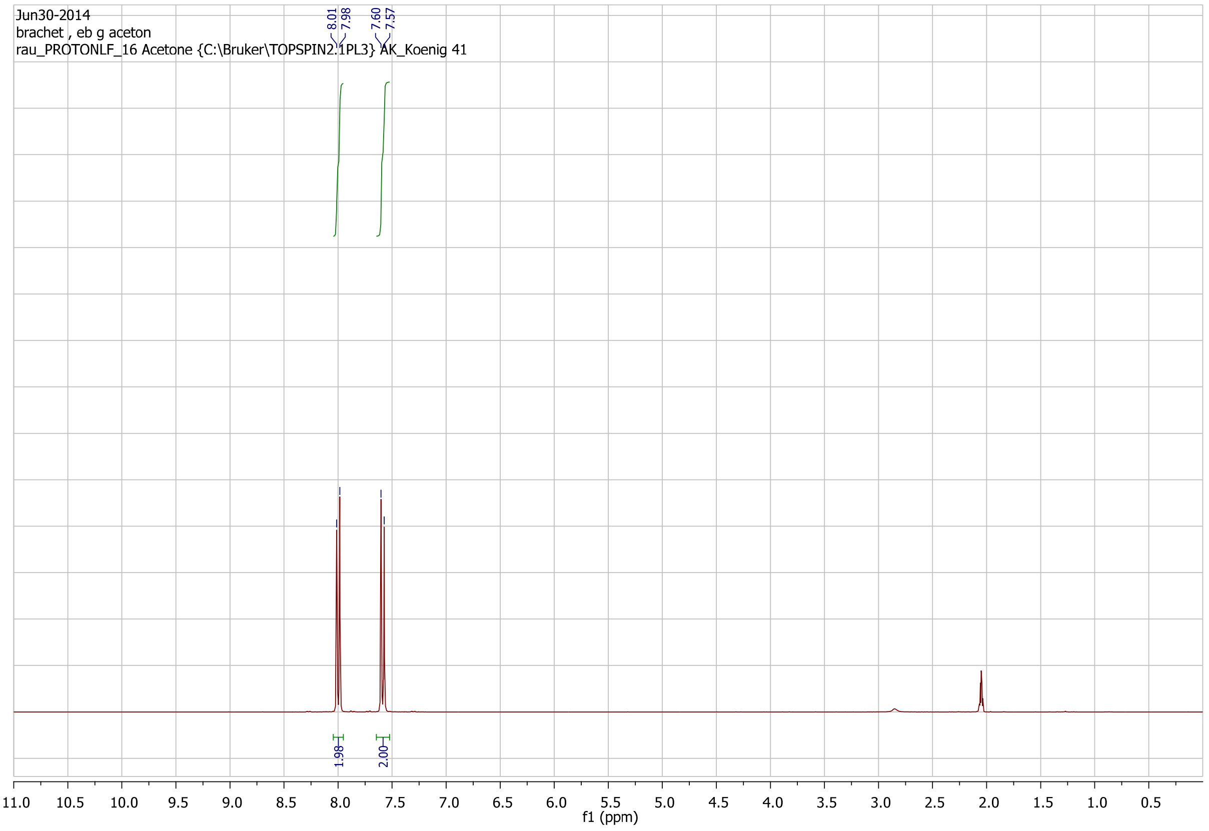

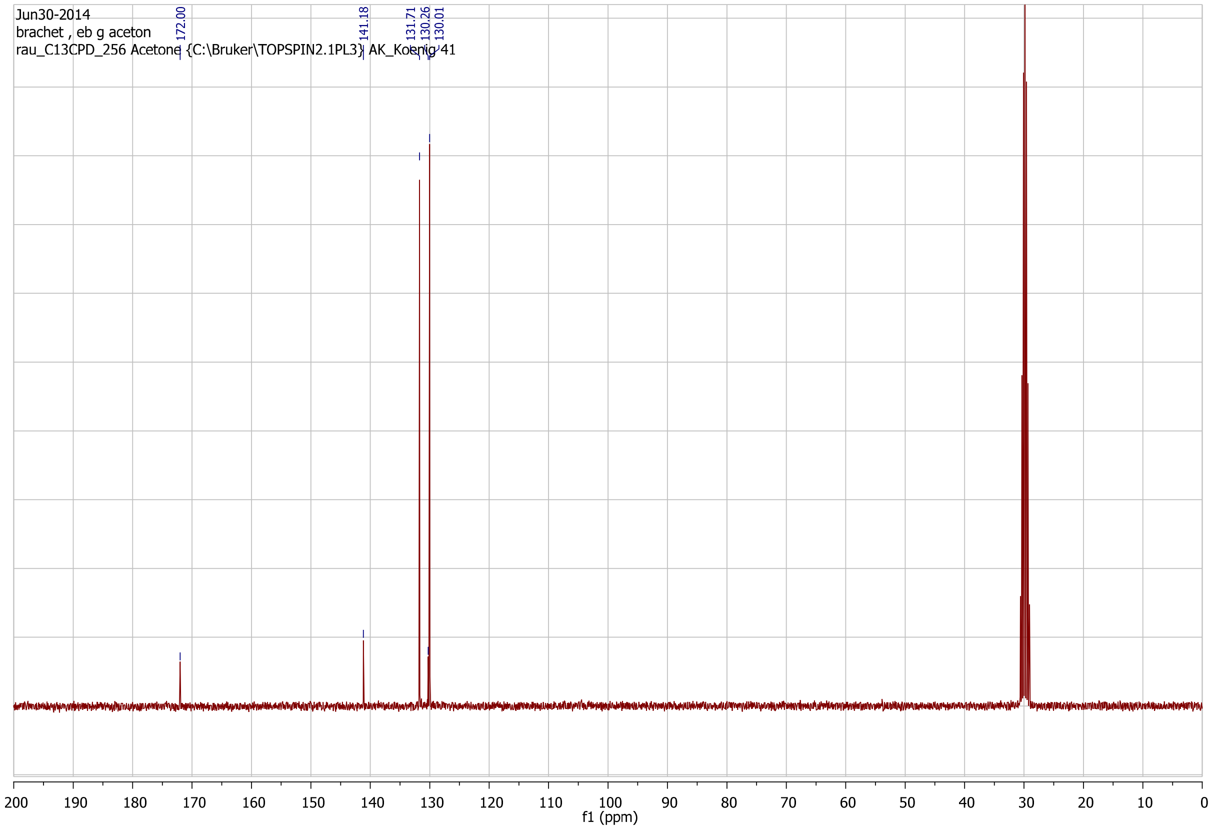

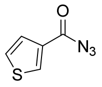

Compound 1h: thiophene-3-carbonyl azide

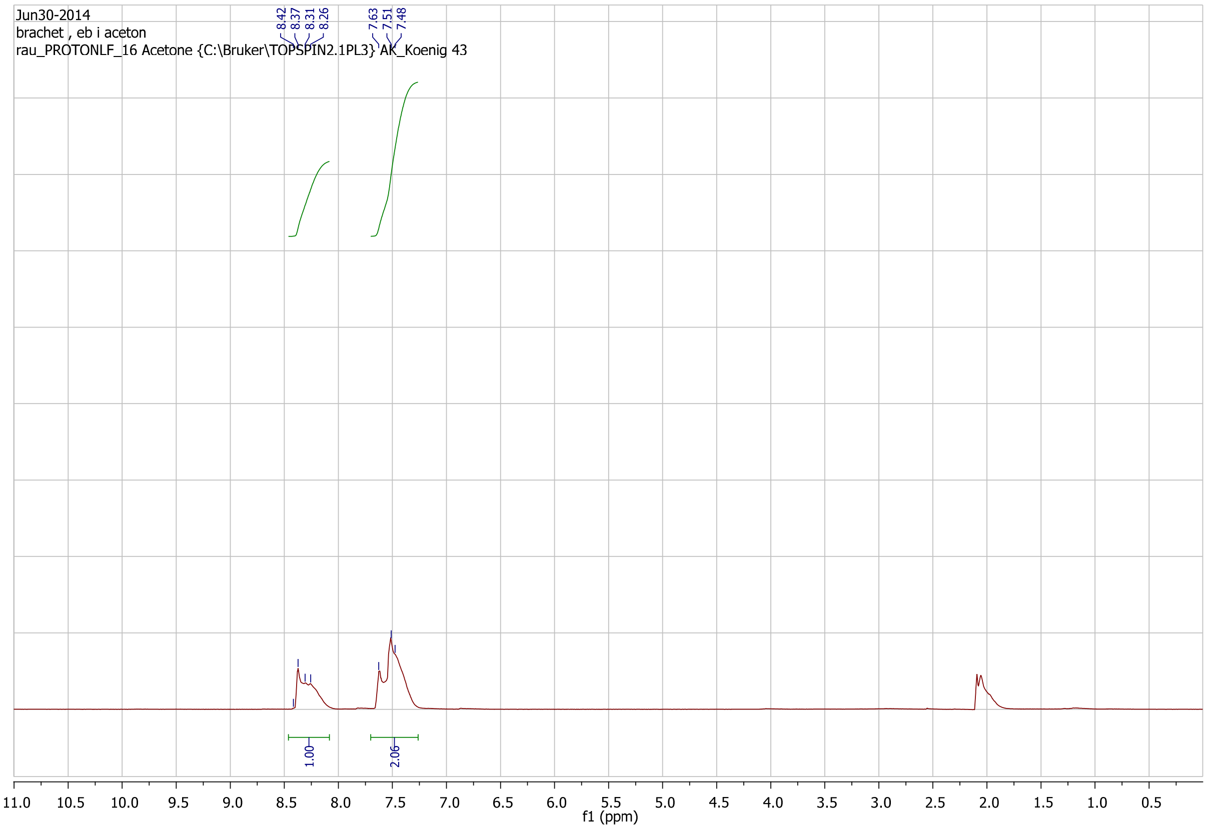

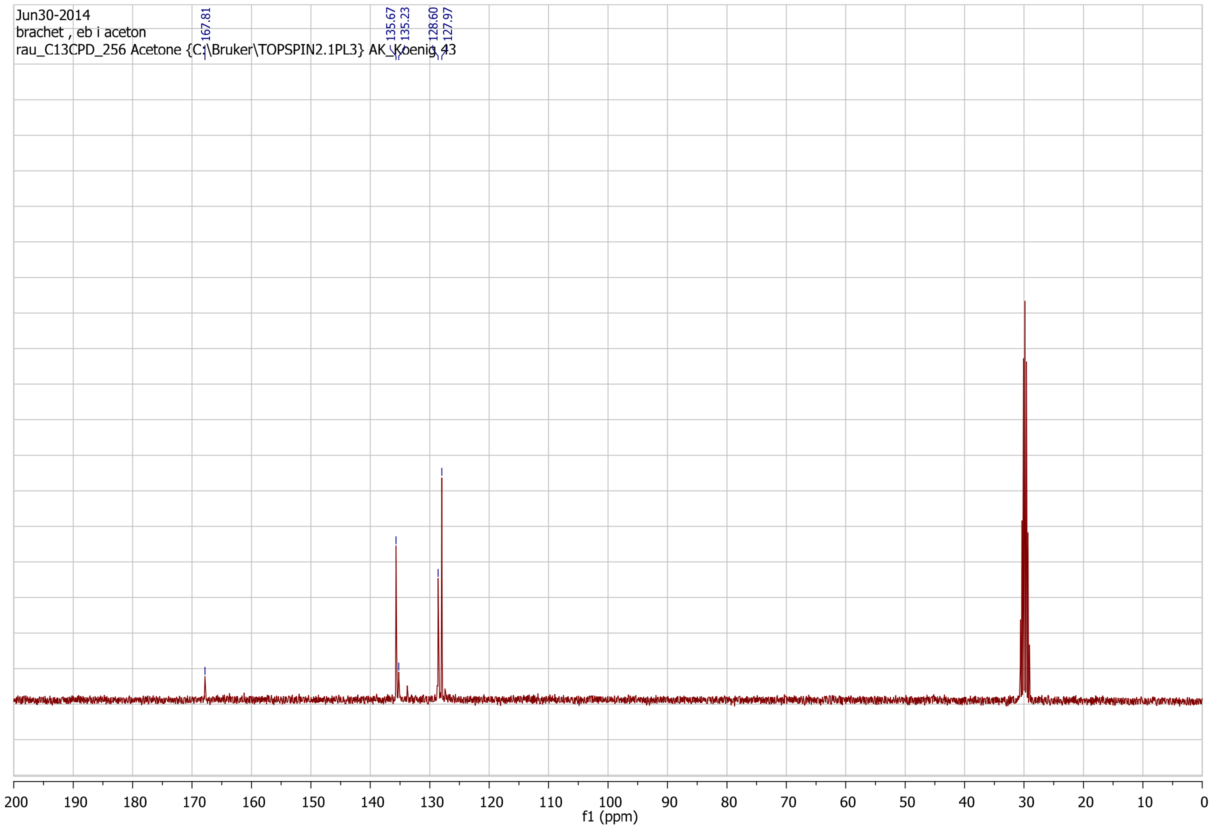

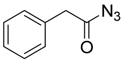

Compound 1i: 2-phenylacetyl azide

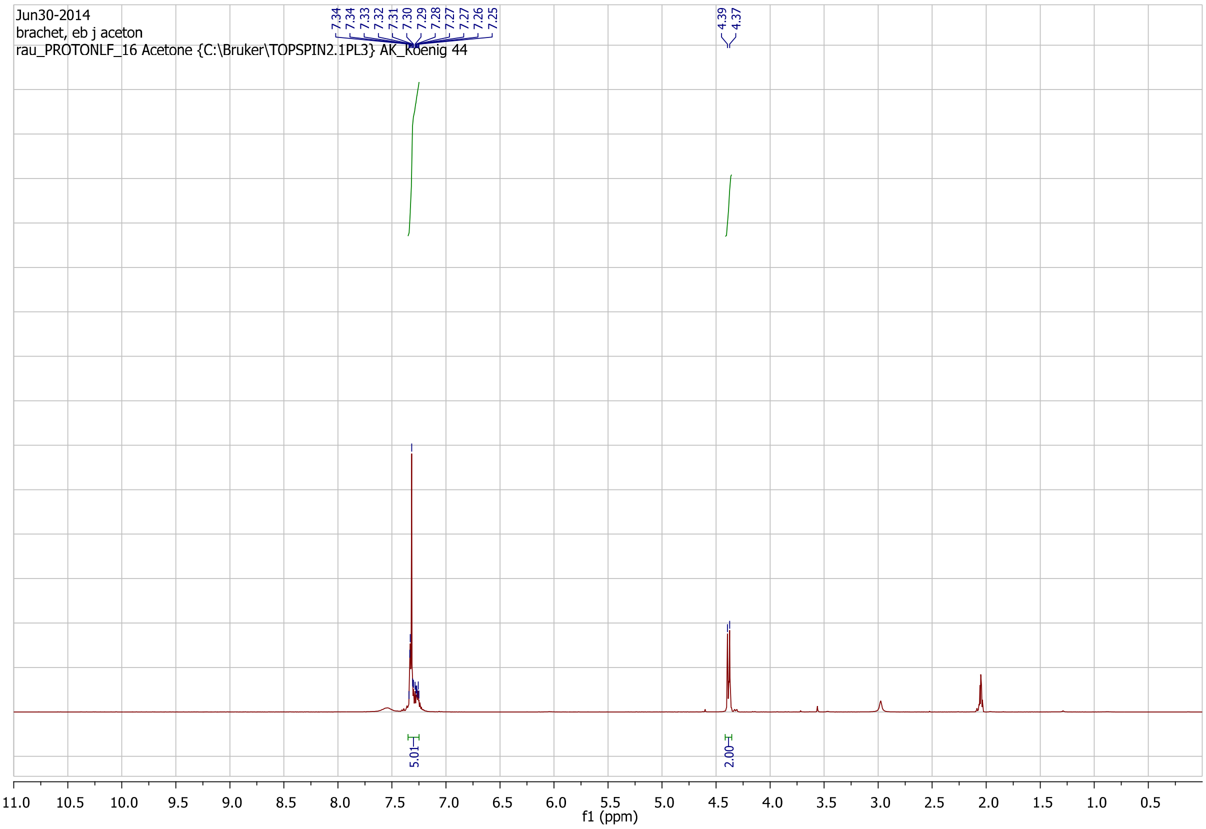

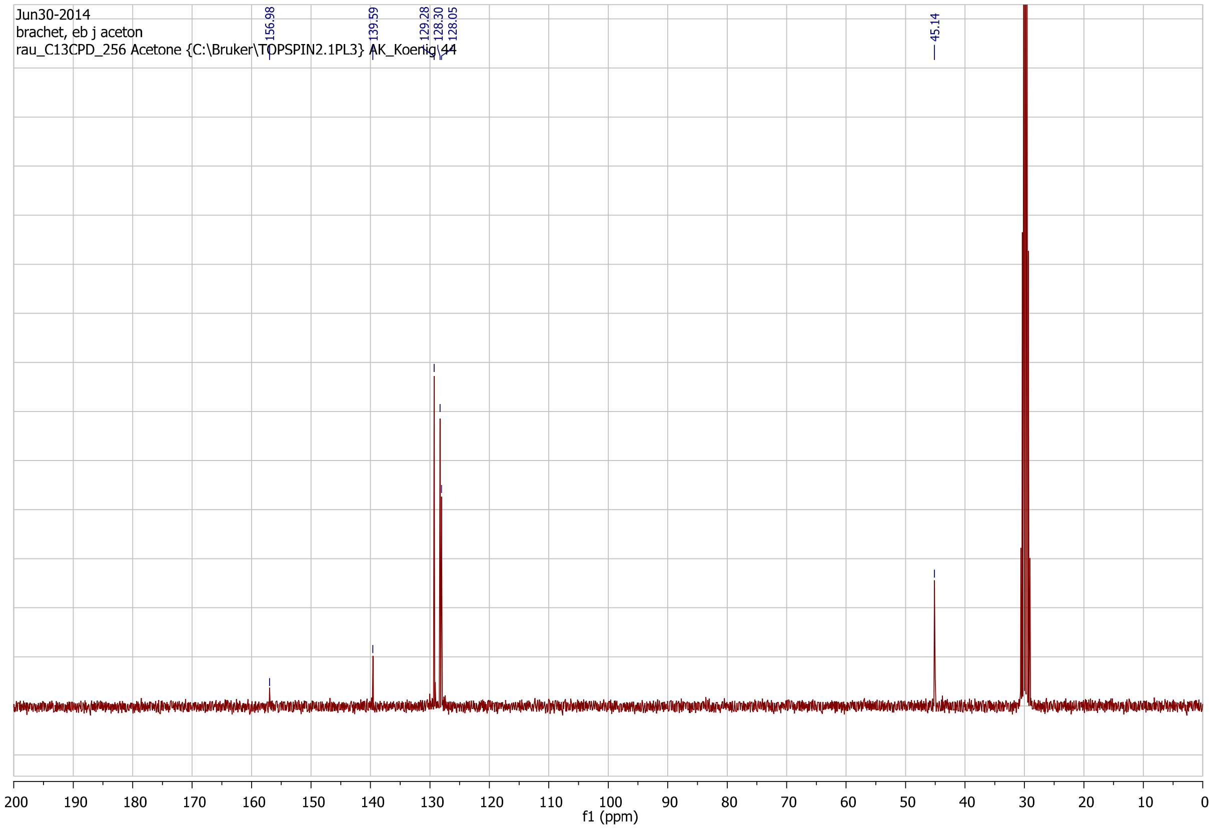

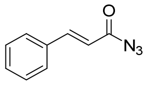

Compound 1j: cinnamoyl azide

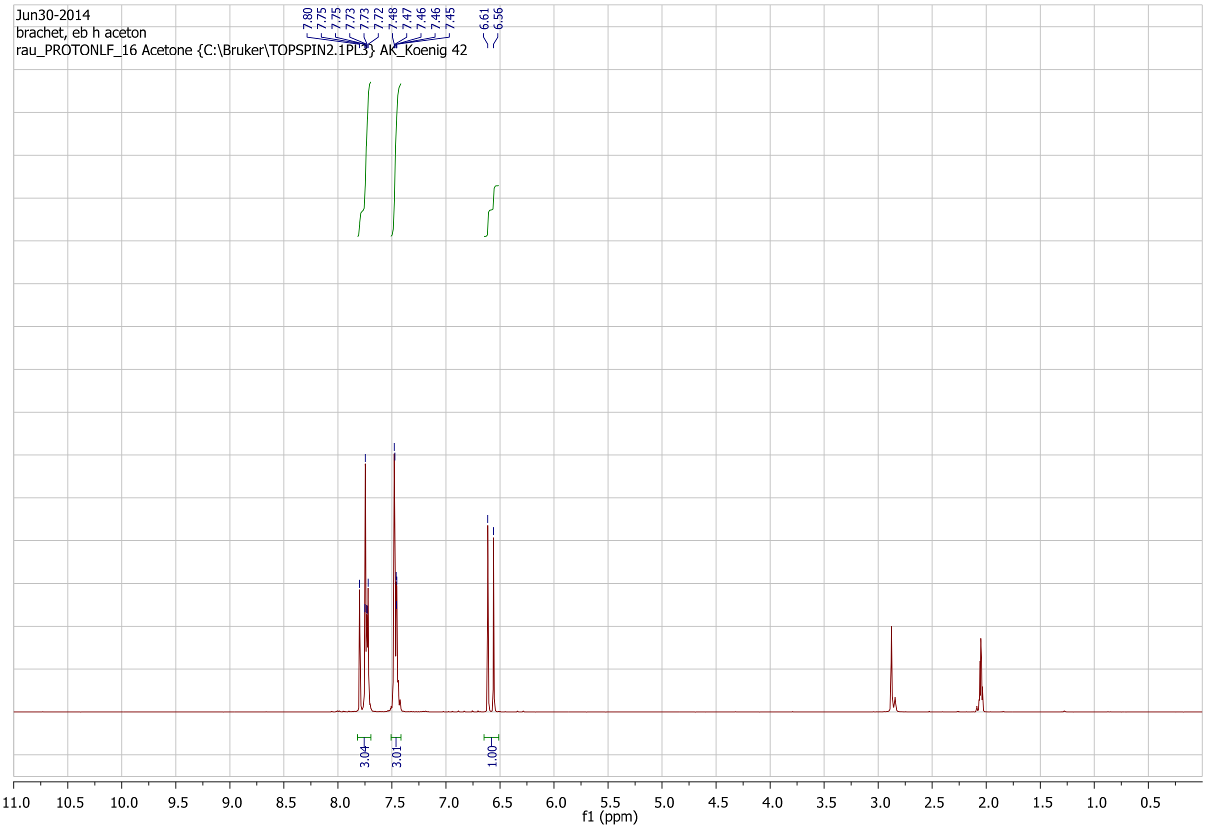

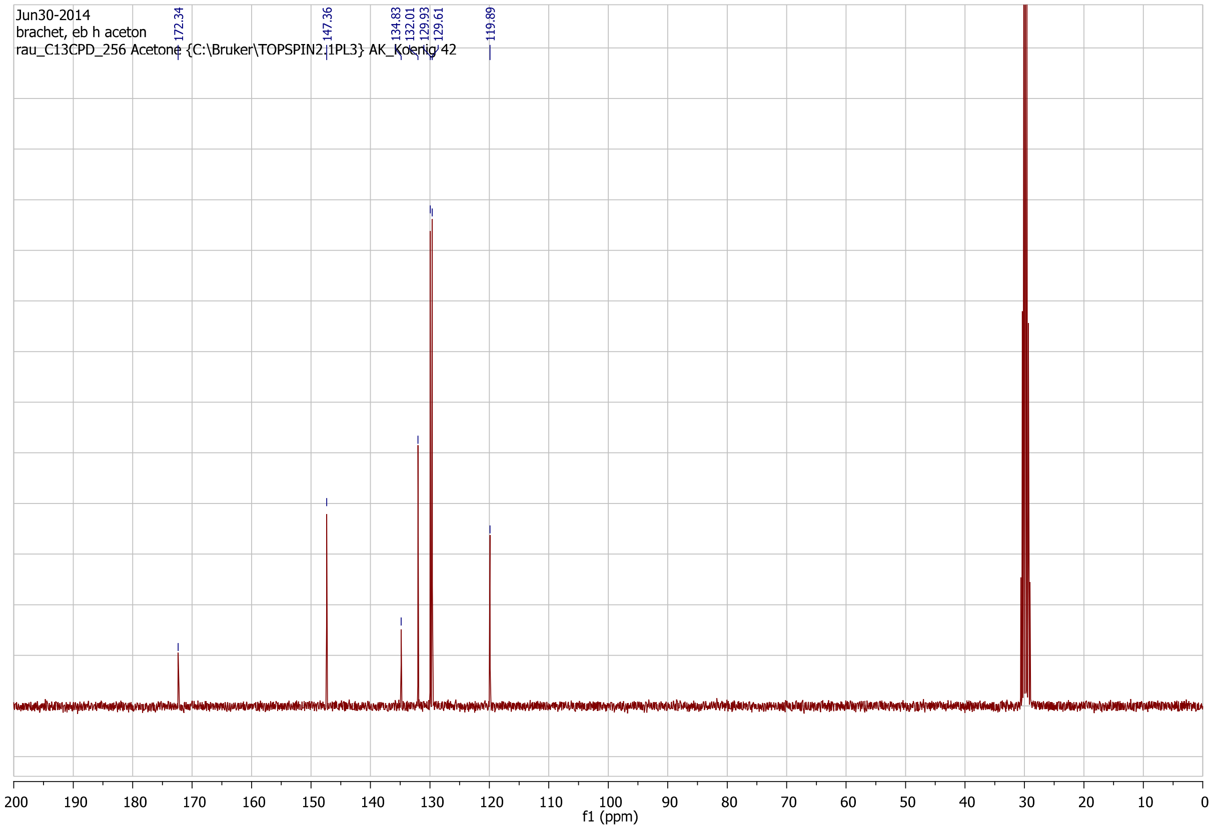

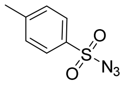

Compound 1k: 4-methylbenzenesulfonyl azide

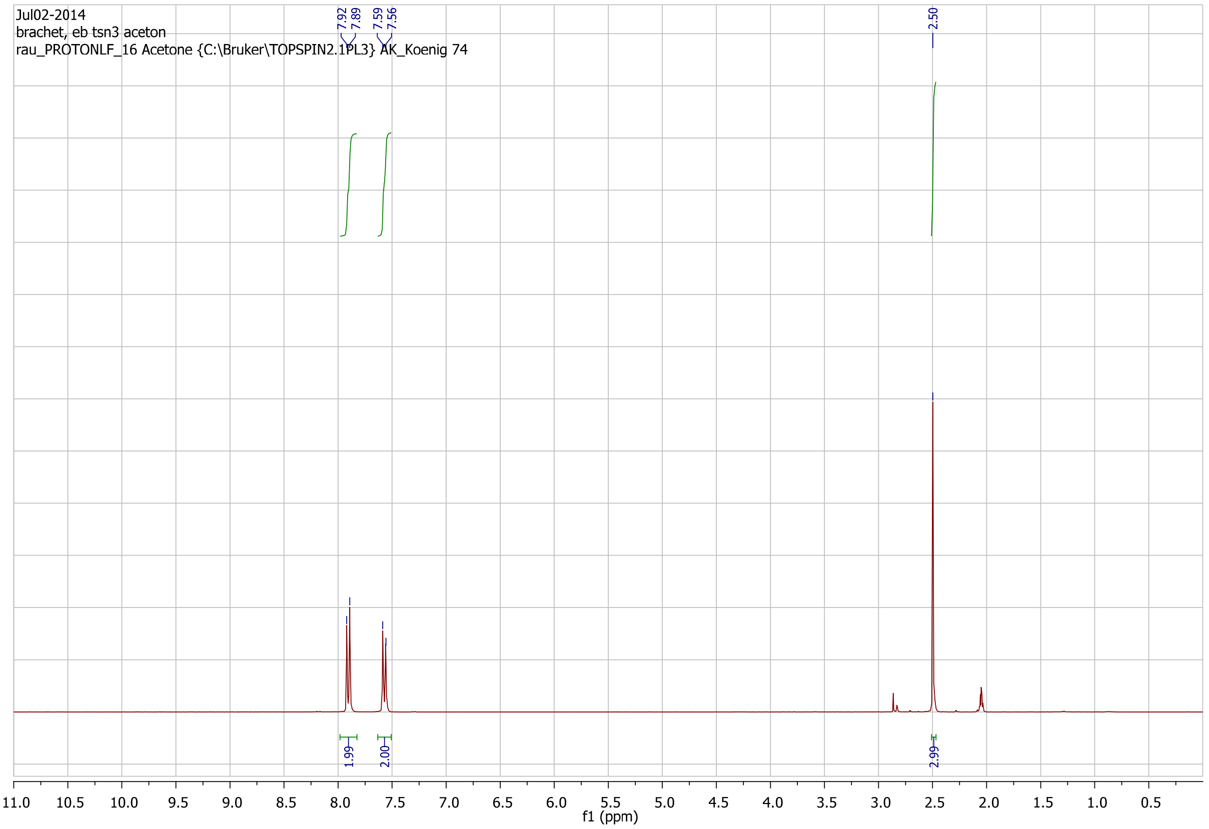

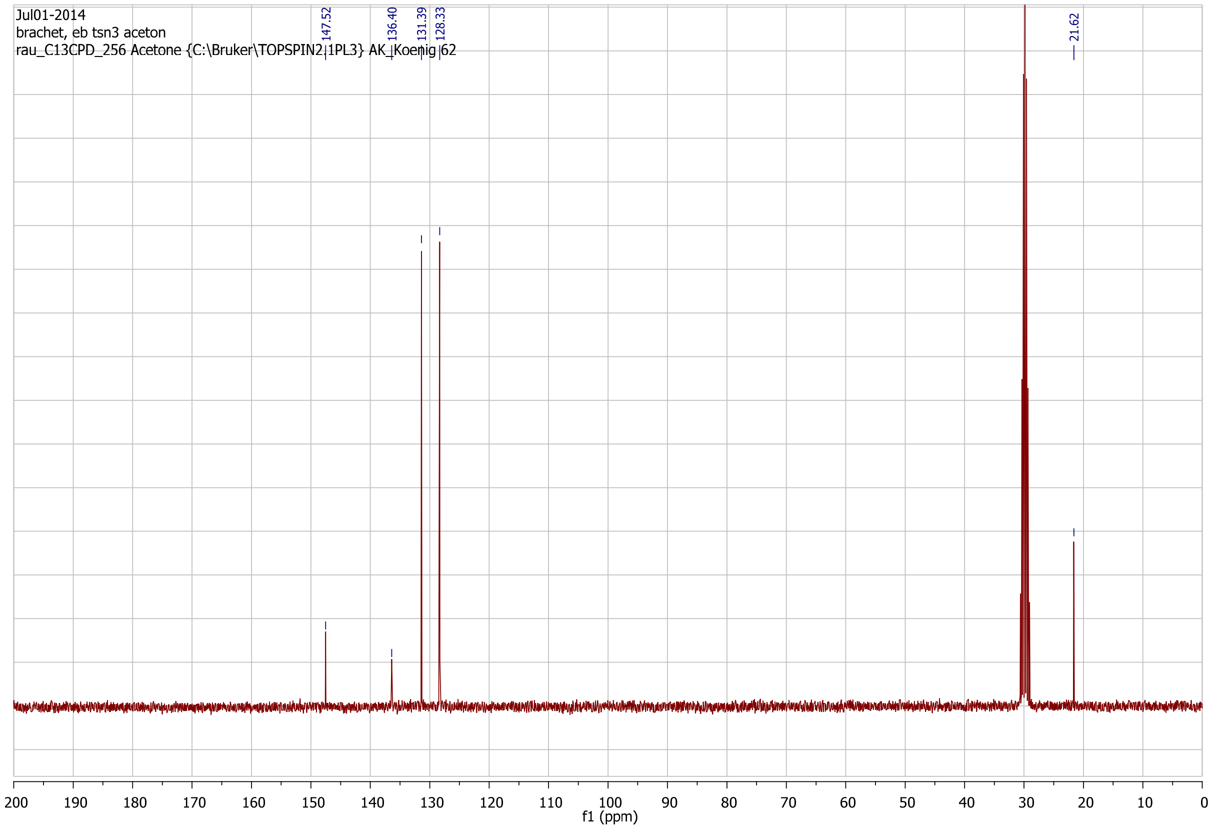

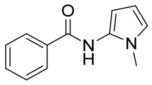

Compound 3a: N-(1-methyl-1H-pyrrol-2-yl)benzamide

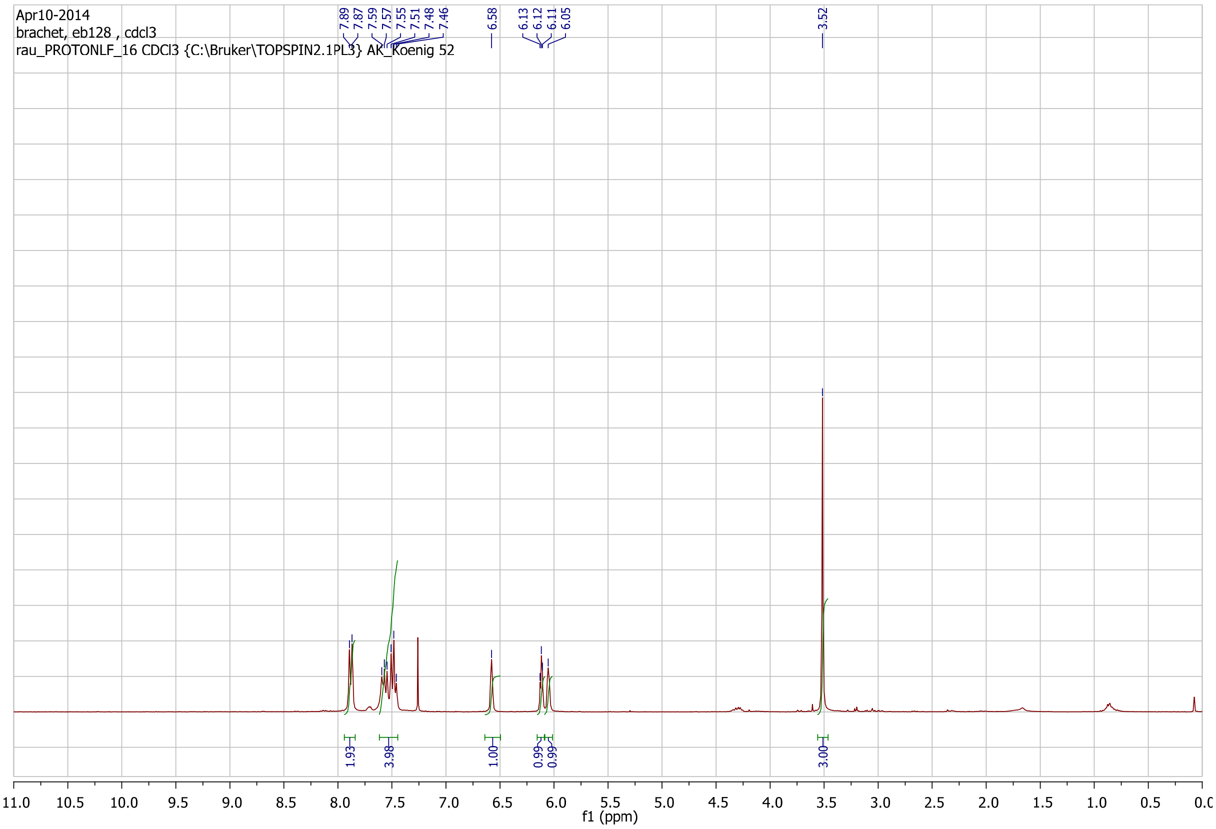

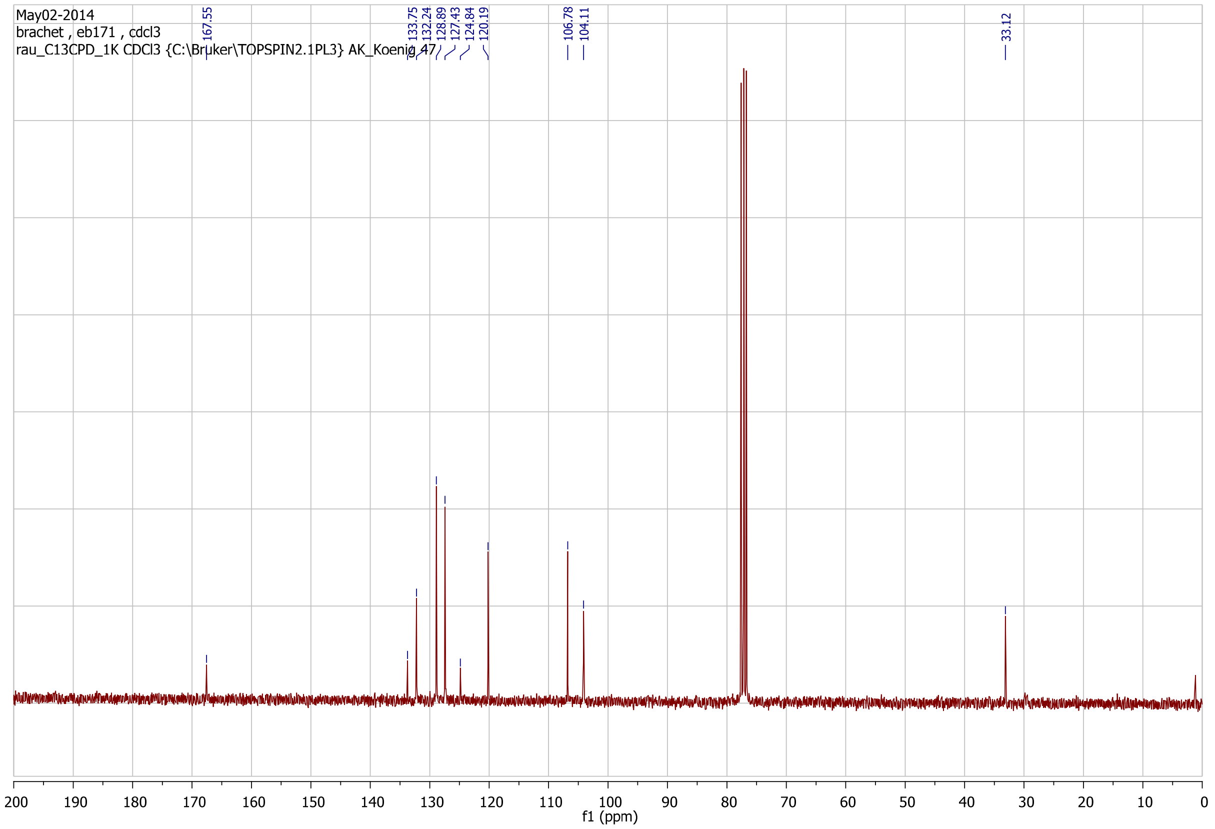

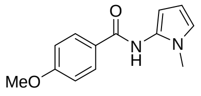

Compound 3b: 4-methoxy-N-(1-methyl-1H-pyrrol-2-yl)benzamide

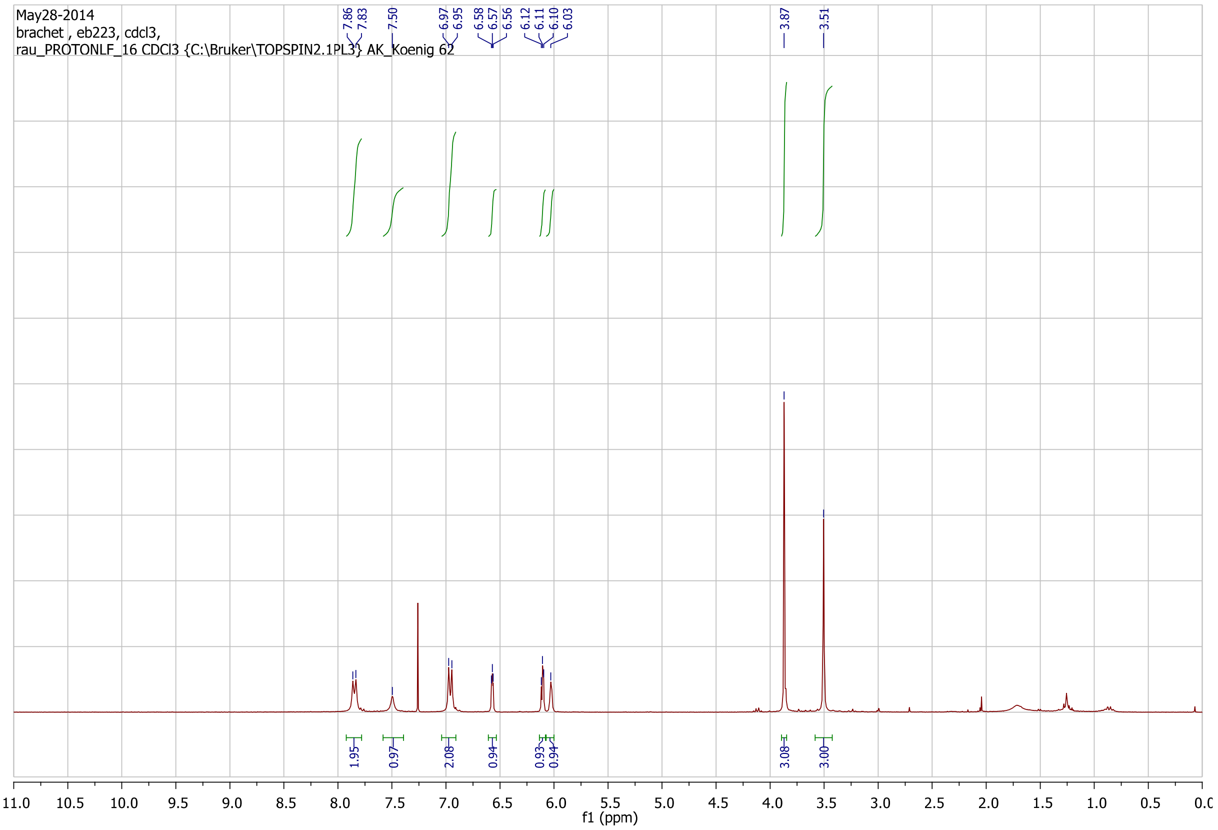

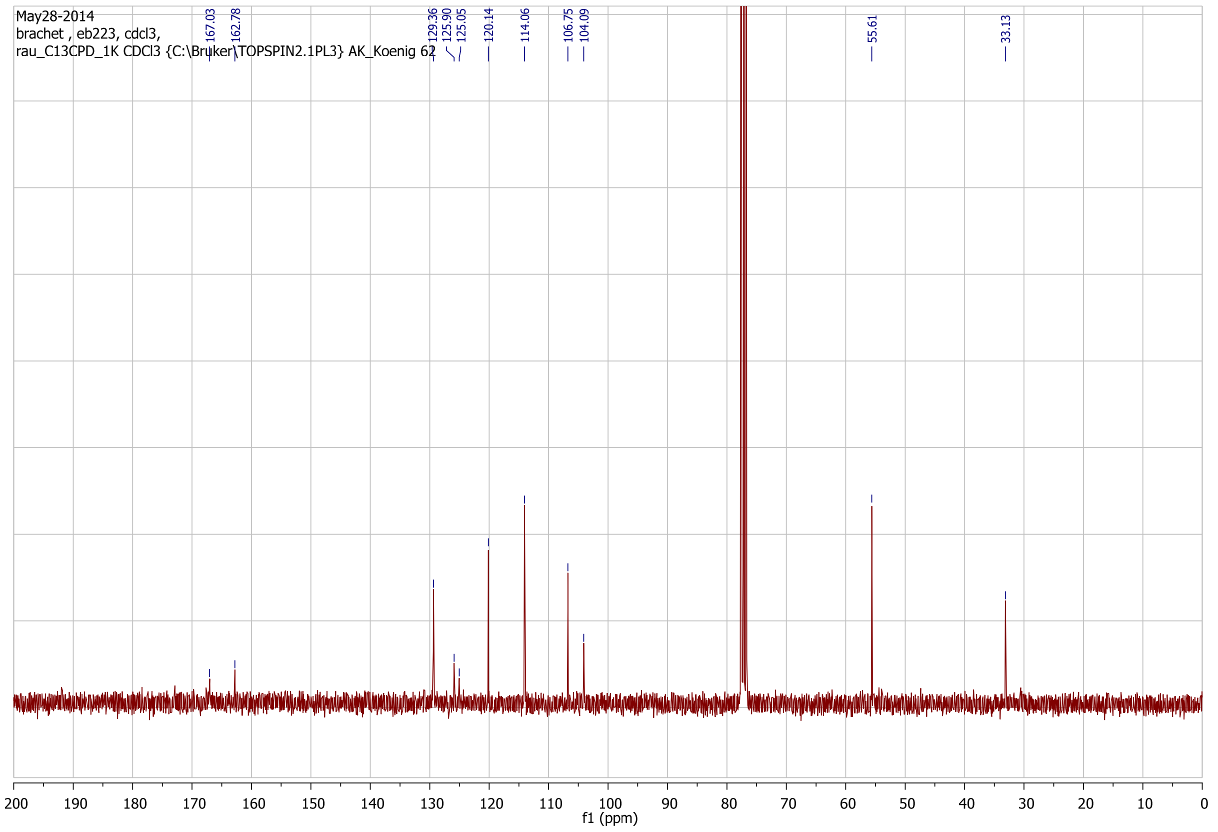

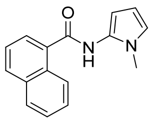

Compound 3c: N-(1-methyl-1H-pyrrol-2-yl)-1-naphthamide

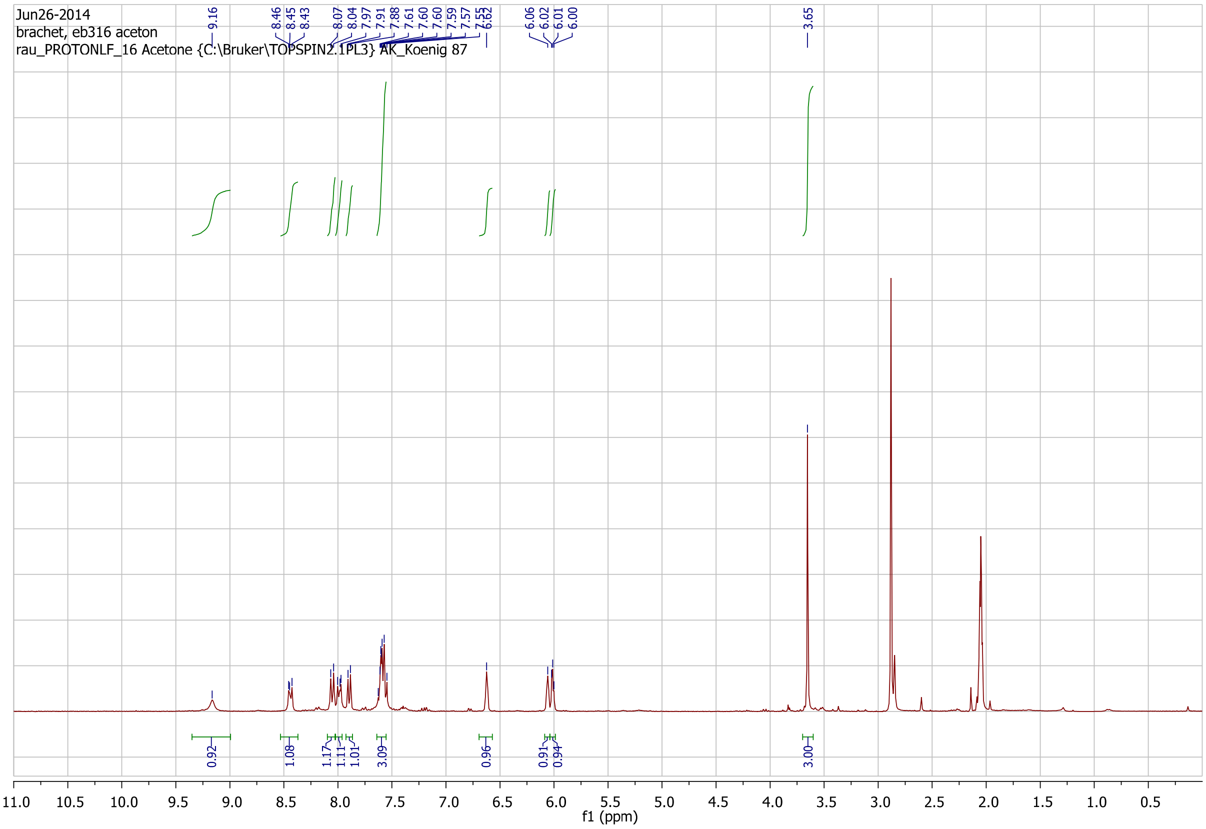

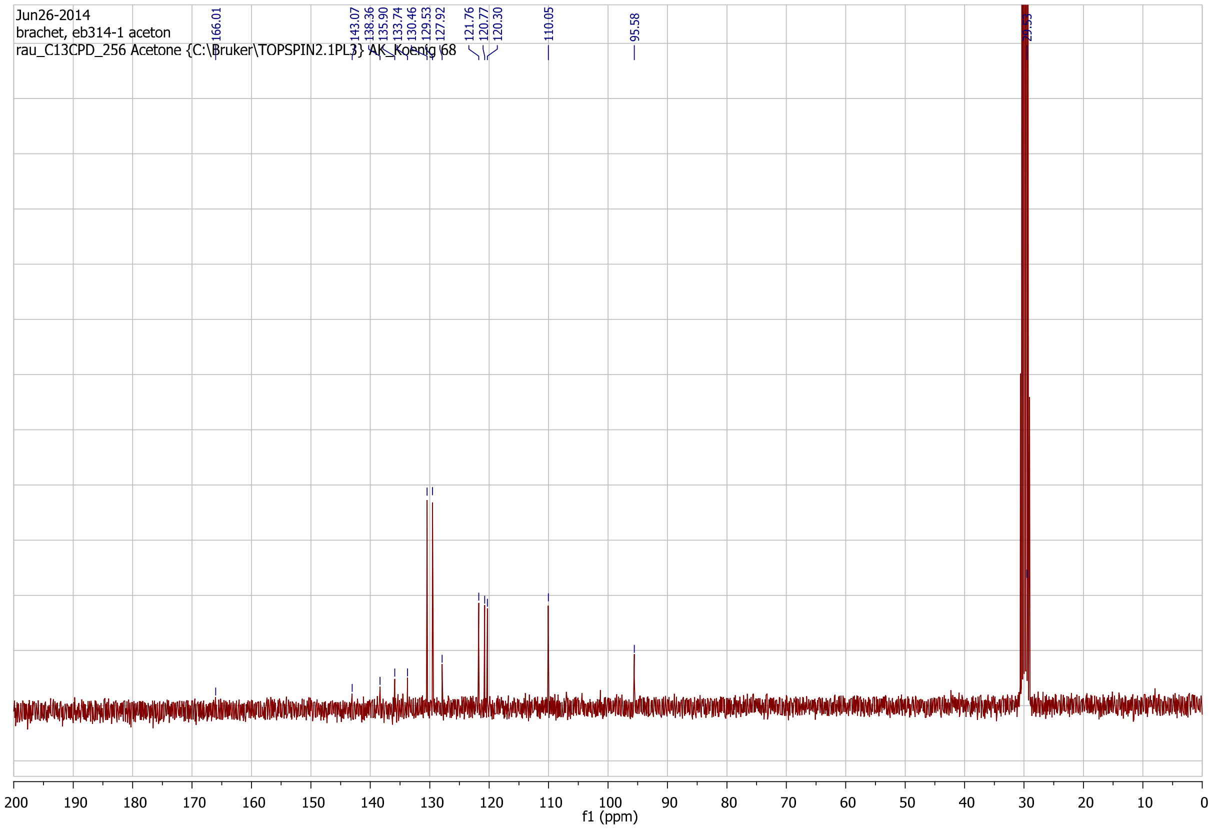

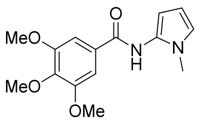

Compound 3d: 3,4,5-trimethoxy-N-(1-methyl-1H-pyrrol-2-yl)benzamide

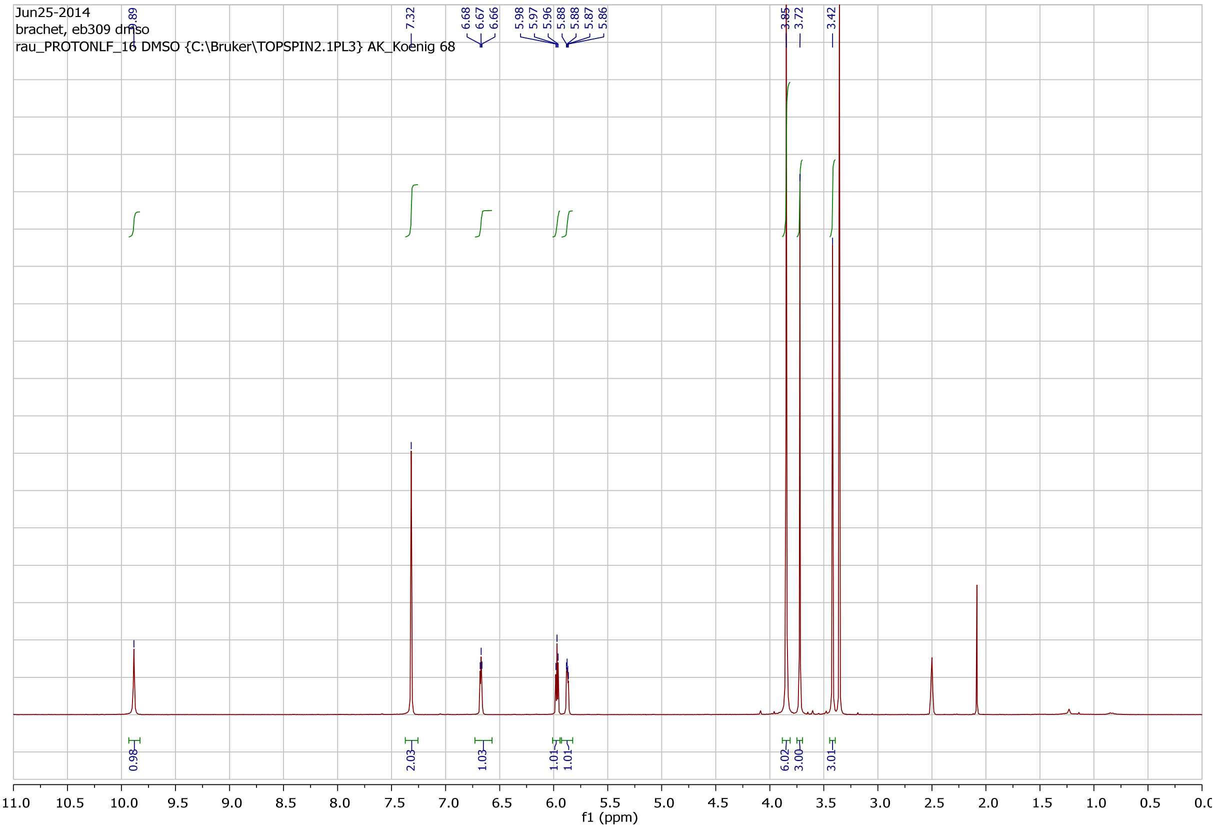

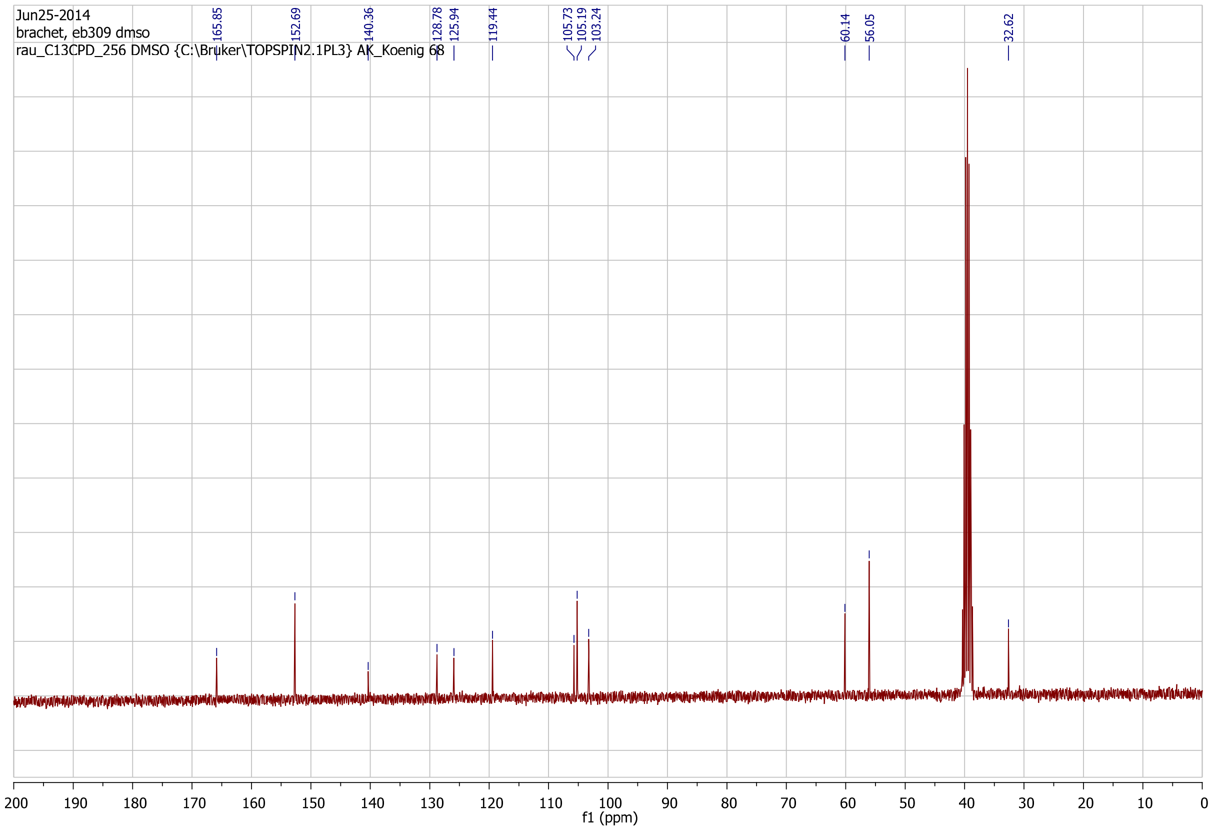

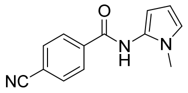

Compound 3e: 4-cyano-N-(1-methyl-1H-pyrrol-2-yl)benzamide

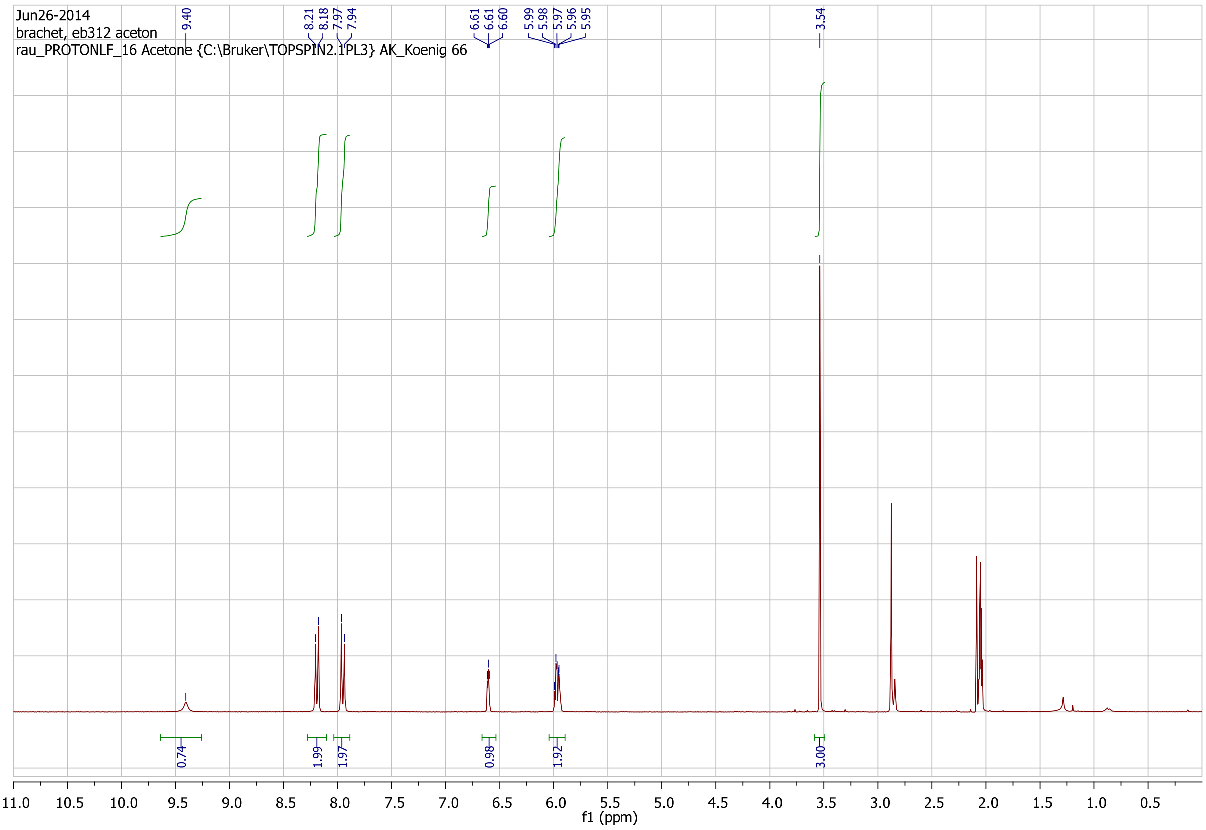

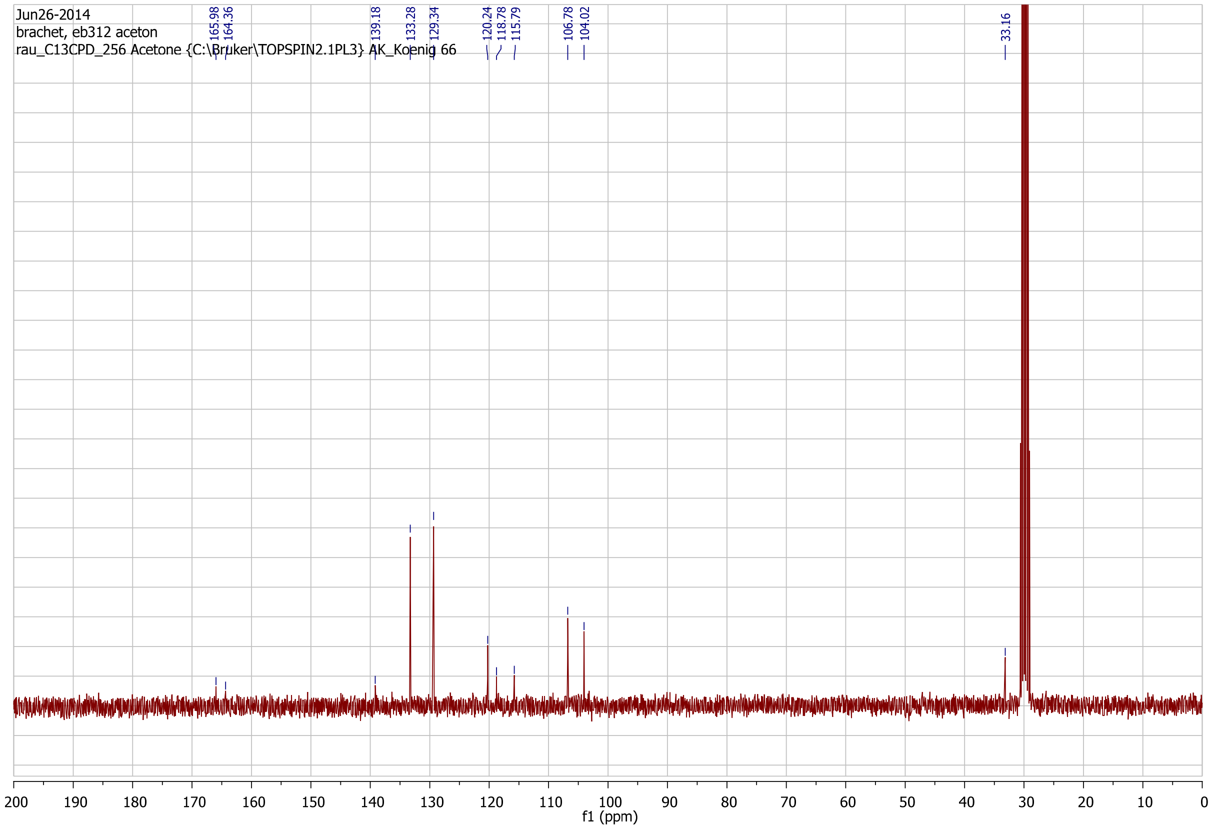

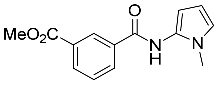

Compound 3f: methyl 3-((1-methyl-1H-pyrrol-2-yl)carbamoyl)benzoate

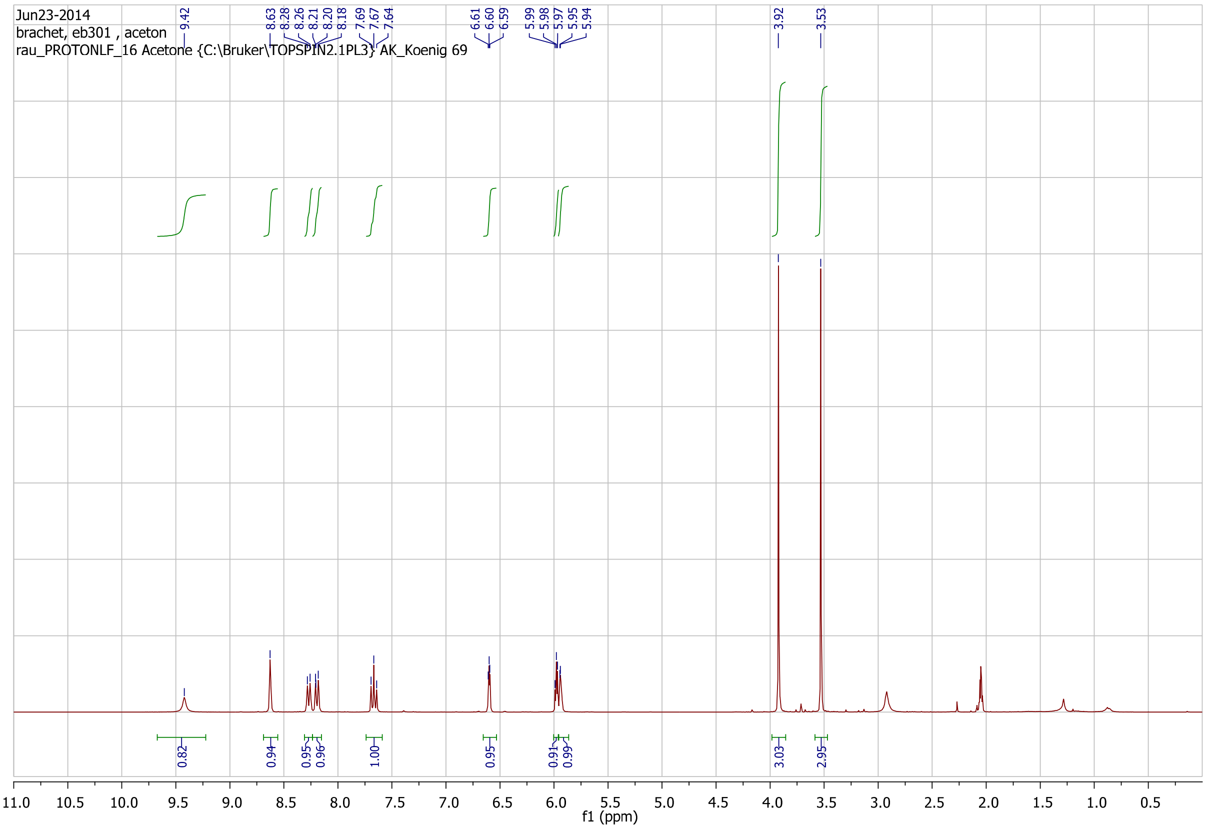

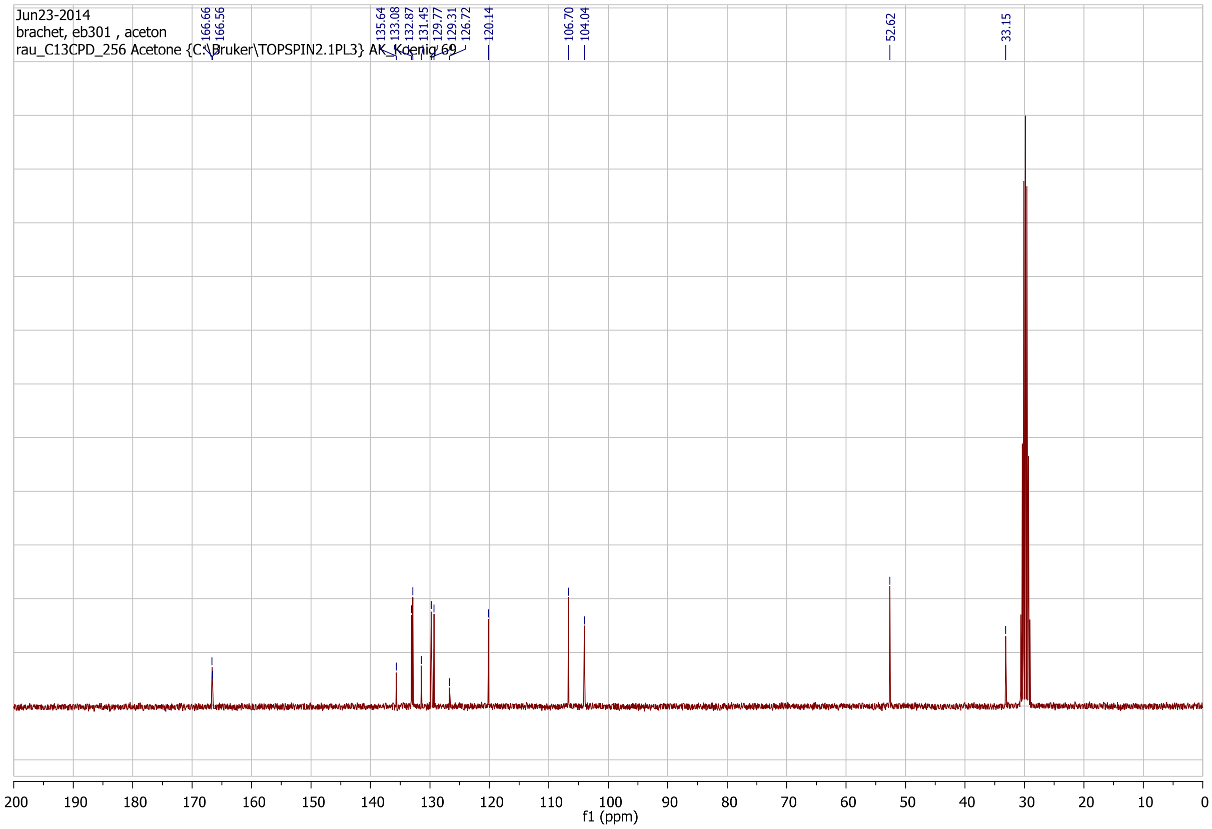

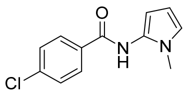

Compound 3g: 4-chloro-N-(1-methyl-1H-pyrrol-2-yl)benzamide

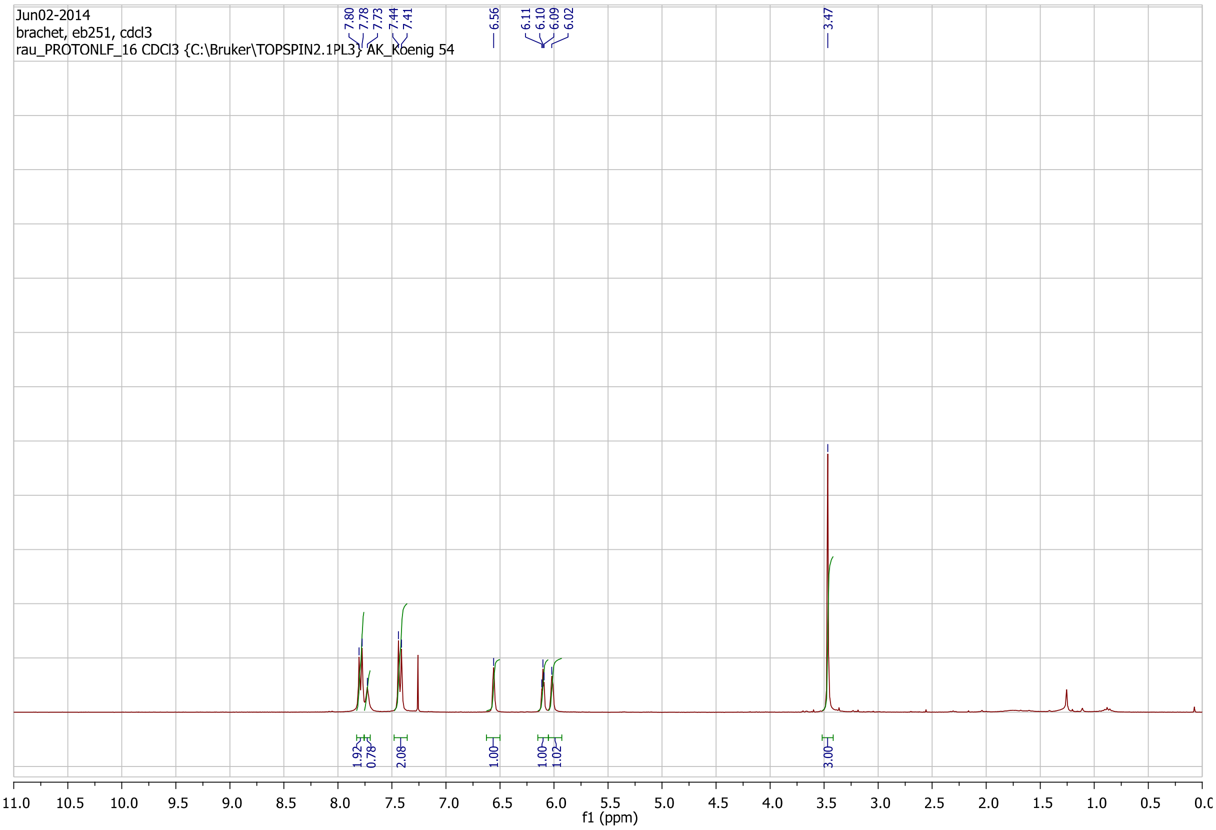

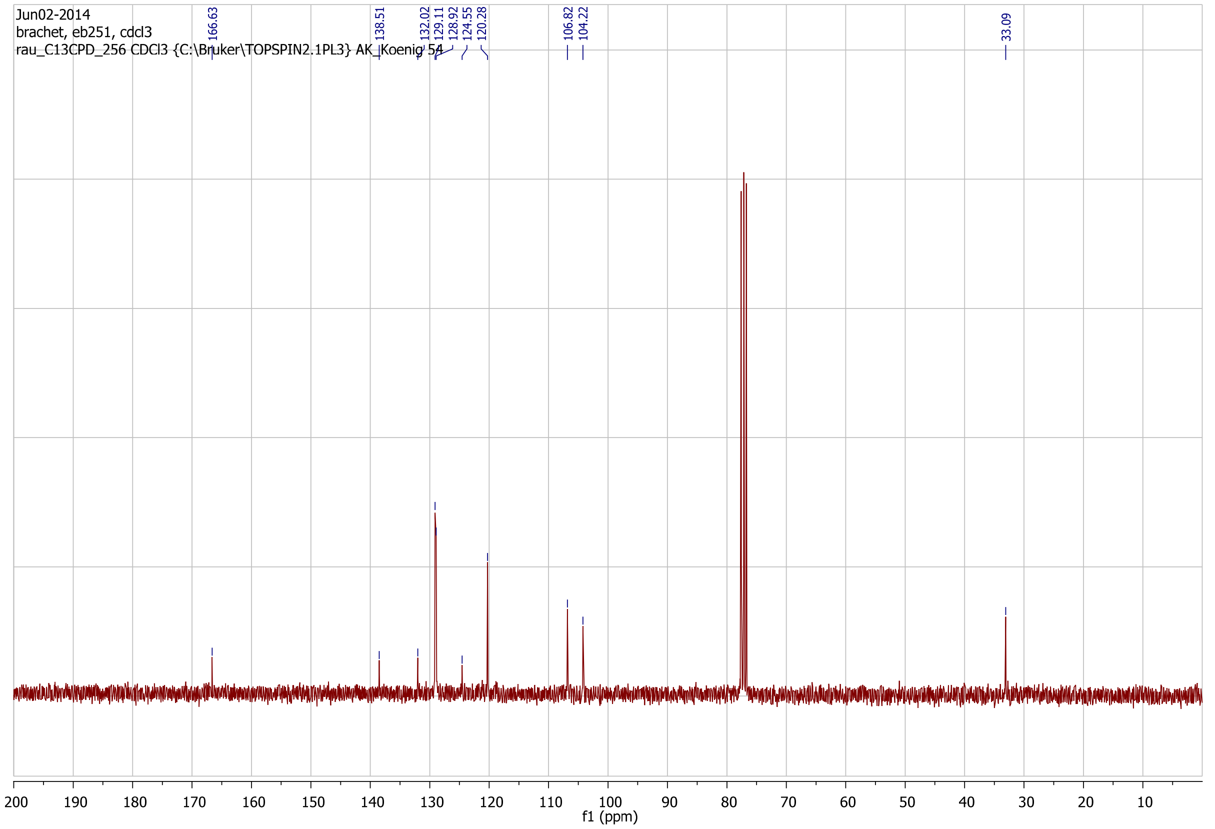

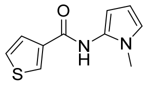

Compound 3h: N-(1-methyl-1H-pyrrol-2-yl)thiophene-3-carboxamide

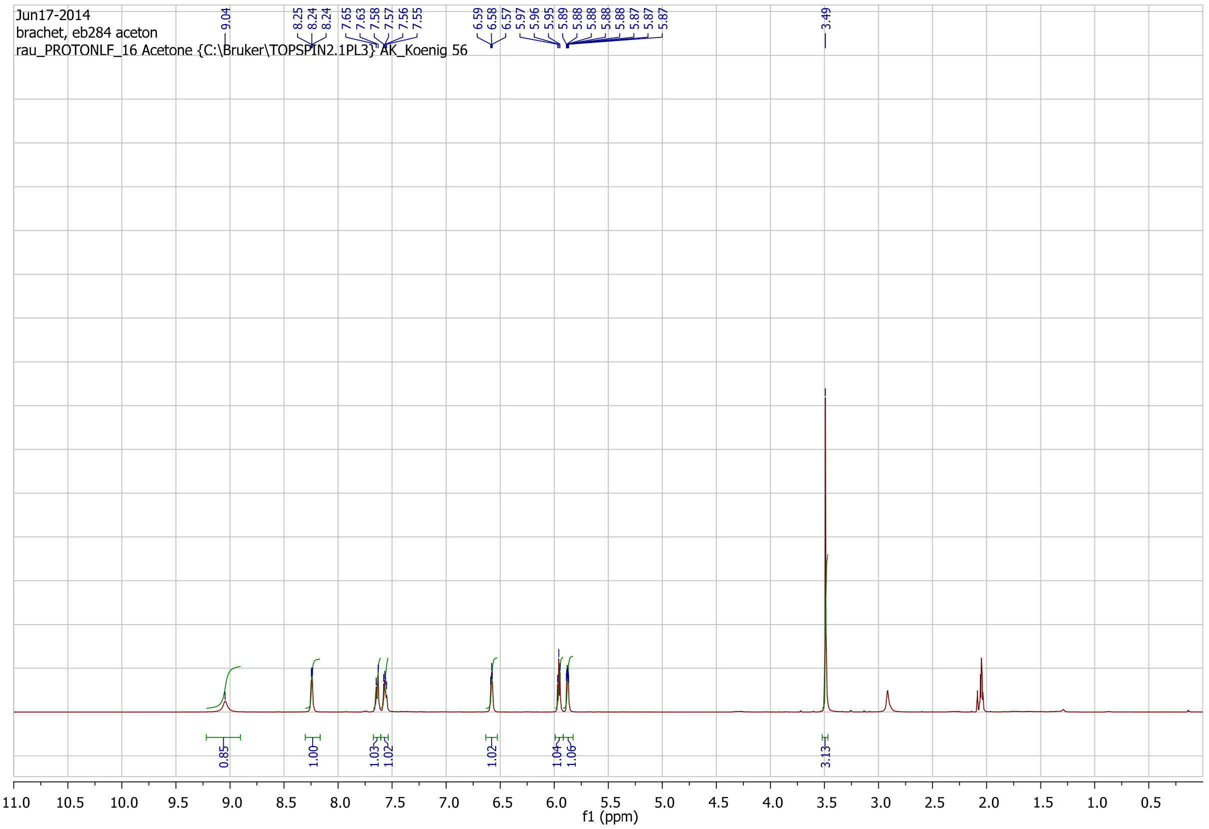

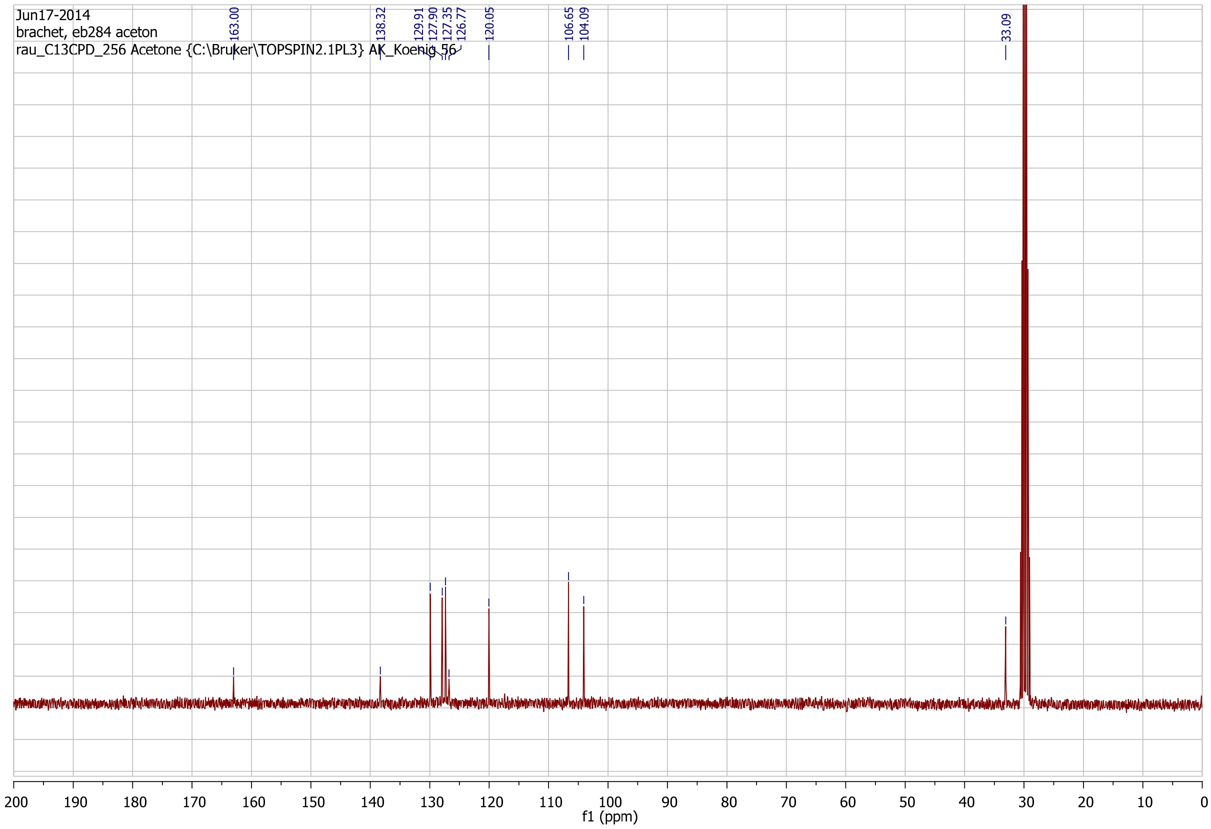

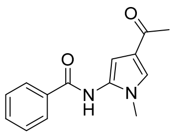

Compound 3j: N-(4-acetyl-1-methyl-1H-pyrrol-2-yl)benzamide

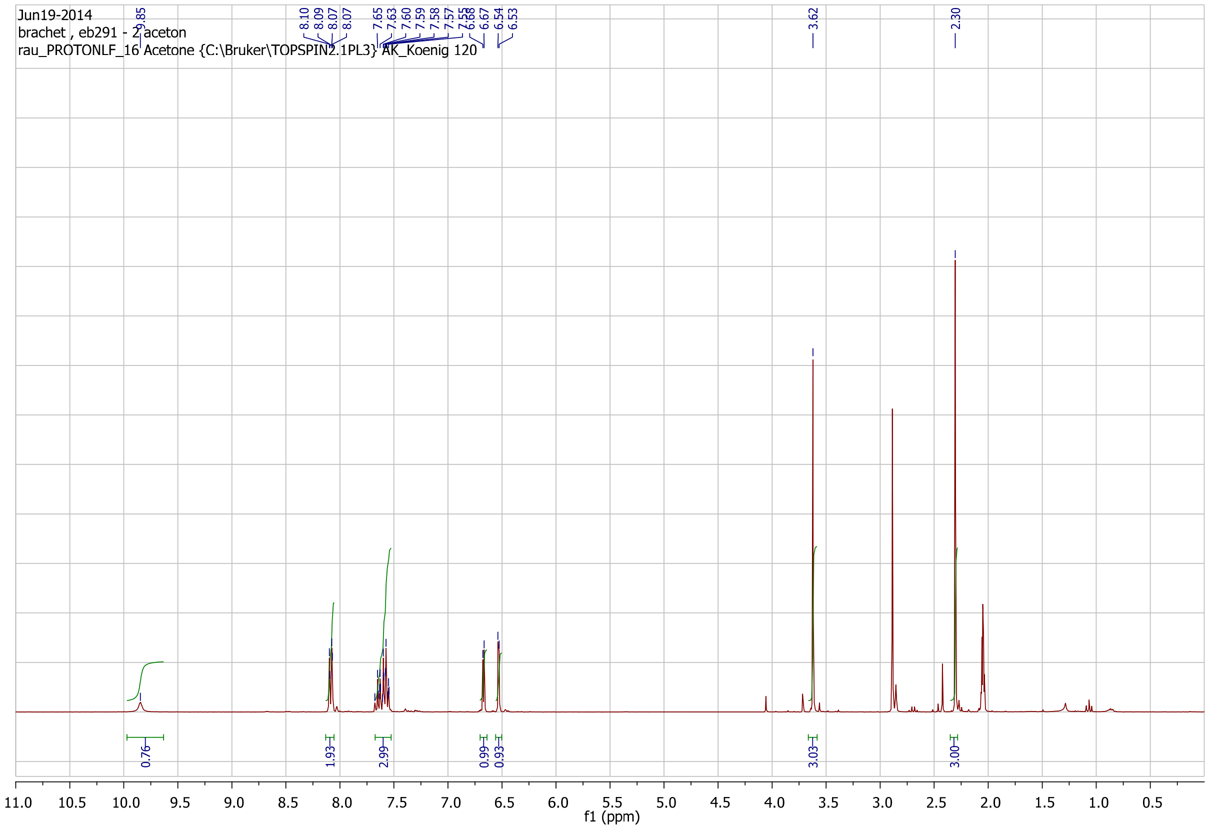

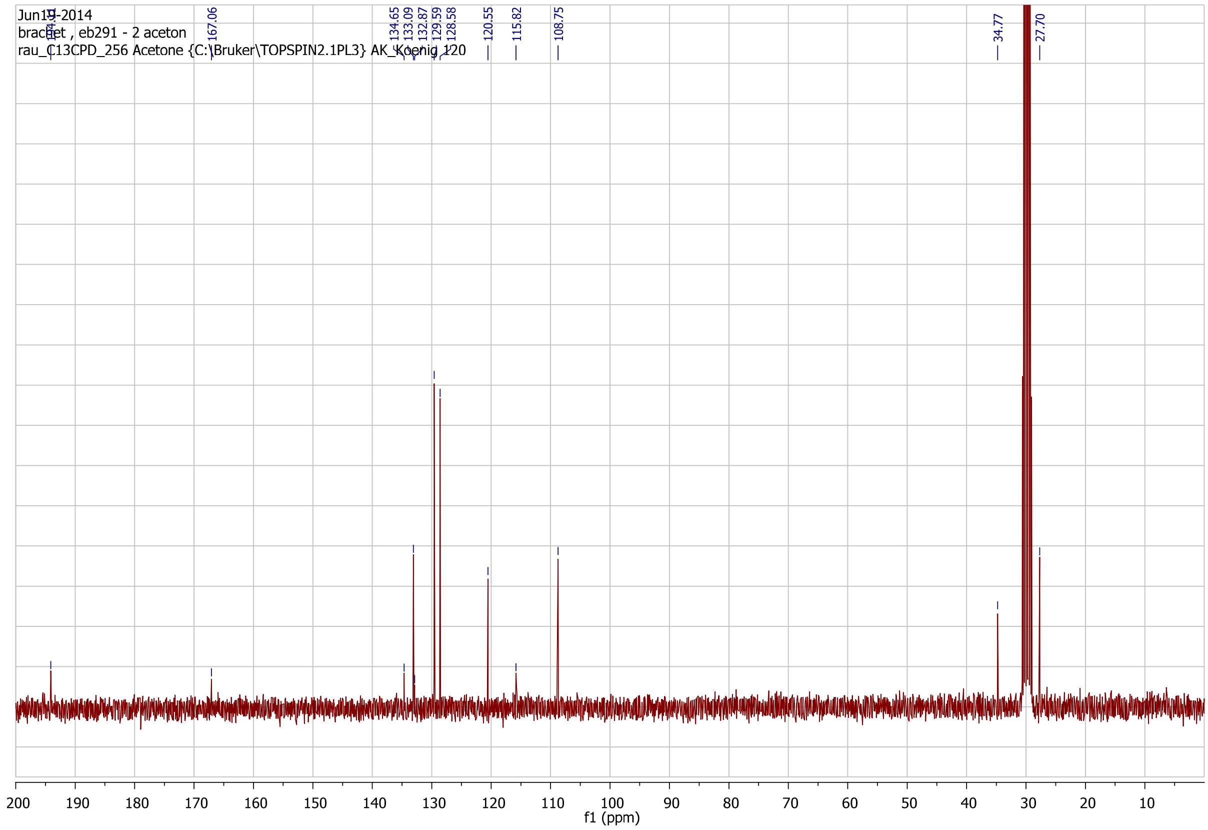

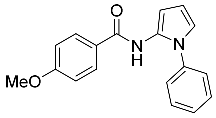

Compound 3k: 4-methoxy-N-(1-phenyl-1H-pyrrol-2-yl)benzamide

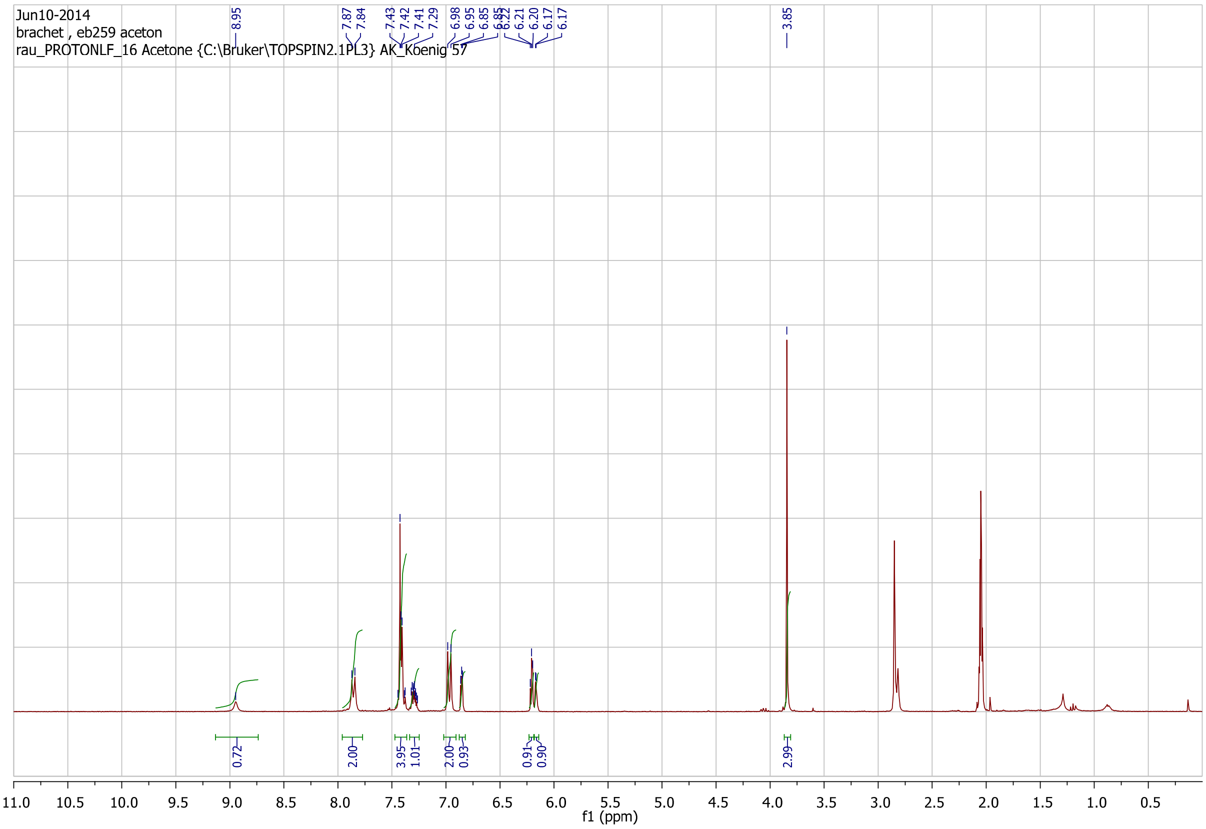

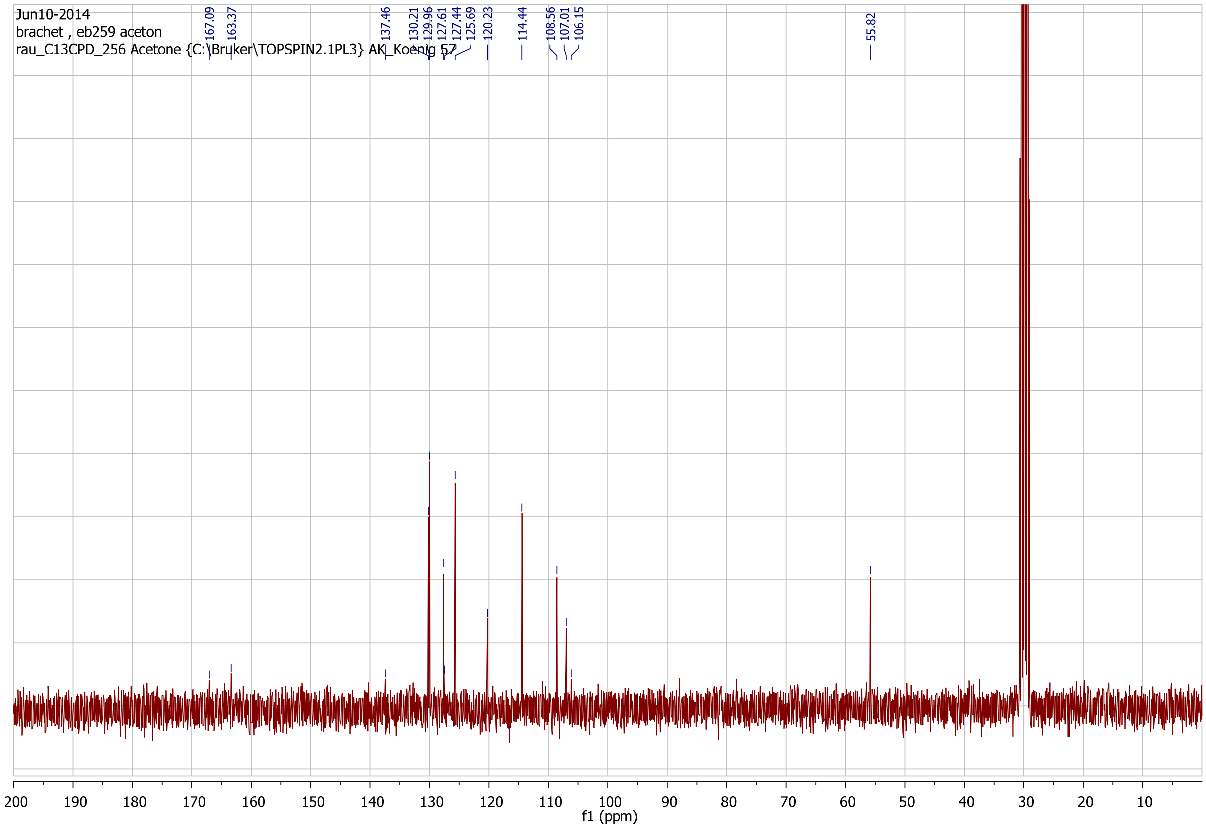

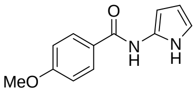

Compound 3l: 4-methoxy-N-(1H-pyrrol-2-yl)benzamide

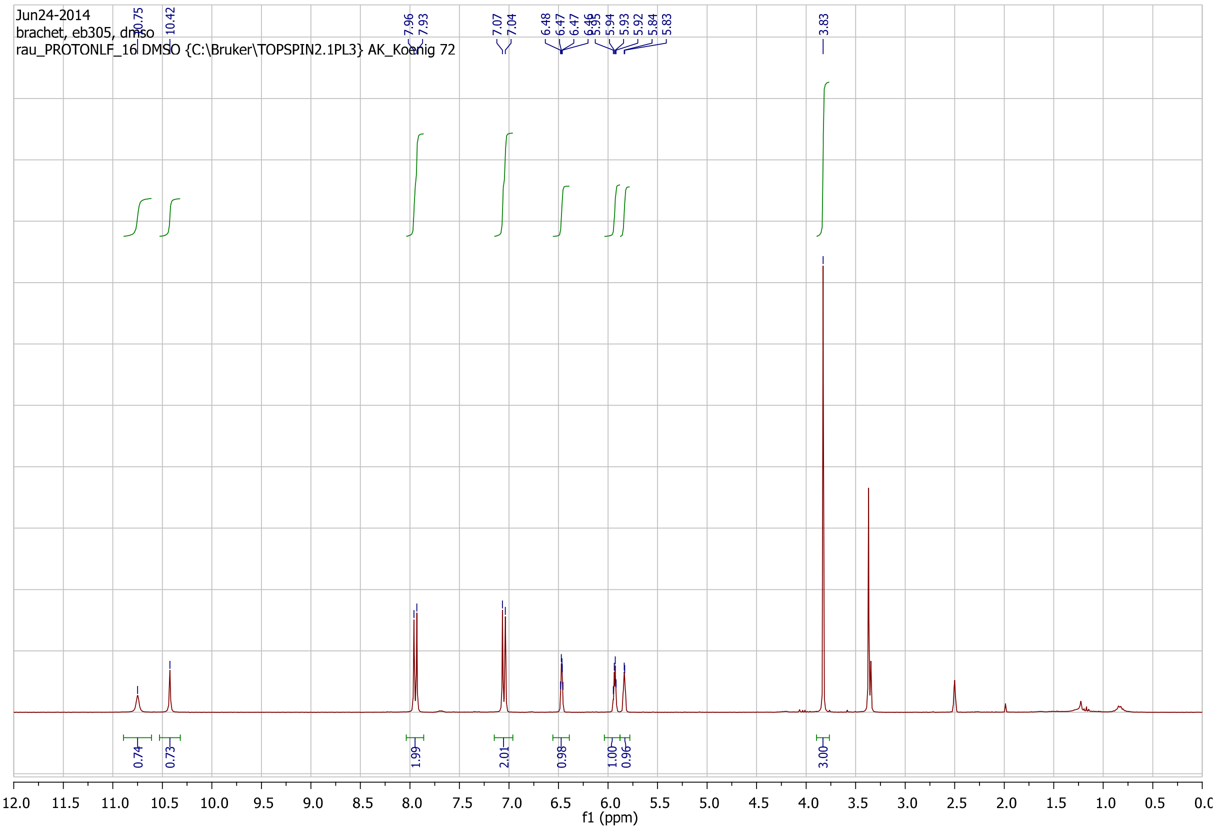

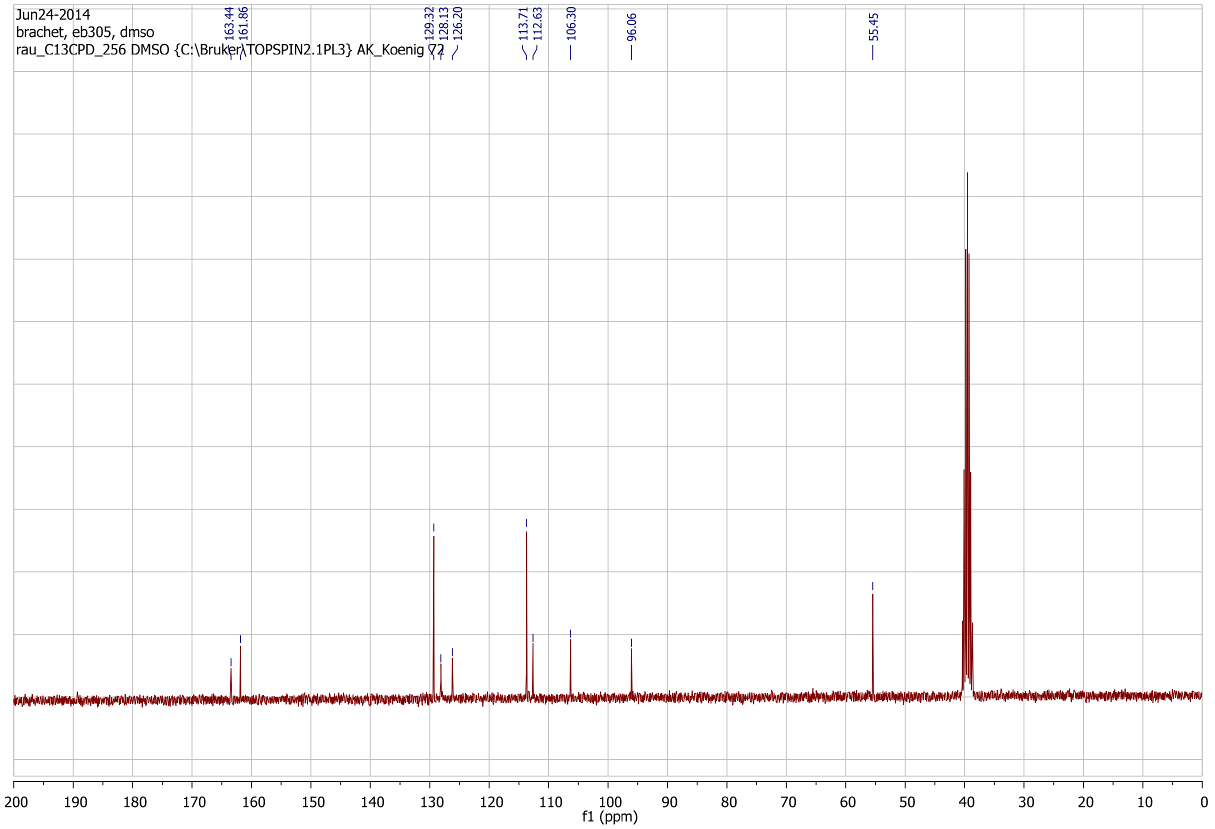

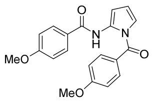

Compound 3m: 4-methoxy-N-(1-(4-methoxybenzoyl)-1H-pyrrol-2-yl)benzamide

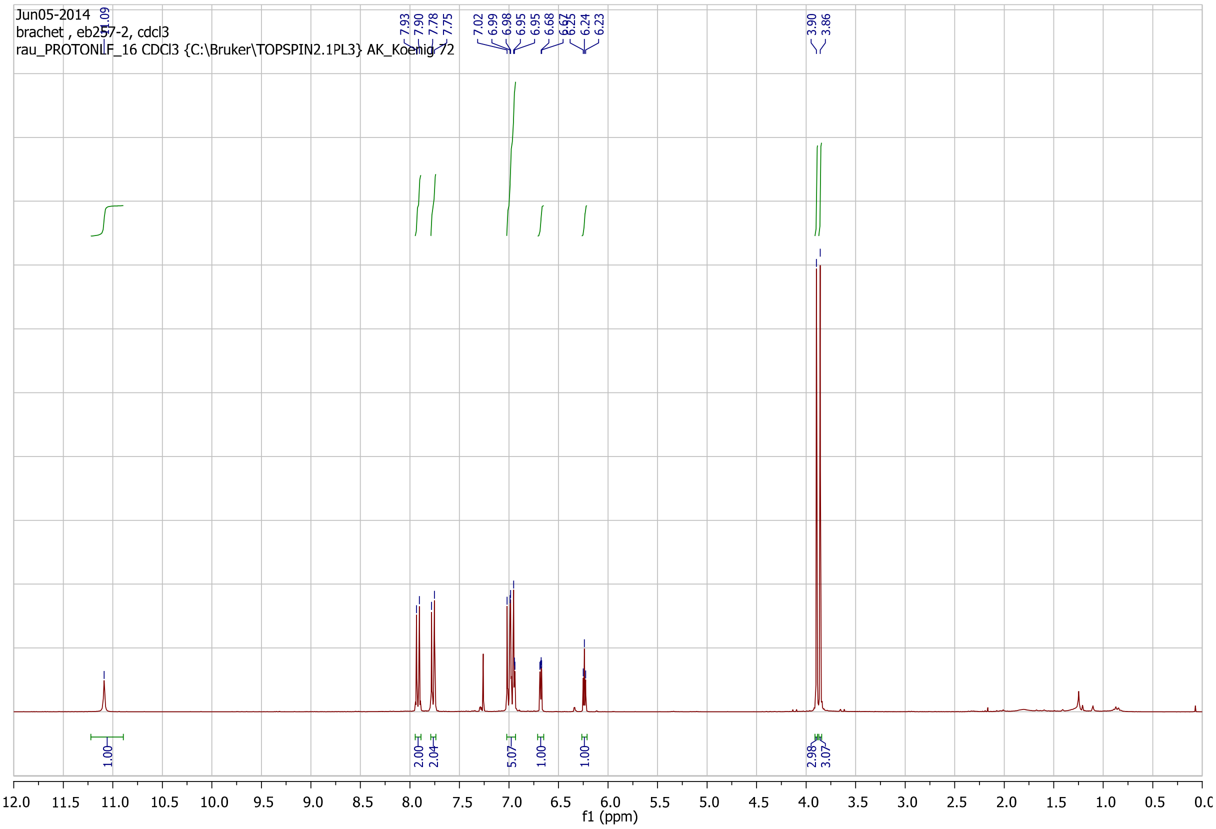

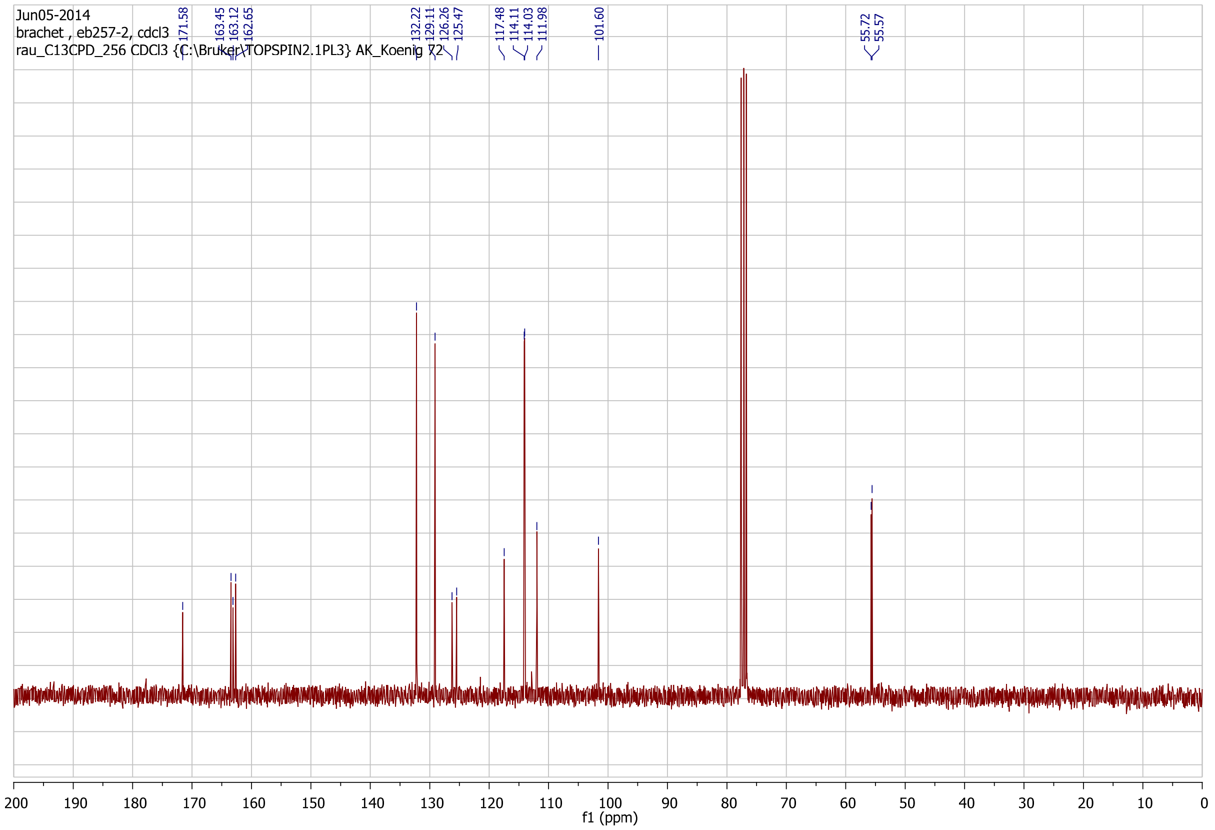

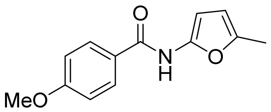

Compound 3n: 4-methoxy-N-(5-methylfuran-2-yl)benzamide

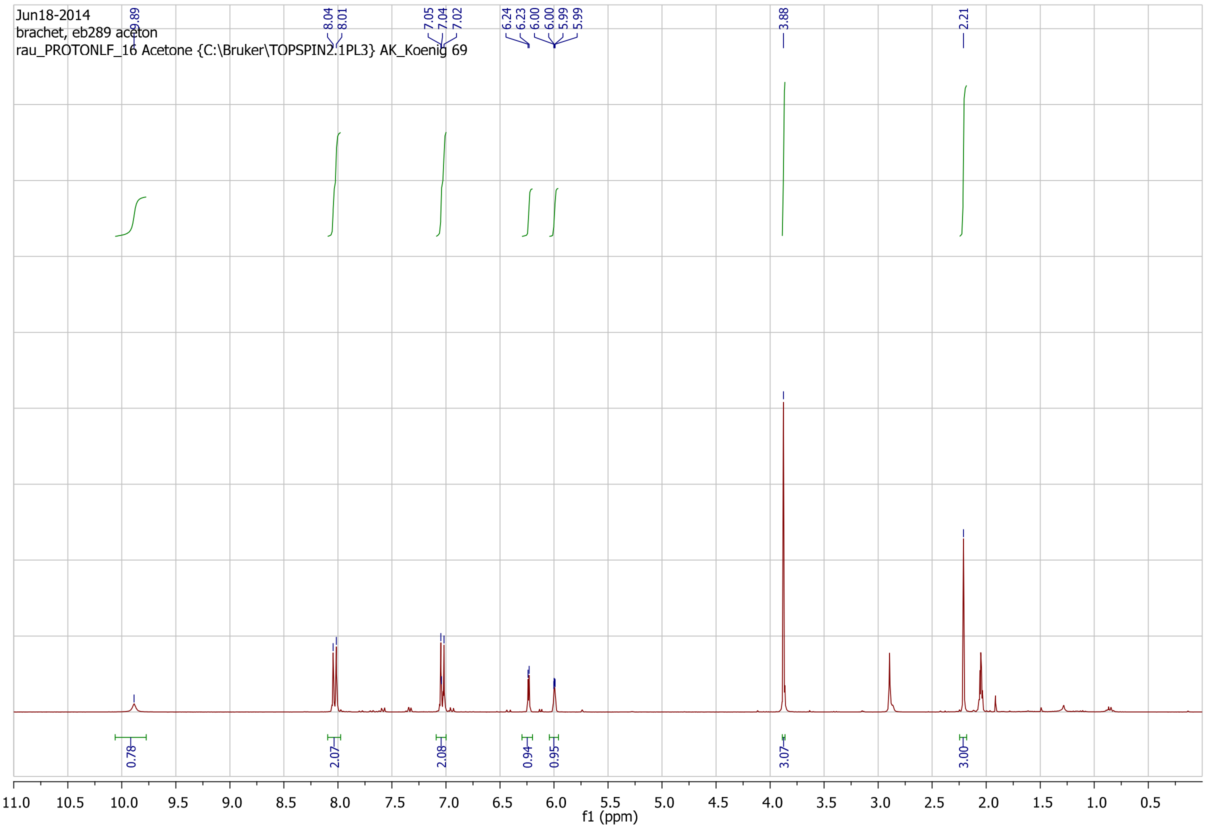

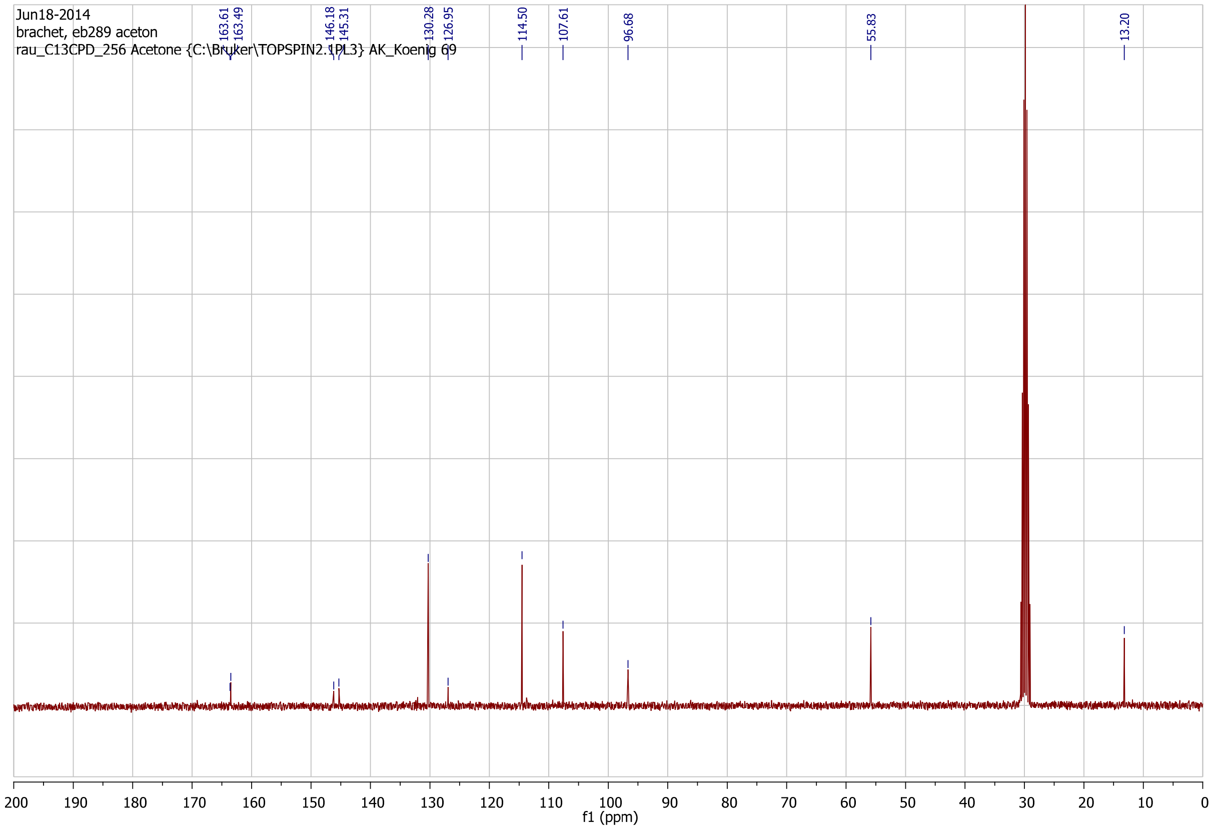

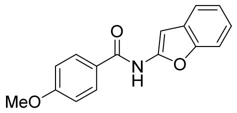

Compound 3o: N-(benzofuran-2-yl)-4-methoxybenzamide

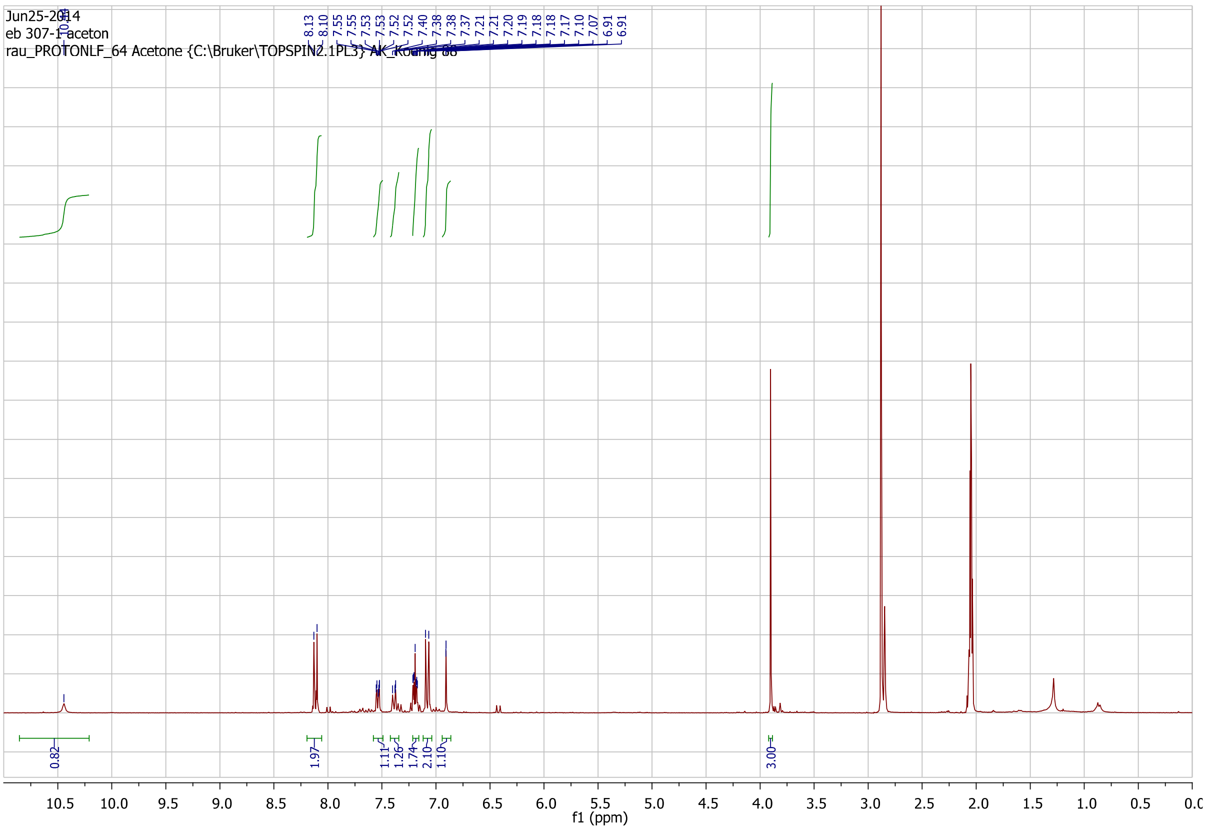

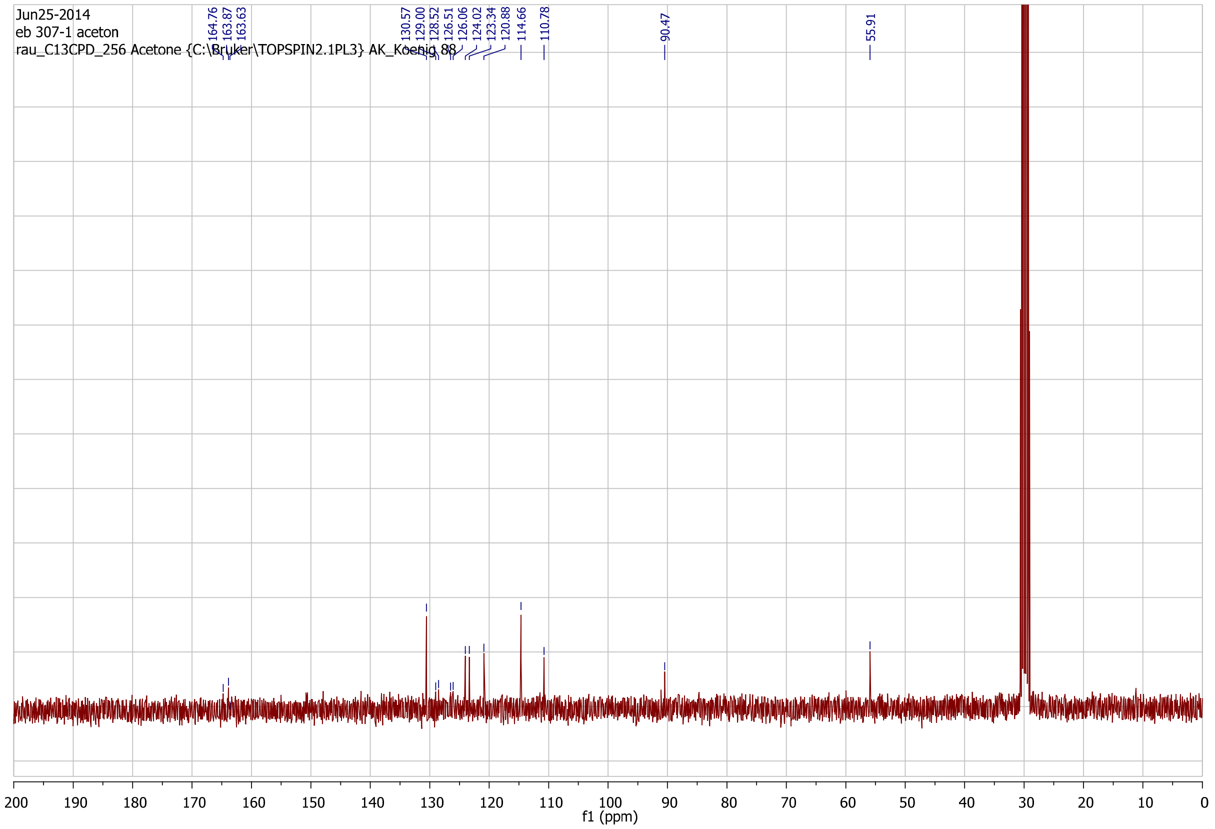

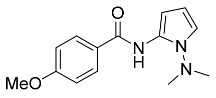

Compound 3p: N-(1-(dimethylamino)-1H-pyrrol-2-yl)-4-methoxybenzamide

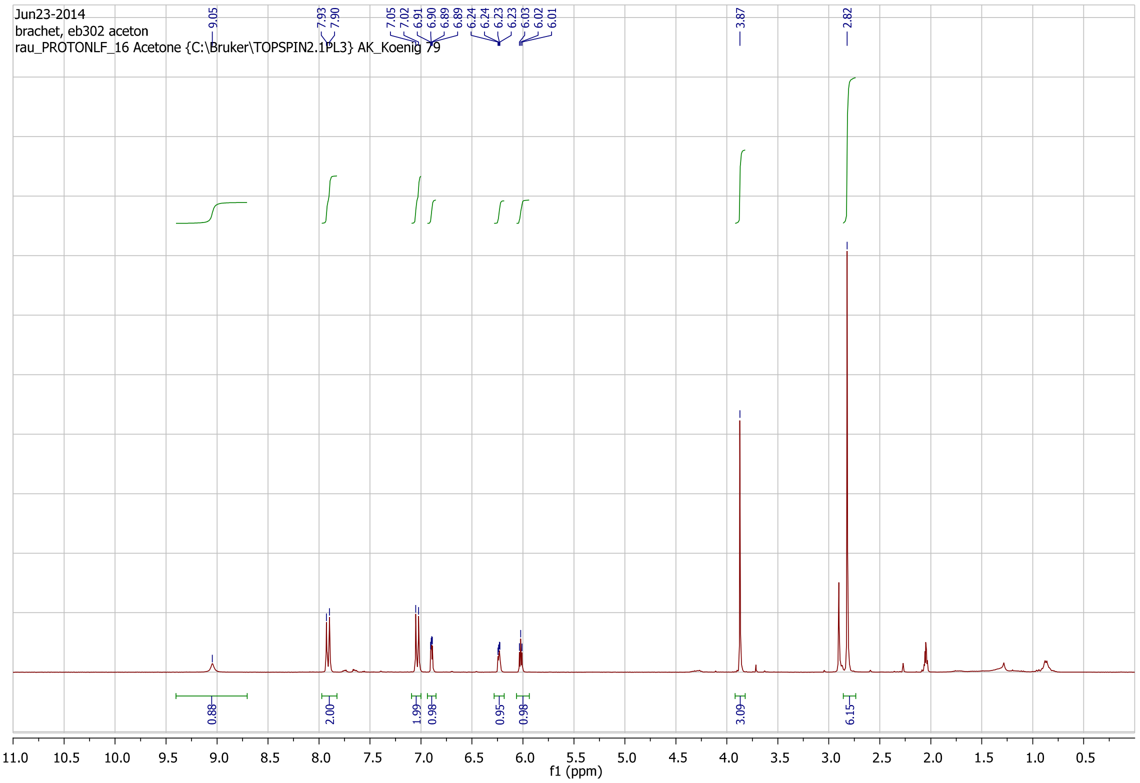

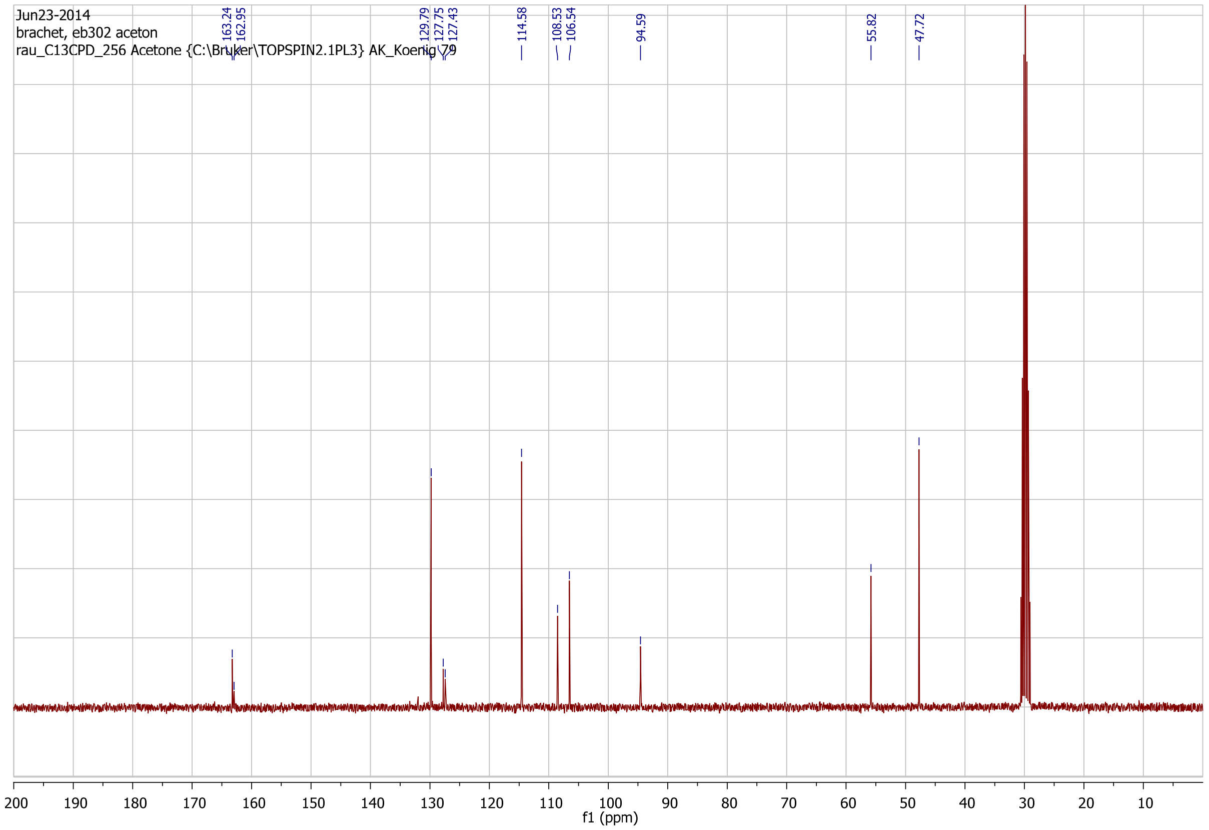

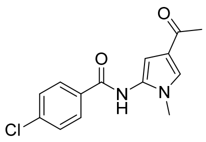

Compound 3q: N-(4-acetyl-1-methyl-1H-pyrrol-2-yl)-4-chlorobenzamide

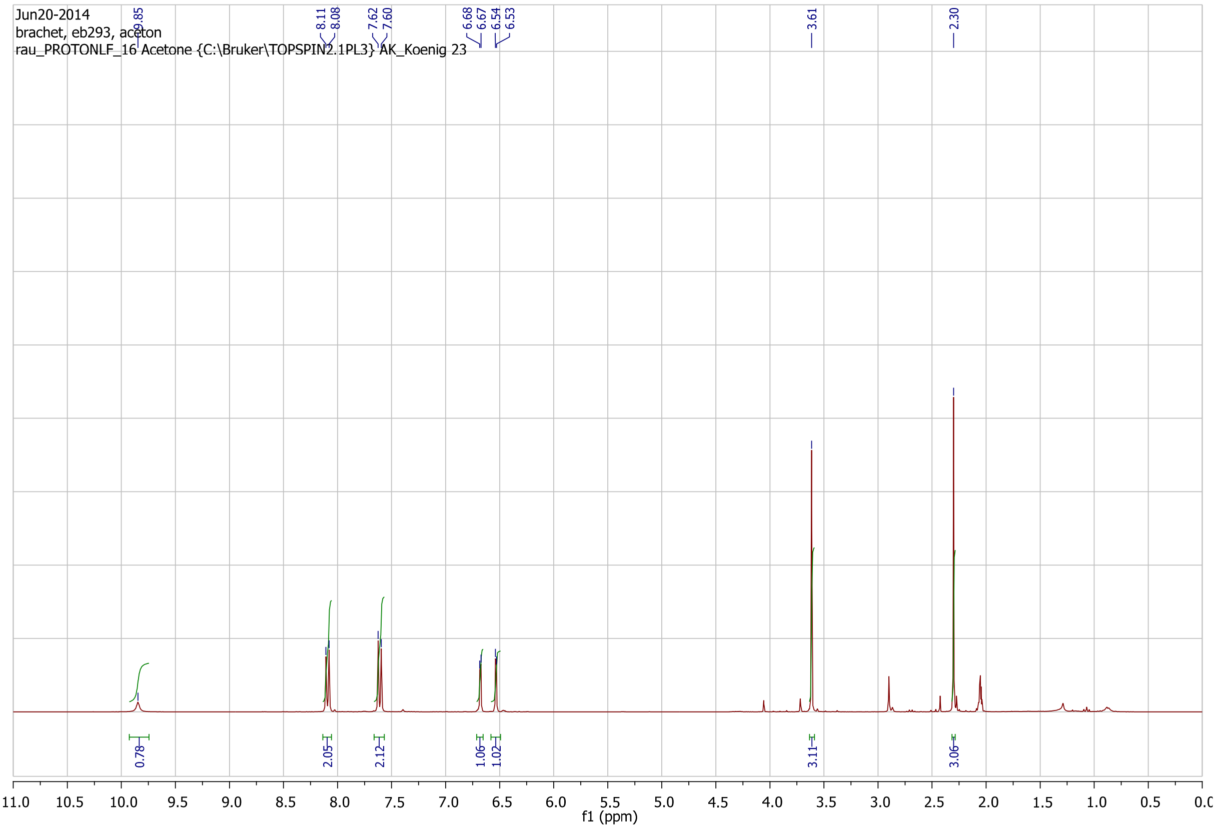

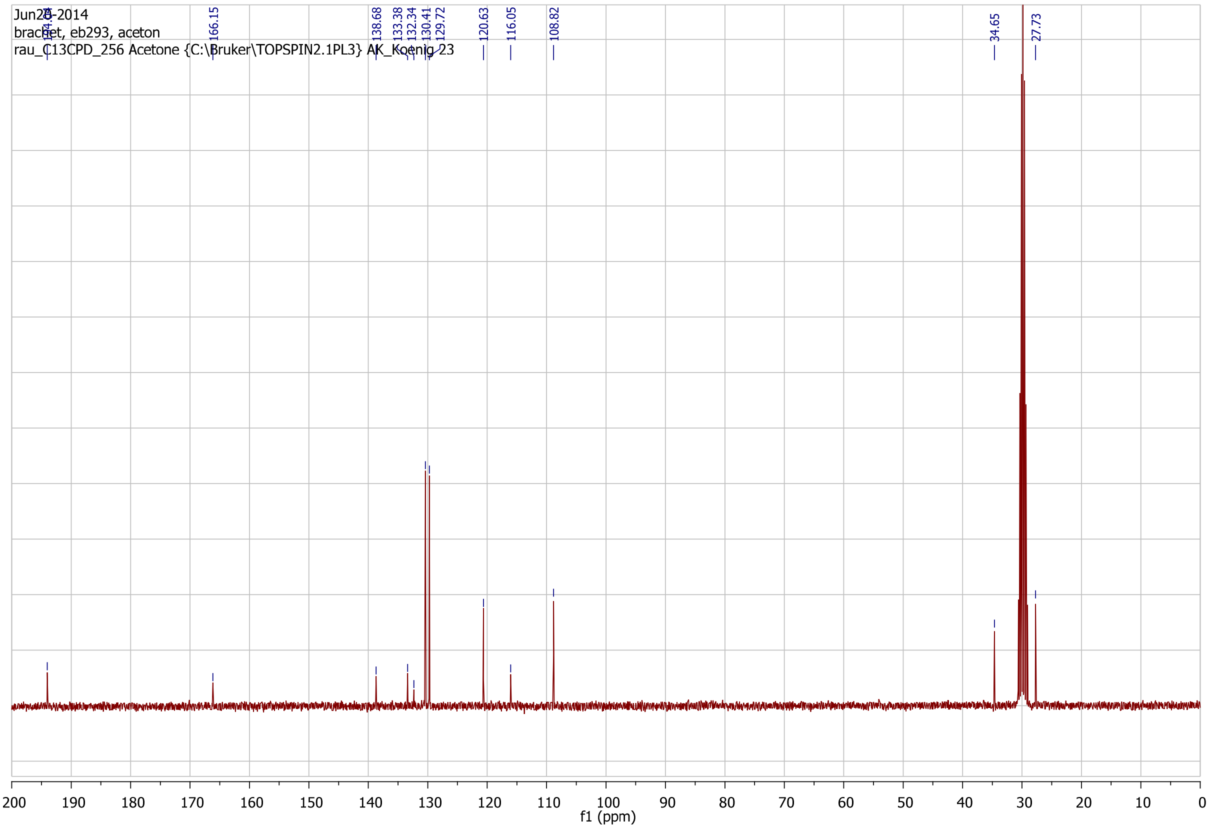

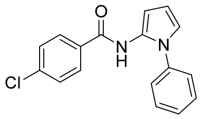

Compound 3r: 4-chloro-N-(1-phenyl-1H-pyrrol-2-yl)benzamide

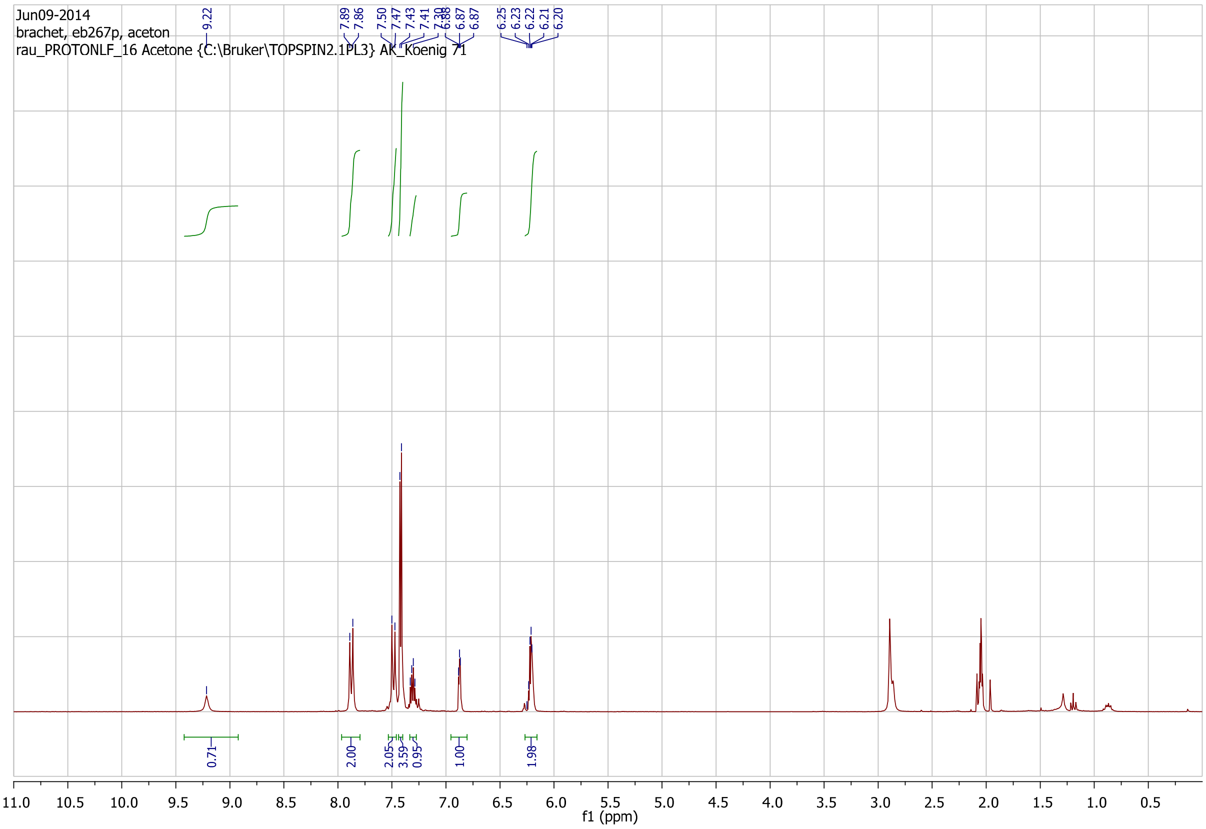

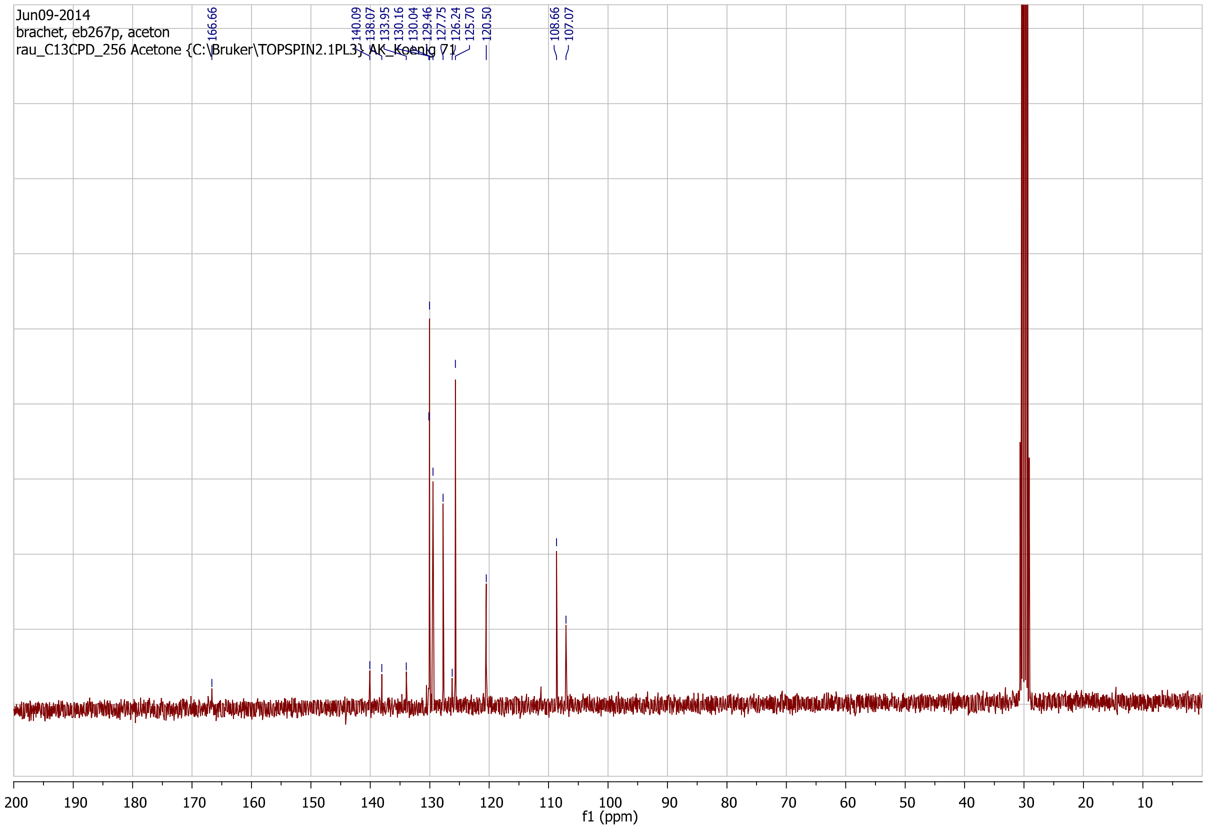

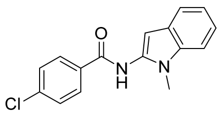

Compound 3s: 4-chloro-N-(1-methyl-1H-indol-2-yl)benzamide

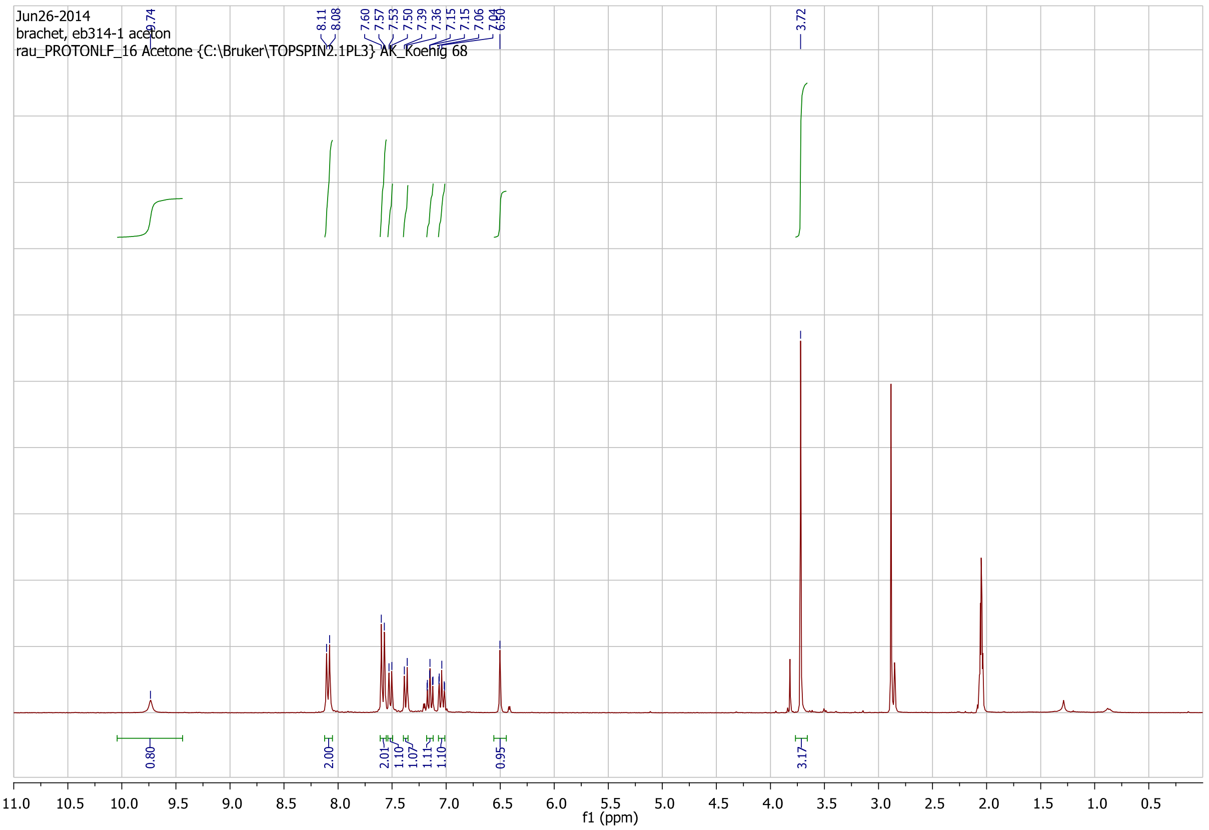

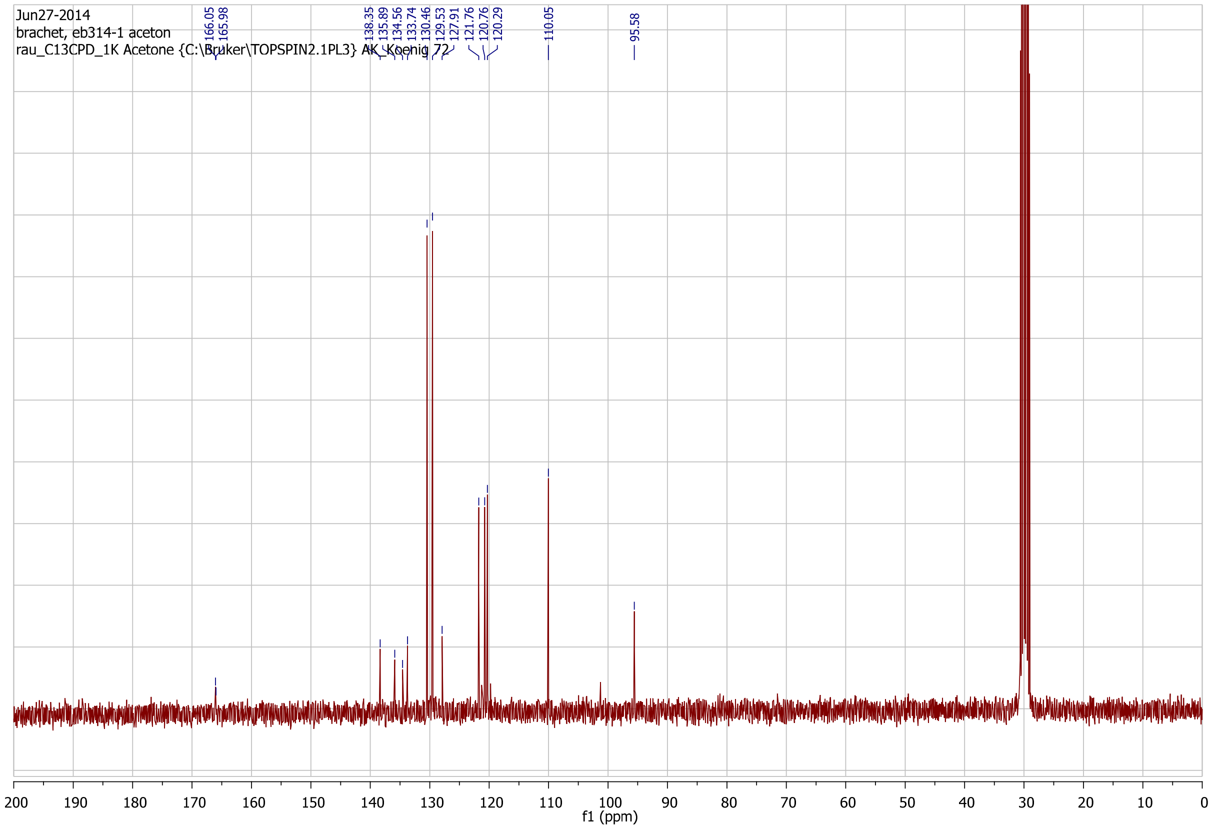

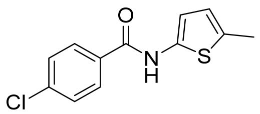

Compound 3t: 4-chloro-N-(5-methylthiophen-2-yl)benzamide

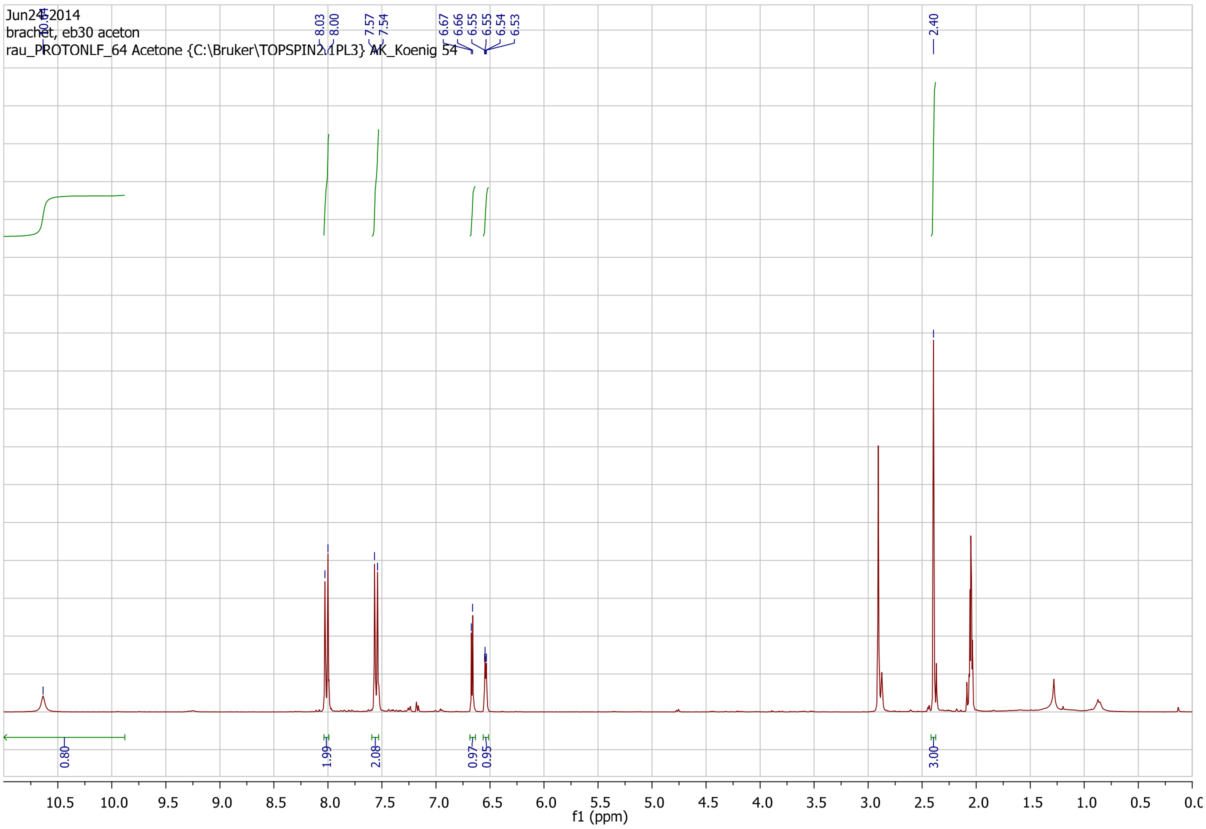

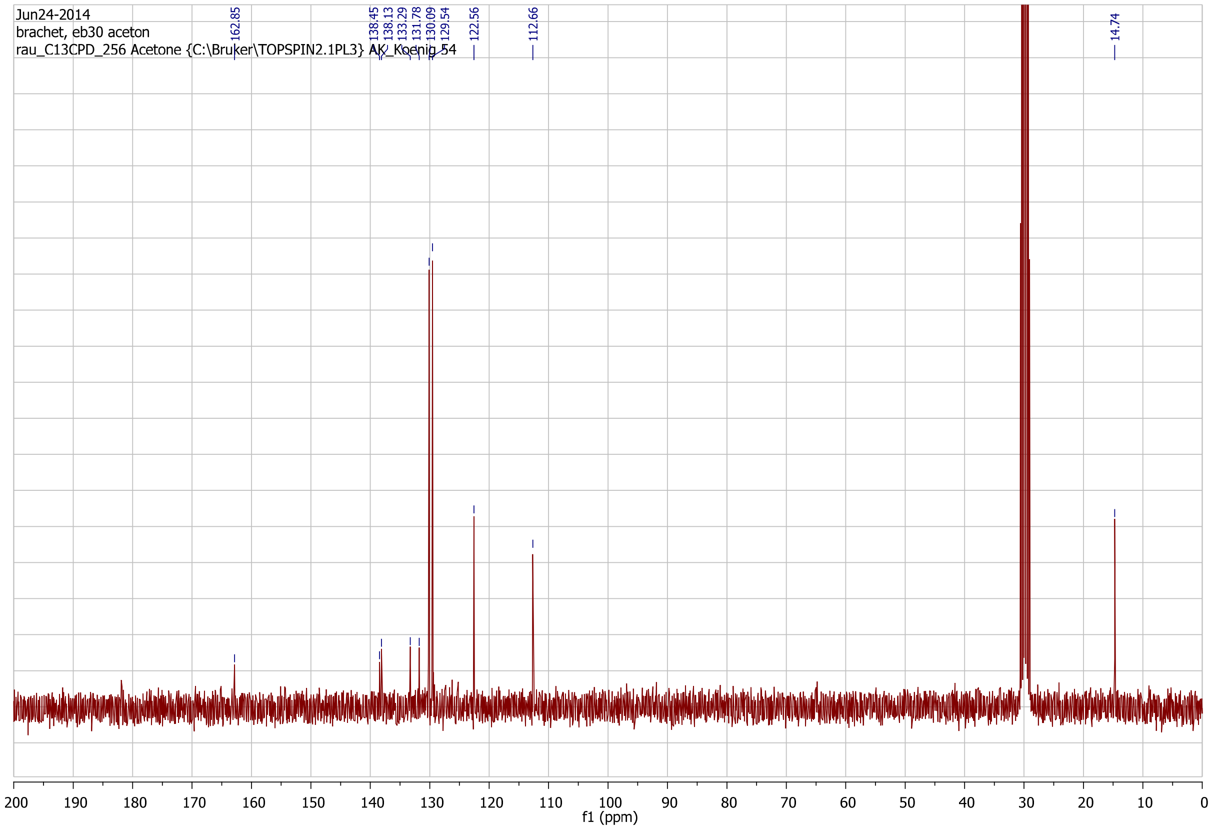

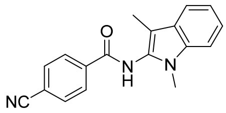

Compound 3u: 4-cyano-N-(1,3-dimethyl-1H-indol-2-yl)benzamide

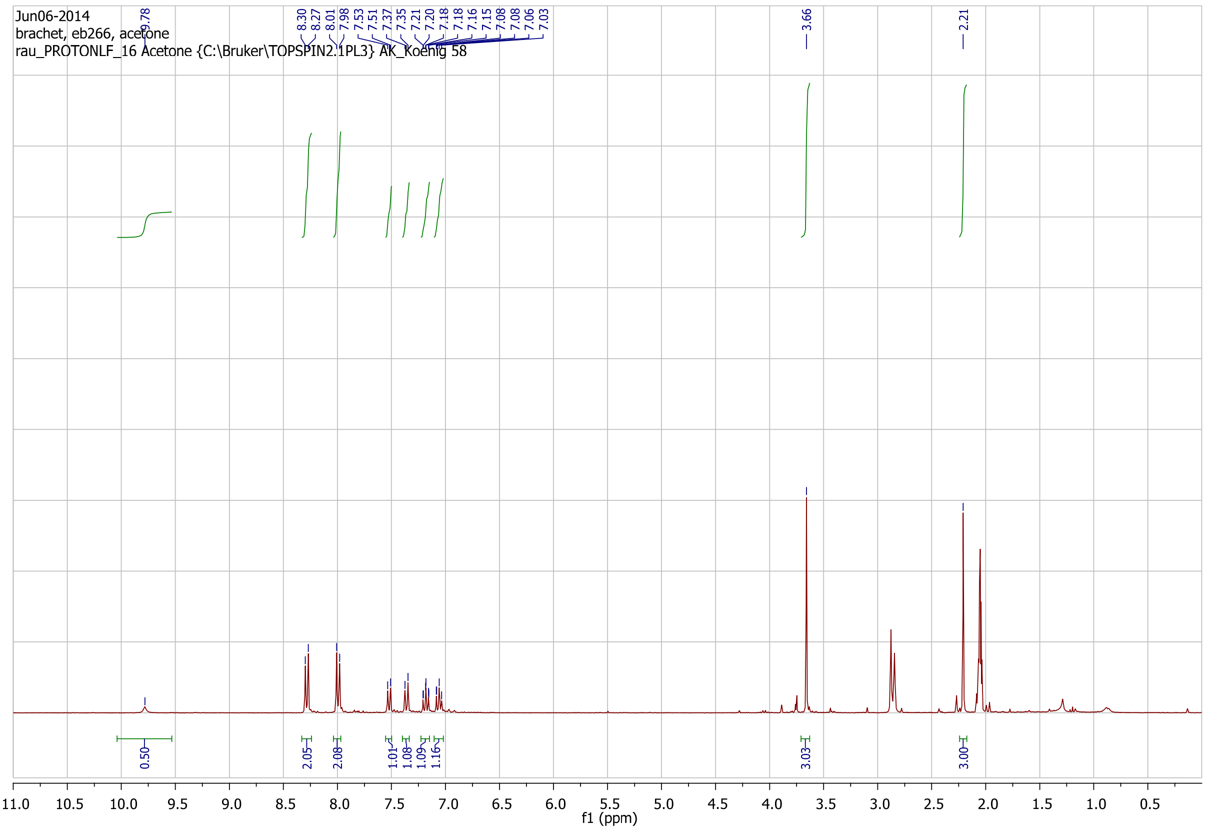

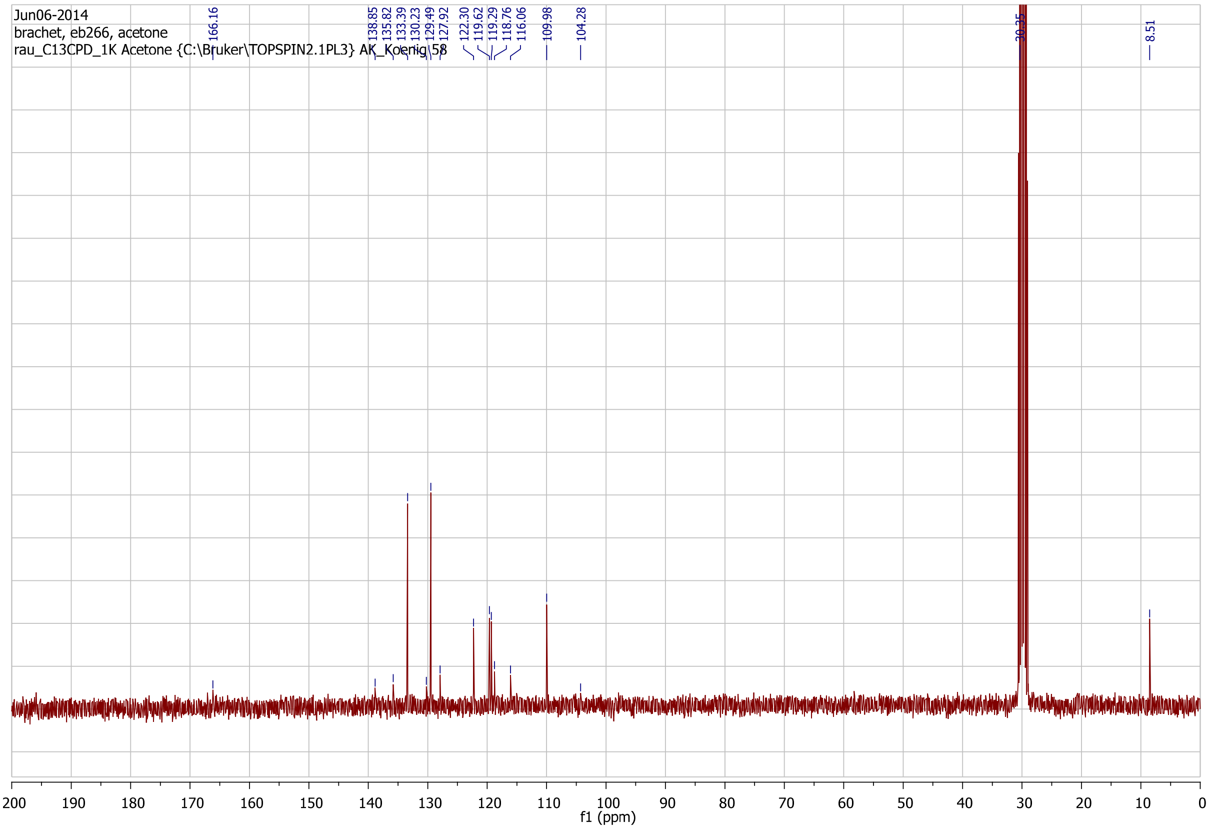

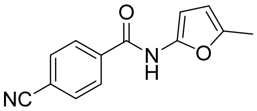

Compound 3v: 4-cyano-N-(5-methylfuran-2-yl)benzamide

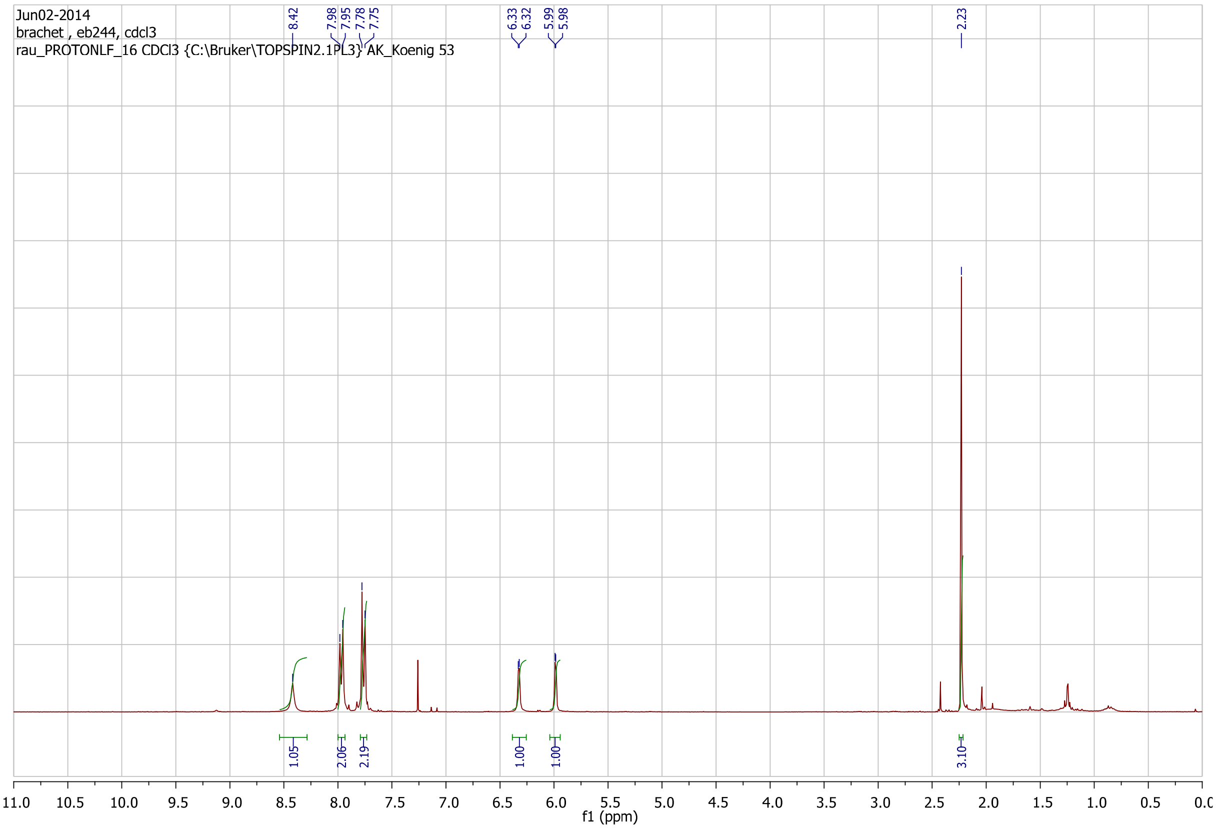

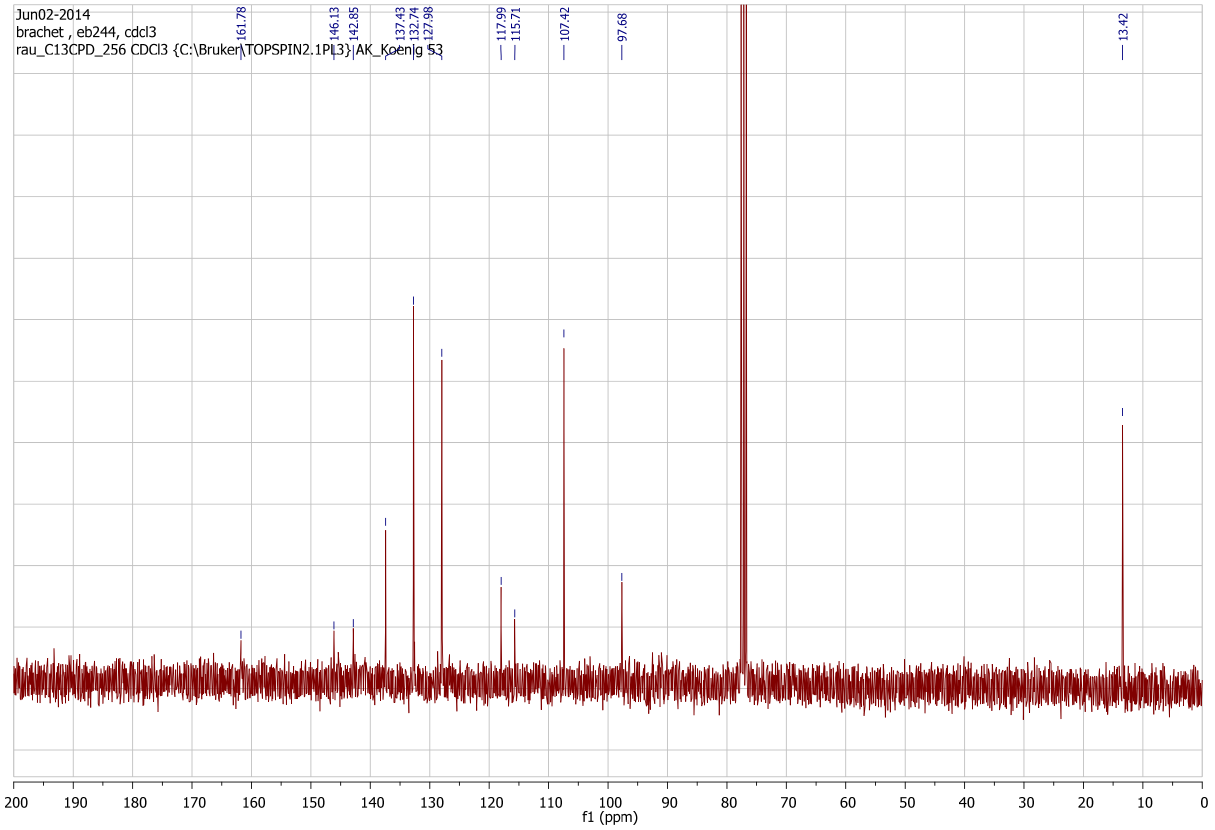

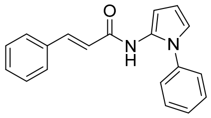

Compound 3w: N-(1-phenyl-1H-pyrrol-2-yl)cinnamamide

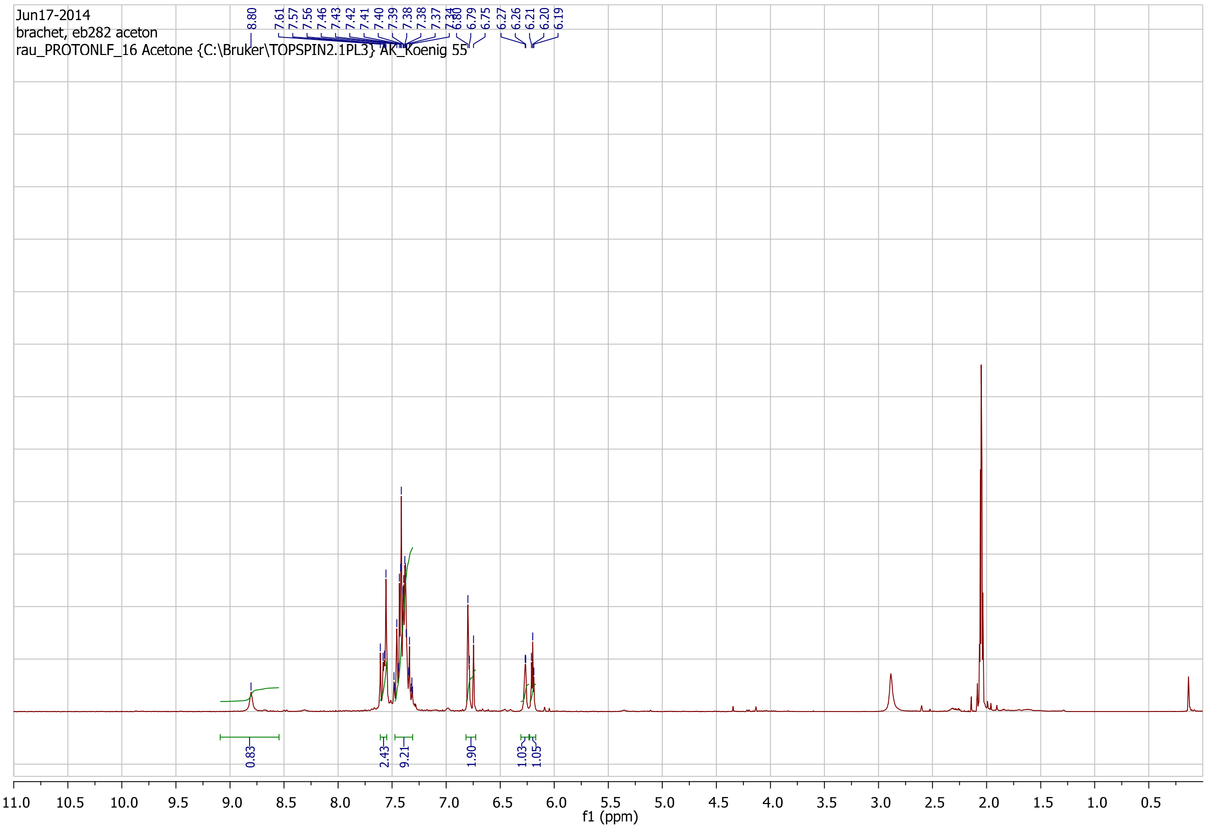

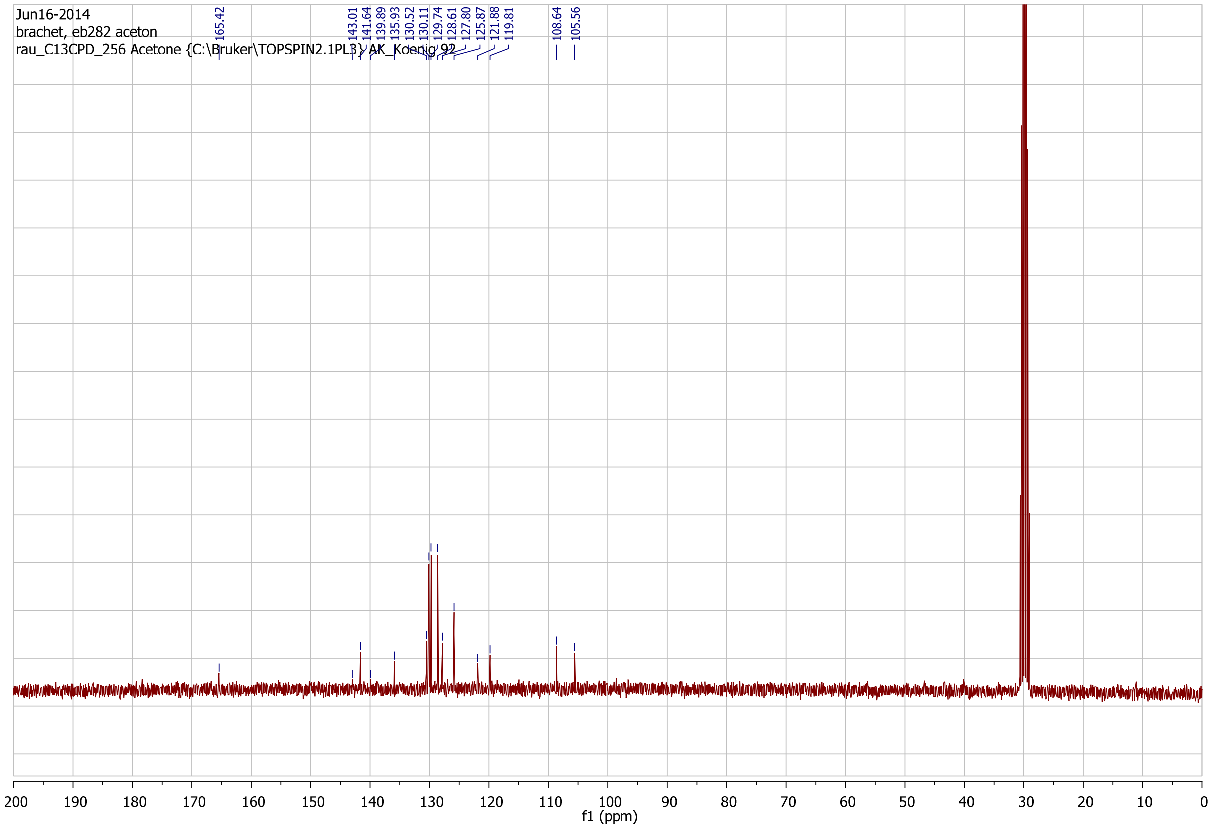

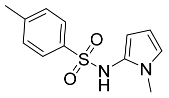

Compound 3x: 4-methyl-N-(1-methyl-1H-pyrrol-2-yl)benzenesulfonamide

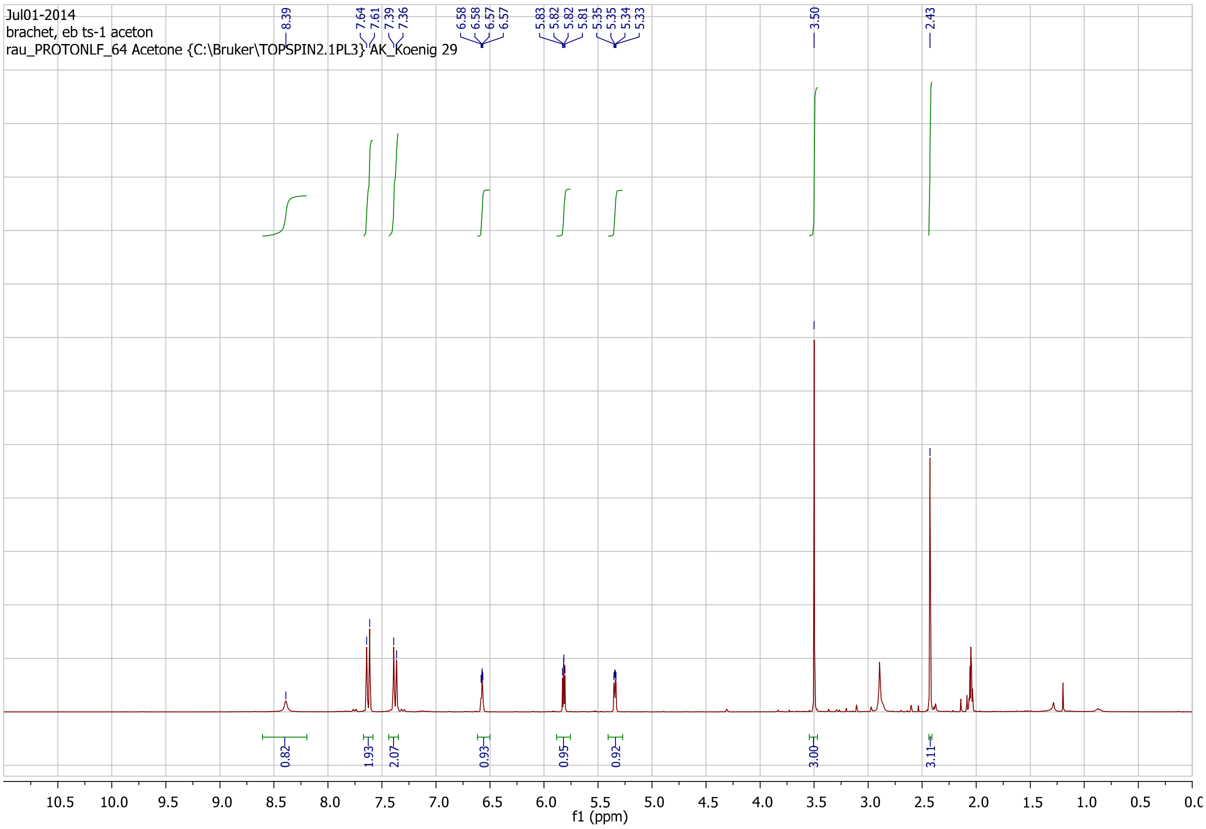

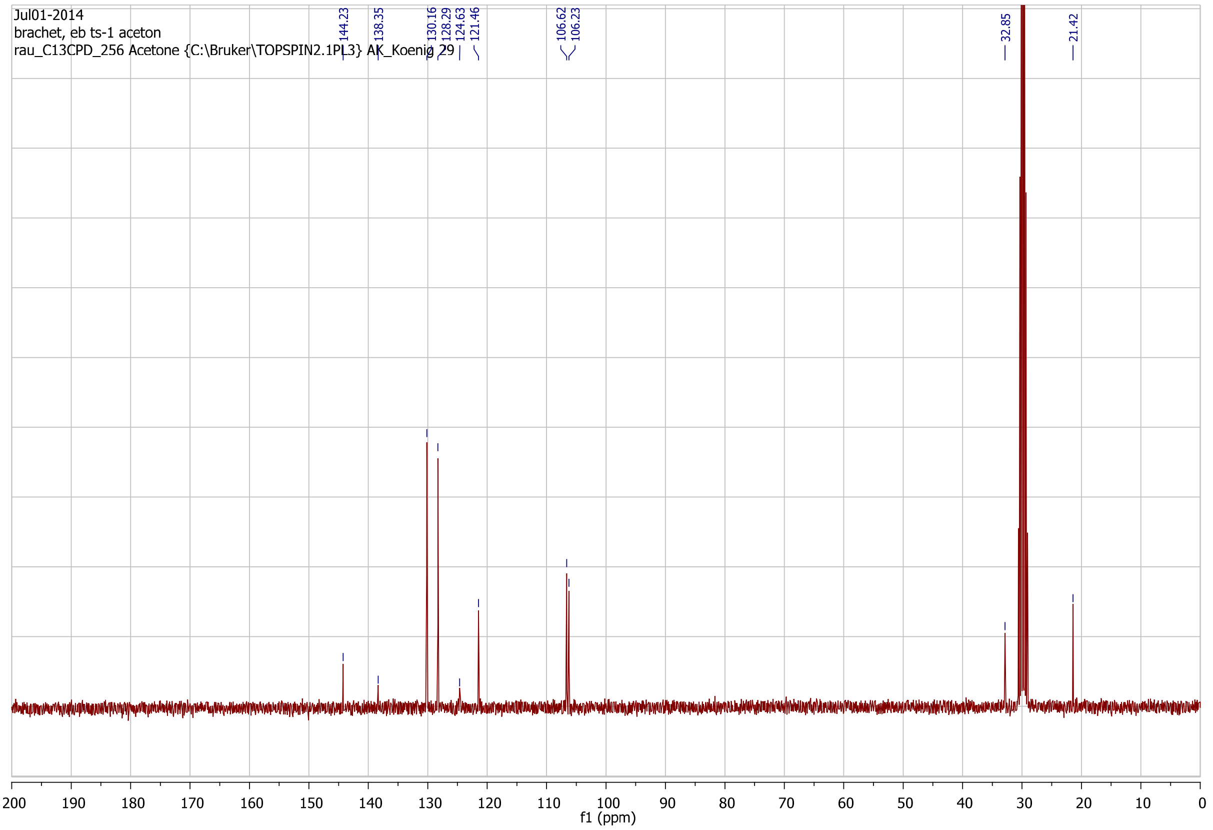

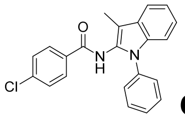

Compound 6: 4-chloro-N-(3-methyl-1-phenyl-1H-indol-2-yl)benzamide

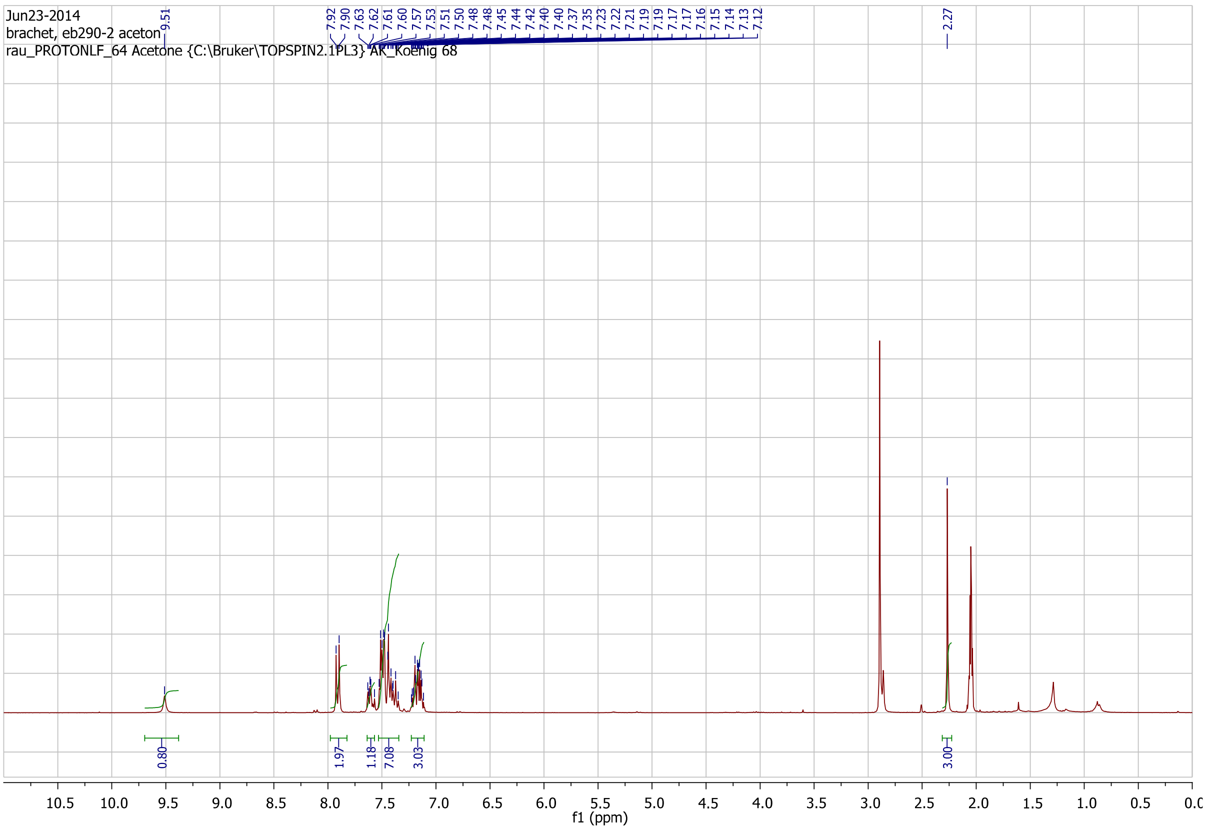

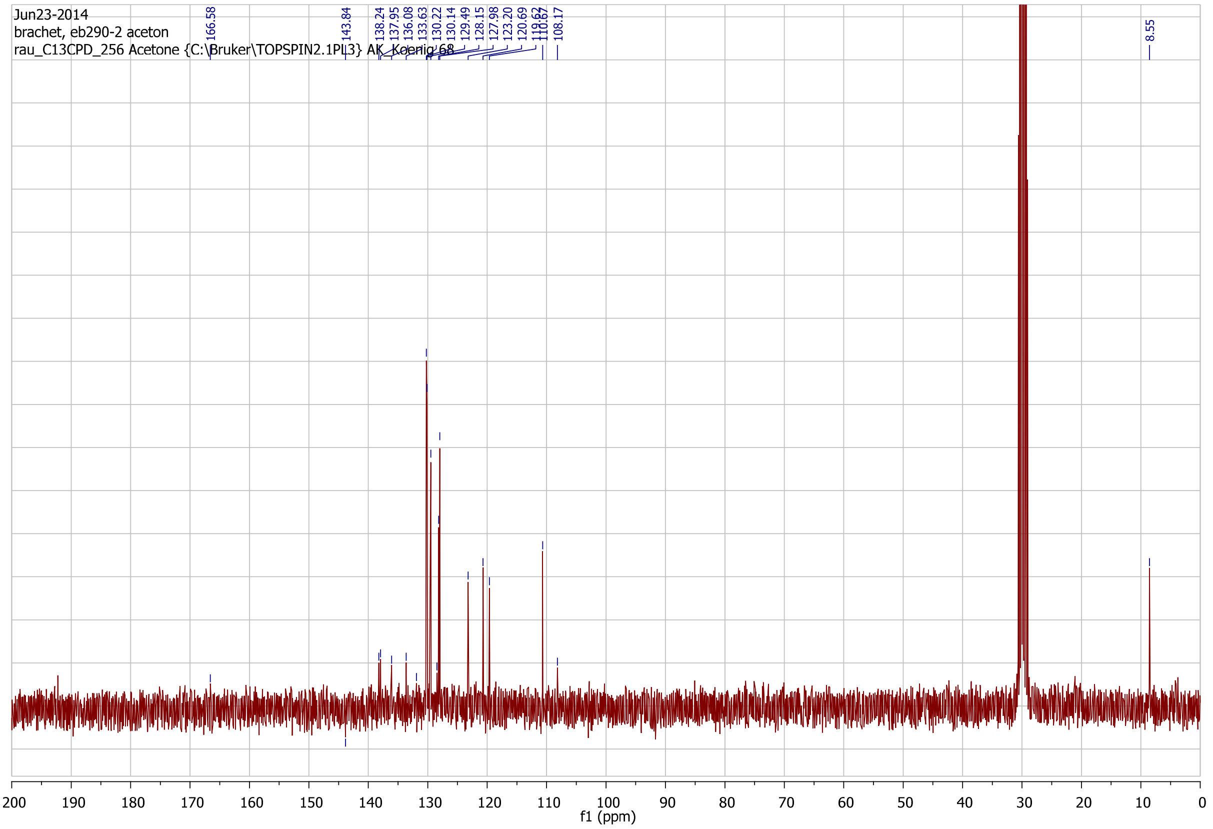

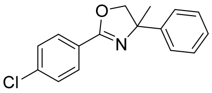

Compound 8: 2-(4-chlorophenyl)-4-methyl-4-phenyl-4,5-dihydrooxazole

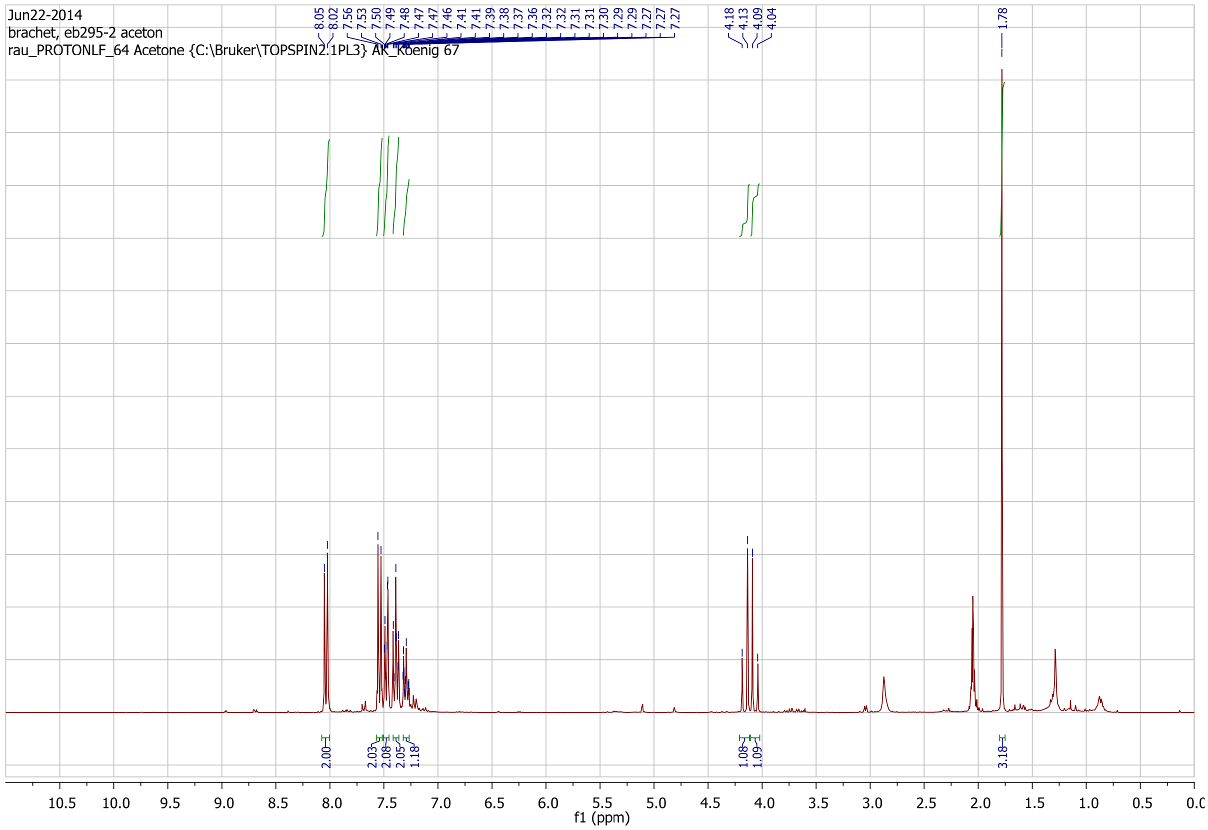

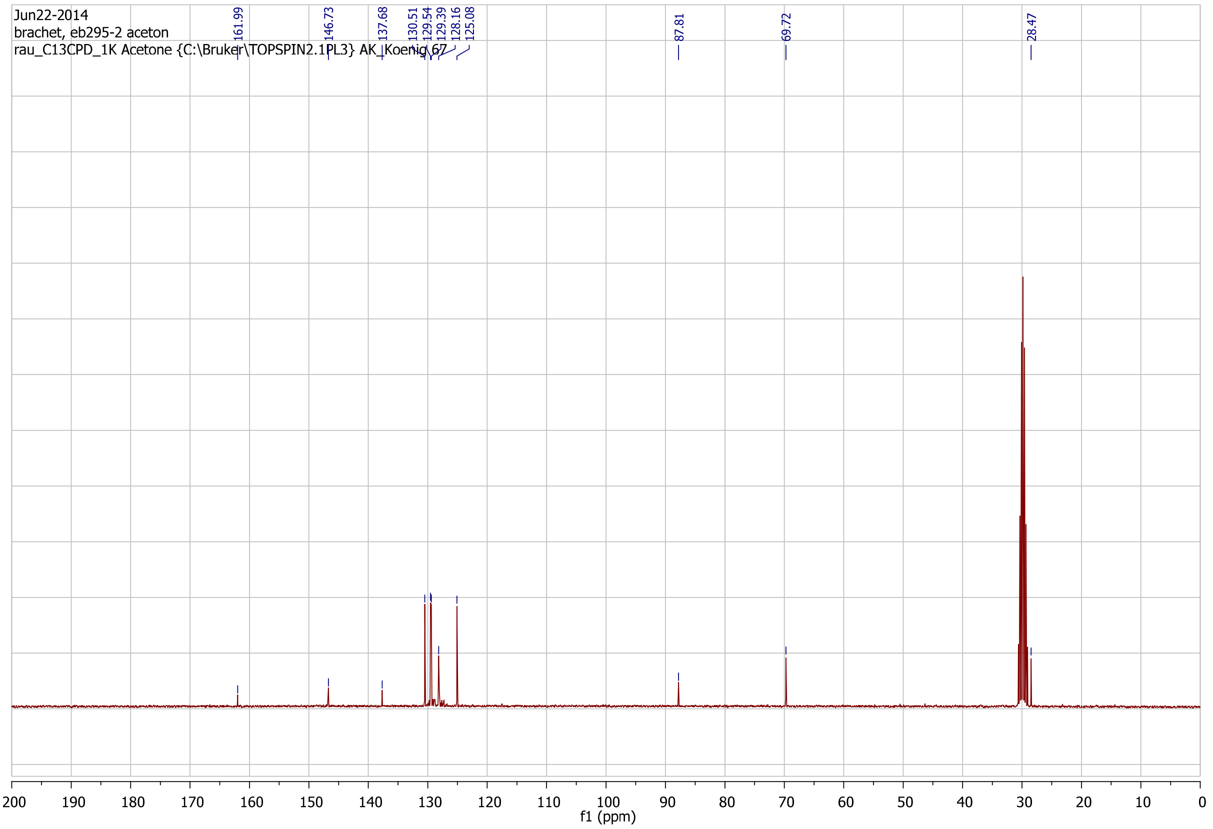

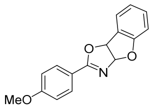

Compound 9: 2-(4-methoxyphenyl)-3a,8b-dihydrobenzofuro[2,3-d]oxazole

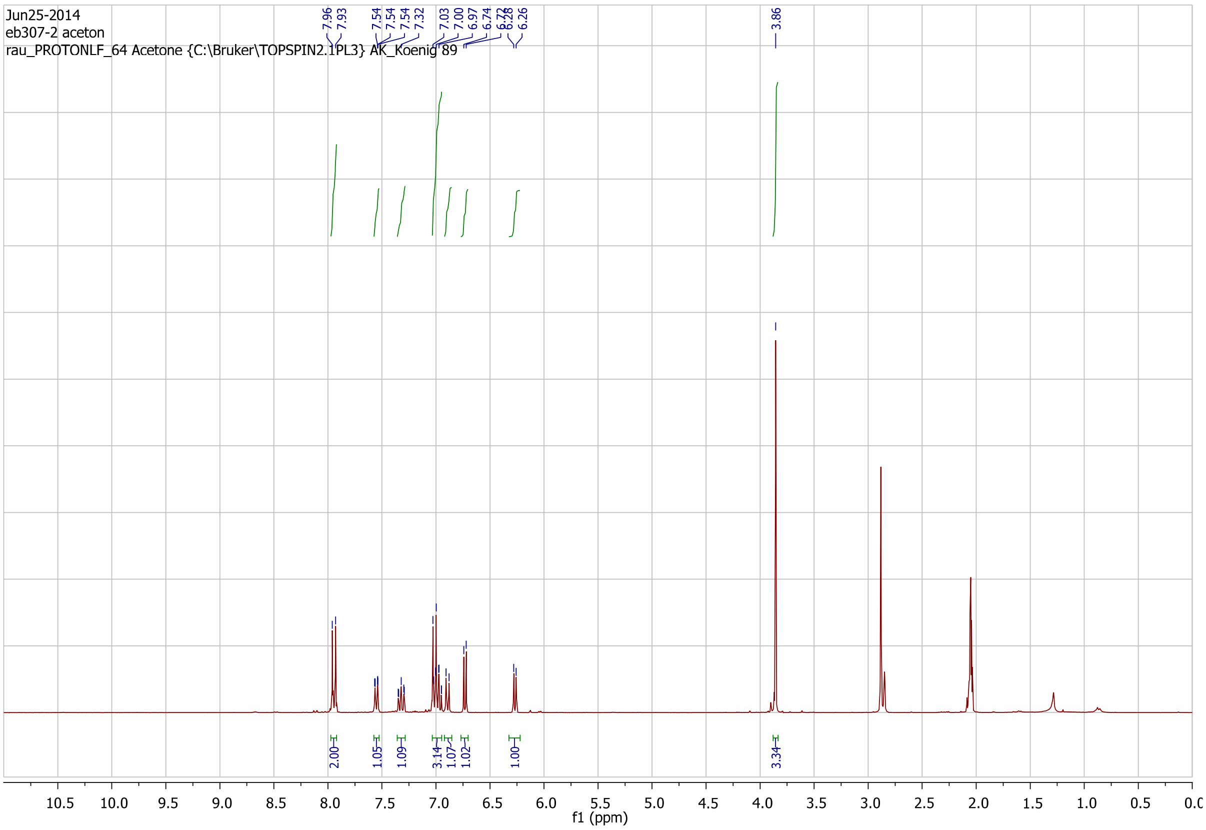

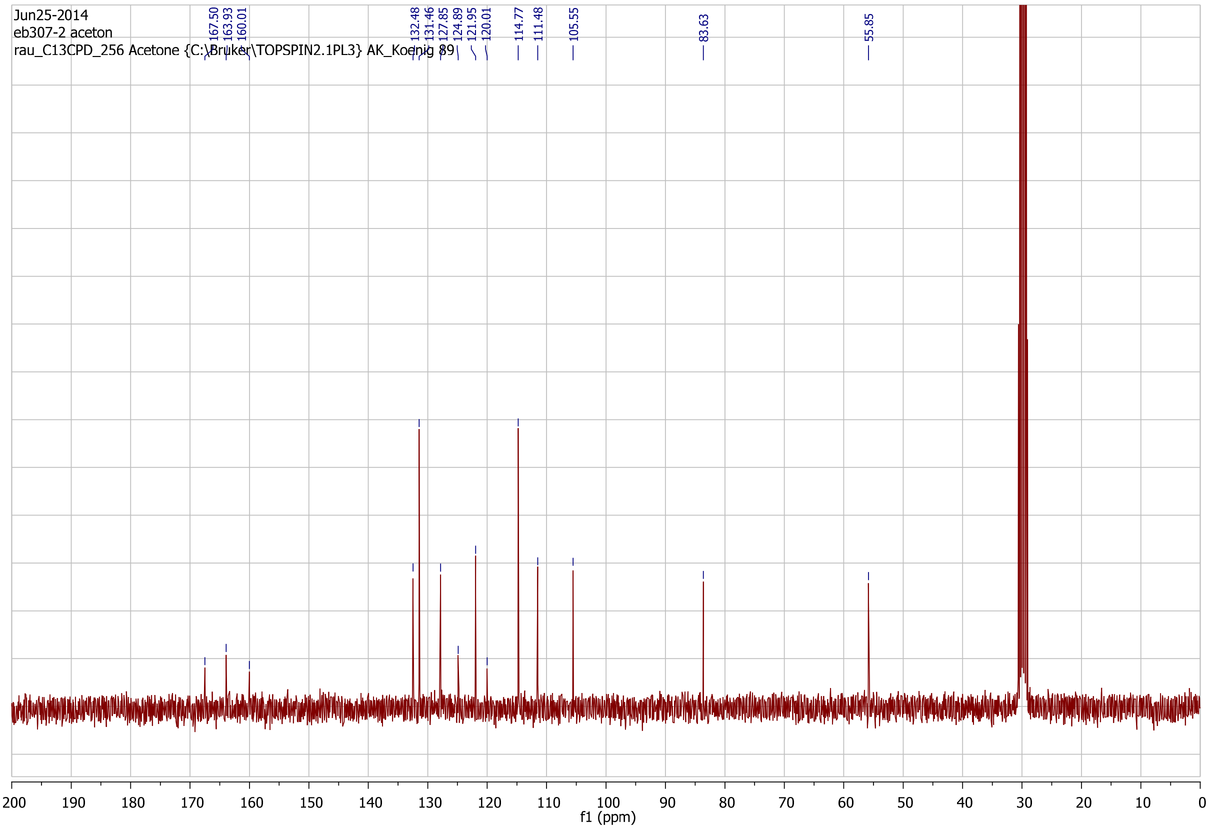

4) Crystallographic data

Compound 8

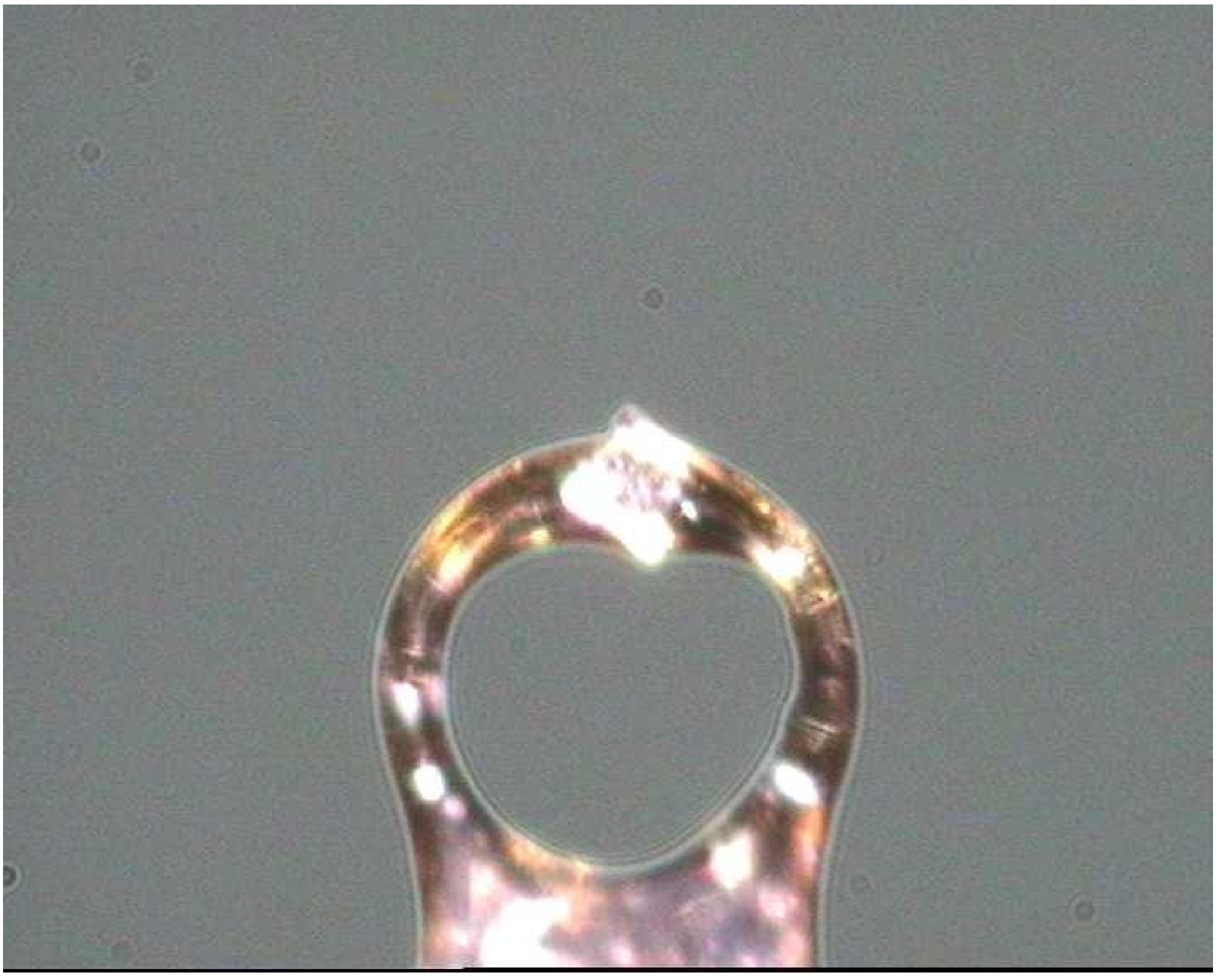

Bond precision: C-C = 0.0020 A Wavelength=1.54184
Cell: a=11.6434(3) b=10.03245(18) c=20.8570(4)
alpha=90 beta=103.161(2) gamma=90
Temperature: 123 K
Calculated Reported
Volume 2372.35(9) 2372.35(8)
Space group P 21/c P 1 21/c 1
Hall group -P 2ybc -P 2ybc
Moiety formula C13 H14 N2 O2 C13 H14 N2 O2
Sum formula C13 H14 N2 O2 C13 H14 N2 O2
Mr 230.26 230.26
Dx,g cm-3 1.289 1.289
Z 8 8
Mu (mm-1) 0.719 0.719
F000 976.0 976.0
F000’ 978.98
h,k,lmax 13,11,24 13,11,24
Nref 3888 3758
Tmin,Tmax 0.912,0.974 0.991,0.997
Tmin’ 0.908
Correction method= ANALYTICAL

Data completeness= 0.967 Theta(max)= 63.480
R(reflections)= 0.0366(3356) wR2(reflections)= 0.1027(3758)
S = 1.040 Npar= Npar = 367

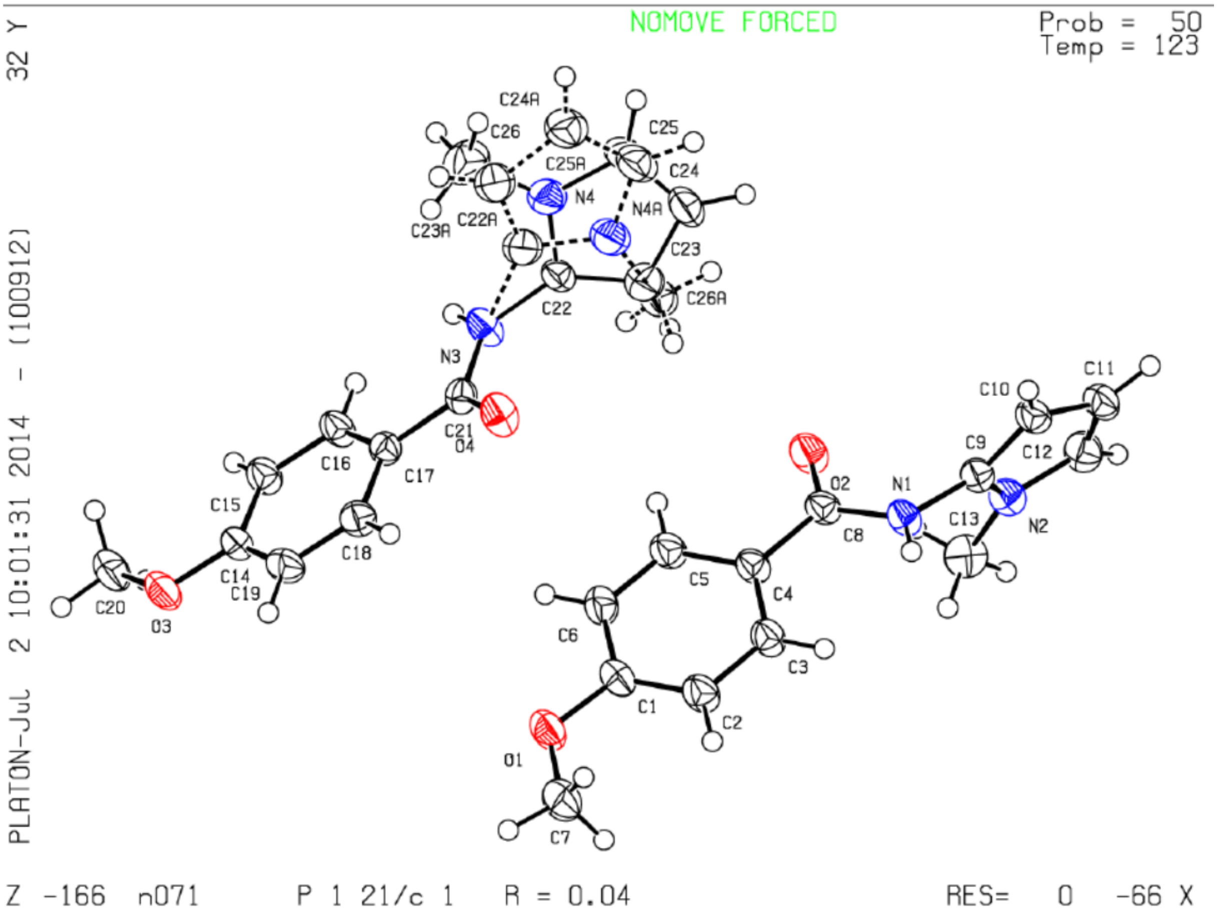

5) Cyclic voltammetry of compound 1b

Measurements were carried out with an **Autolab PGSTAT302N Metrohm**

Working electrode: Glassy Carbon

Counter electrode: Platinum wire

Pseudo reference electrode: Silver wire

Supporting electrolyte: Tetrabutylammonium tetrafluoroborate Fluka 0.1 M

Solvent : DMSO

DMSO was degassed with argon prior to the measurements. All experiments were performed under argon atmosphere. Ferrocene was used as an internal reference for the reduction and oxidation potentials.

Nested procedure	
Autolab control	
Set potential	0.000
Set cell	On
Wait time (s)	5
Optimize current range	5
CV staircase	[0.000, 1.600, -1.800, 0.000, 6, 0.0500000]
Start potential (V)	0.000
Upper vertex potential (V)	1.600
Lower vertex potential (V)	-1.800
Stop potential (V)	0.000
Number of stop crossings	6
Step potential (V)	-0.00488
Scan rate (V/s)	0.0500000
Estimated number of poin...	4192
Interval time (s)	0.097656

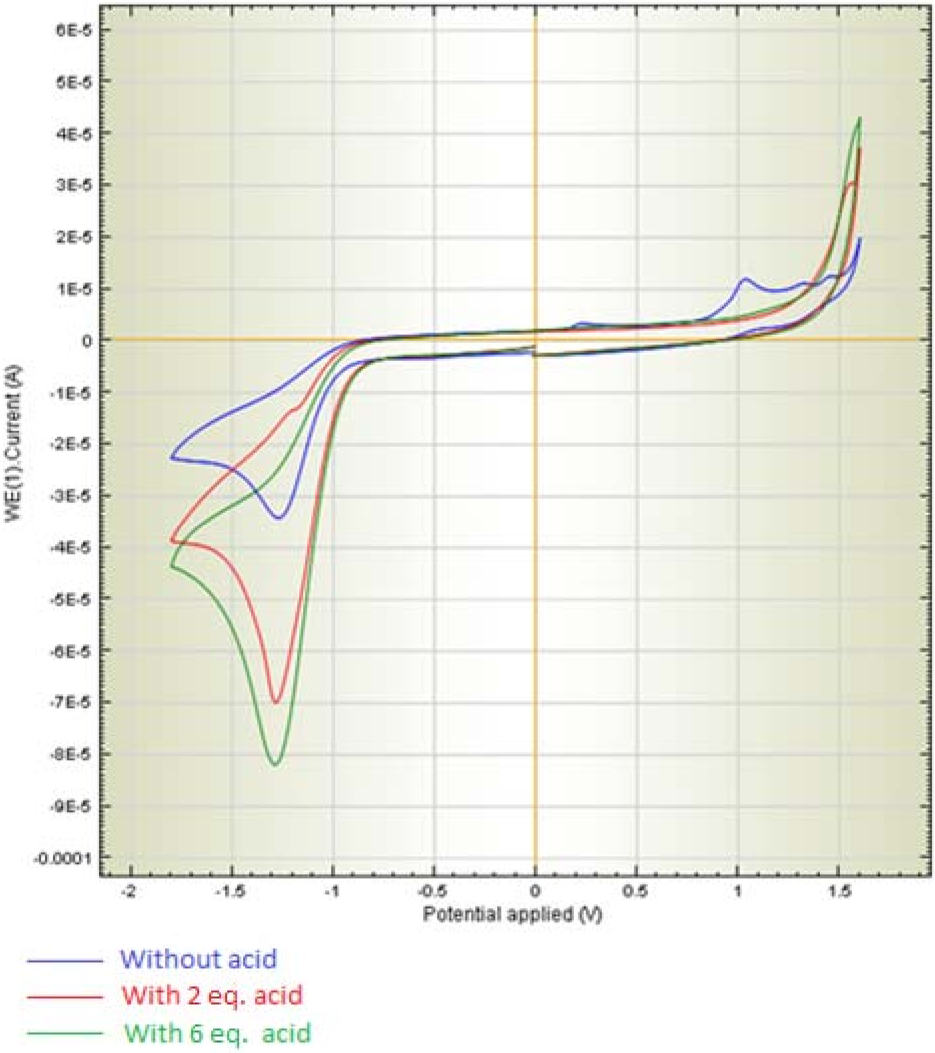

Reduction and oxidation potentials of compound **1b** (DMSO)

**Without Acid**	**With Acid**
$E_{Ox(DMSO)}$= + 0,37 V vs. Fe^{+}/Fe<br>$E_{Red(DMSO)}$= – 1,87 V vs. Fe^{+}/Fe	$E_{Ox(DMSO)}$= + 0,90 V vs. Fe^{+}/Fe<br>$E_{Red(DMSO)}$= – 1,87 V vs. Fe^{+}/Fe

6) Stern-Volmer analysis for $Ru(bpy)_3Cl_2$ x 6 H_2O + compound 1b

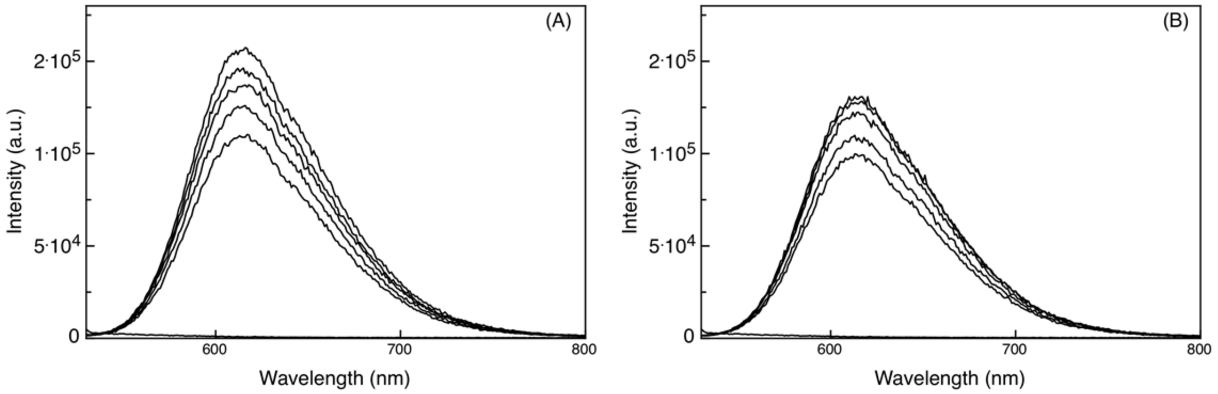

Left figure: Changes in the fluorescence spectra (in this case intensity) of $Ru(bpy)_3Cl_2$.6 H_2O upon successive addition of 4-chlorobenzoyl azide.
Right figure: Changes in the fluorescence spectra (in this case intensity) of $Ru(bpy)_3Cl_2$.6 H_2O upon successive addition of 4-chlorobenzoyl azide in the presence of H_3PO_4 (19.1 mg/mL).

Emission quenching of $Ru(bpy)_3Cl_2.6H_2O$ in the presence of increasing amounts of compound **1g** in DMSO at 25 ^{0}C. The concentration of $Ru(bpy)_3Cl_2.6H_2O$ was 0.o5 mM and the concentration of **1g** was increased from 0 to 0.25 mM. Excitation wavelength was 455 nm.

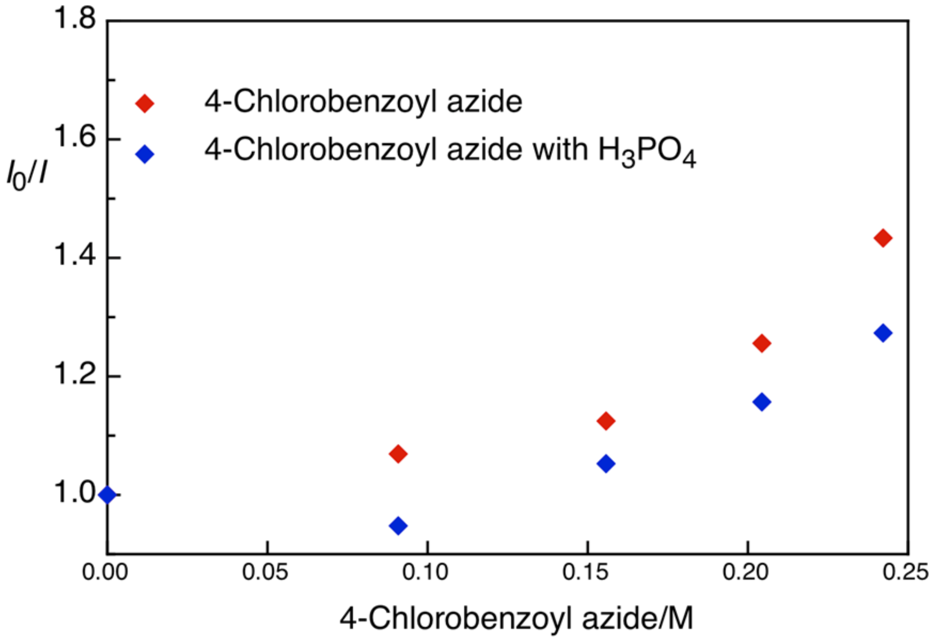

Stern-Volmer plot for $Ru(bpy)_3Cl_2.6H_2O$ + compound **1g** in DMSO at 25 ^{0}C. The concentration of $Ru(bpy)_3Cl_2$. 6 H_2O was 0.05 mM. Excitation wavelength was 455 nm. 19.1 mg/mL H_3PO_4.

Fluorescence emission spectra were recorded on a Cary Eclipse Fluorescence spectrophotometer. Solutions containing $Ru(bpy)_3Cl_2.6H_2O$ with varying concentrations of the quencher were excited at

455 nm and the emission intensities at ca. 620 nm were determined. DMSO was degassed before measurements. I_0 and I represent the intensities of the emission in the absence and presence of the quencher at ca. 620 nm.

The triplet energy of acyl azides is reported to be ~ 41 kcal/mol.[10] The triplet energy of phenyl azide is estimated to be 68 kcal/mol.[11]

7) Time profile of the reaction of 1b with 2a with and without blue light irradiation.

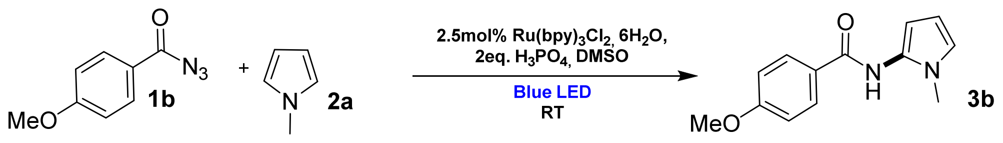

We performed an experiment with "on-off" switching of the light source in the reaction of compound **1b** and **2a** under standard condition with mesitylene as internal standard. The internal standard was added to the reaction mixture with the substrates. We start with 30 min of irradiation followed by 30 min stirring in the dark. Next, 1 hour of irradiation followed by 1 hour of stirring in the dark. One additional hour of irradiation is needed to complete the reaction in this case. Without light the reaction conversion stops immediately. The conversion of **1b** was determined by GC using mesitylene as the internal standard. The results indicate that the reaction is not a radical chain process and the conversion requires the light excitation.

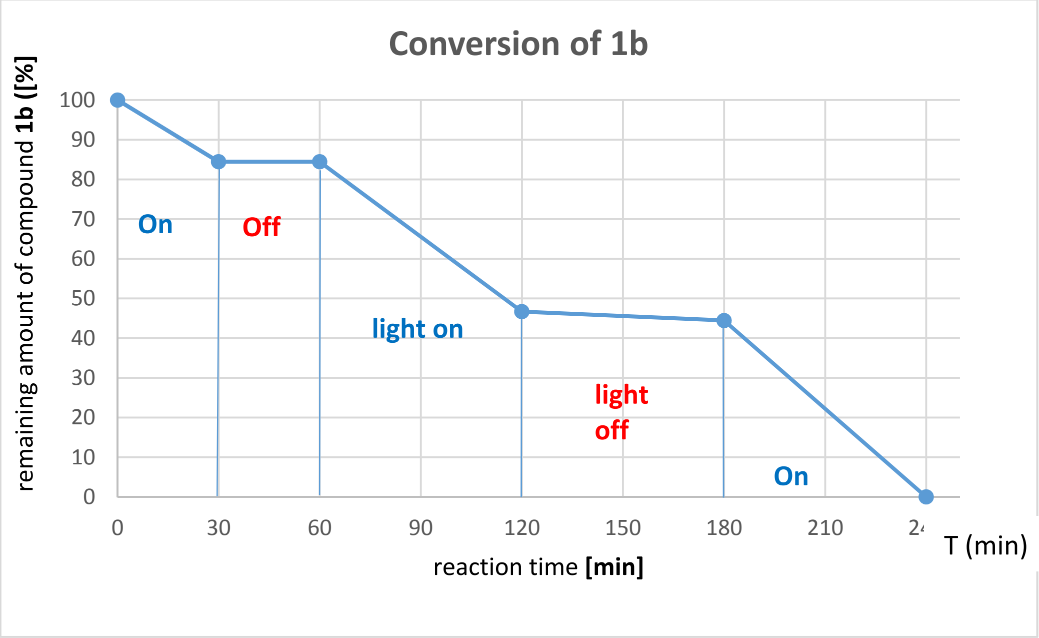

8) GC-MS analysis of crude reaction mixtures

```
File        :M:\GCMS\Ghosh(T)\G0866_00.D
Operator    : R. Vasold
Acquired    : 19 Sep 2014   10:25      using AcqMethod G0866_00.M
Instrument :    MSD 5975 PAL2
Sample Name: TG-EB-C6
Misc Info   :
Vial Number: 1
```

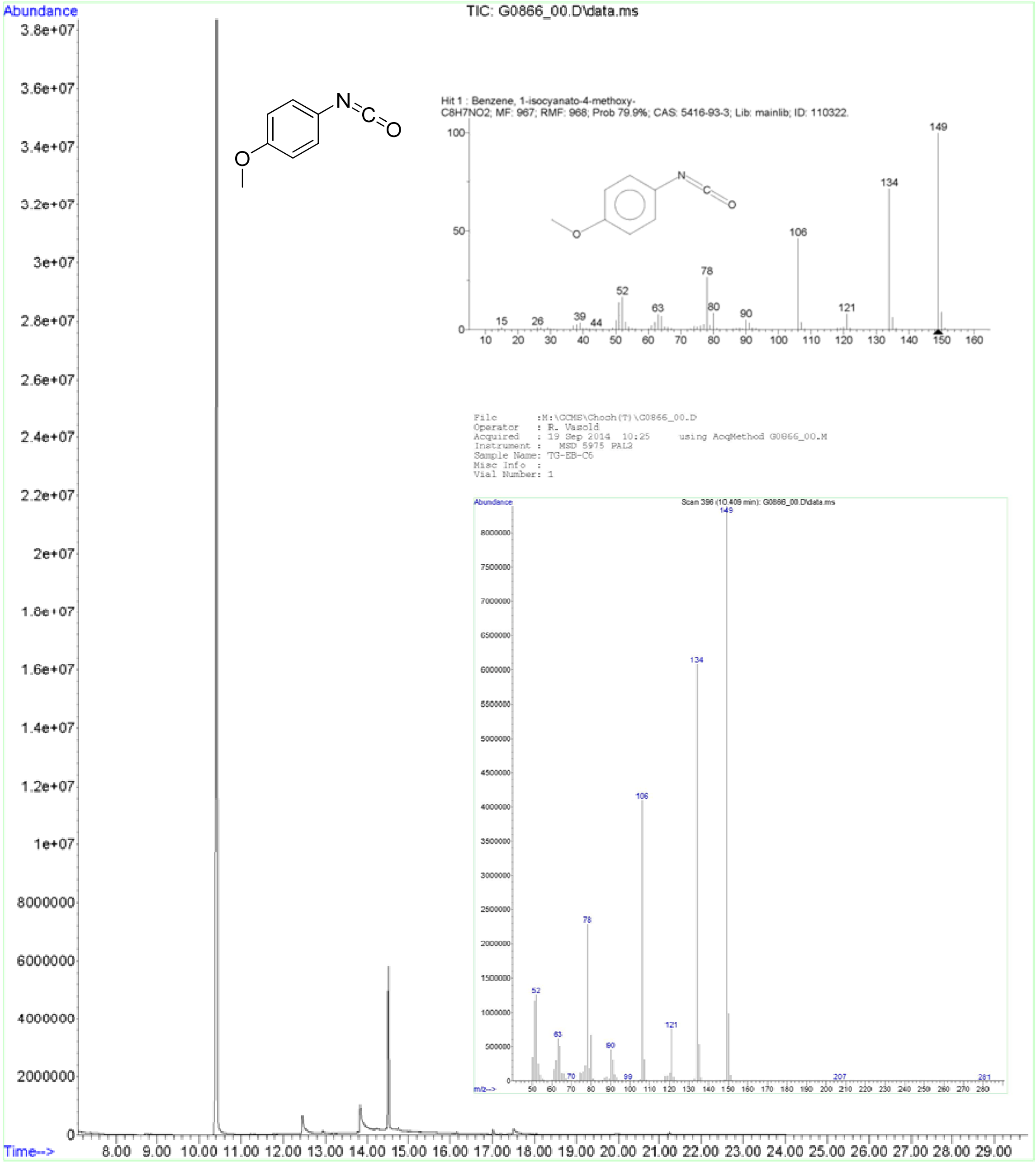

Reaction conditions: Compound **1b**, *N*-methylpyrrole, $Ru(bpy)_3Cl_2 \cdot 6\ H_2O$ in DMSO under N_2. Blue LED (λ_{Ex} = 455 nm) irradiation for 1.5 hours.

```
File        :M:\GCMS\Ghosh(T)\G0867_04.D
Operator    : R. Vasold
Acquired    : 22 Sep 2014  15:16     using AcqMethod G0867_00.M
Instrument  :   MSD 5975 PAL2
Sample Name: TG-EB-C11
Misc Info  :
Vial Number: 5
```

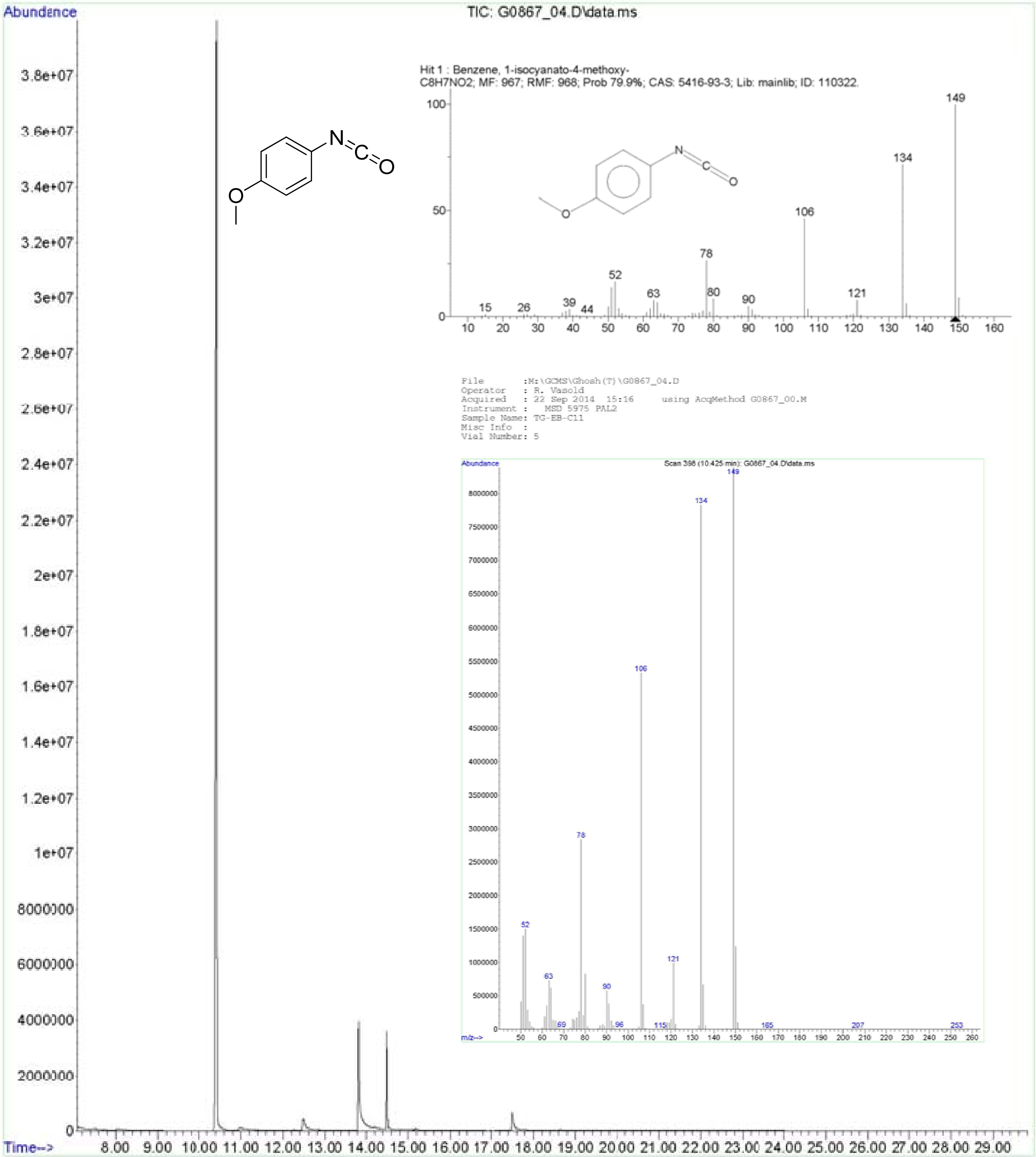

Reaction conditions: Compound **1b** and $Ru(bpy)_3Cl_2$. 6 H_2O were irradiated in DMSO under N_2 with a blue LED (λ_{Ex} = 455 nm) for 1.5 hours.

```
File        :M:\GCMS\Ghosh(T)\G0866_01.D
Operator    : R. Vasold
Acquired    : 19 Sep 2014   11:35      using AcqMethod G0866_00.M
Instrument  :    MSD 5975 PAL2
Sample Name: TG-EB-C7
Misc Info   :
Vial Number: 1
```

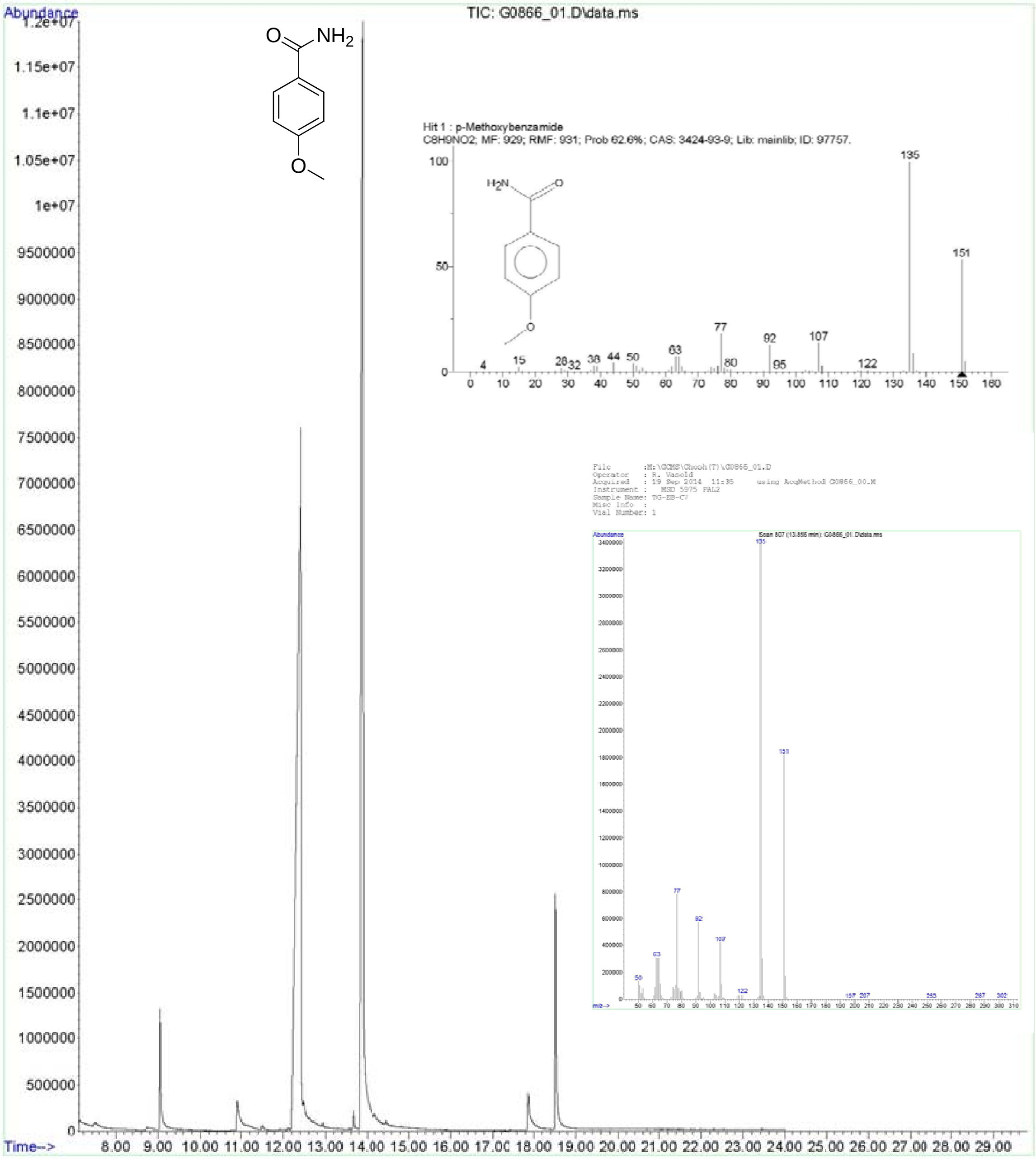

Reaction conditions: Compound **1b**, N-methylpyrrole, $Ru(bpy)_3Cl_2 \cdot 6\,H_2O$ and H_3PO_4 in DMSO under N_2. Irradiation by blue LED (λ_{Ex} = 455 nm) for 1.5 hours.

```
File        :M:\GCMS\Ghosh(T)\G0866_01.D
Operator    : R. Vasold
Acquired    : 19 Sep 2014   11:35      using AcqMethod G0866_00.M
Instrument :   MSD 5975 PAL2
Sample Name: TG-EB-C7
Misc Info   :
Vial Number: 1
```

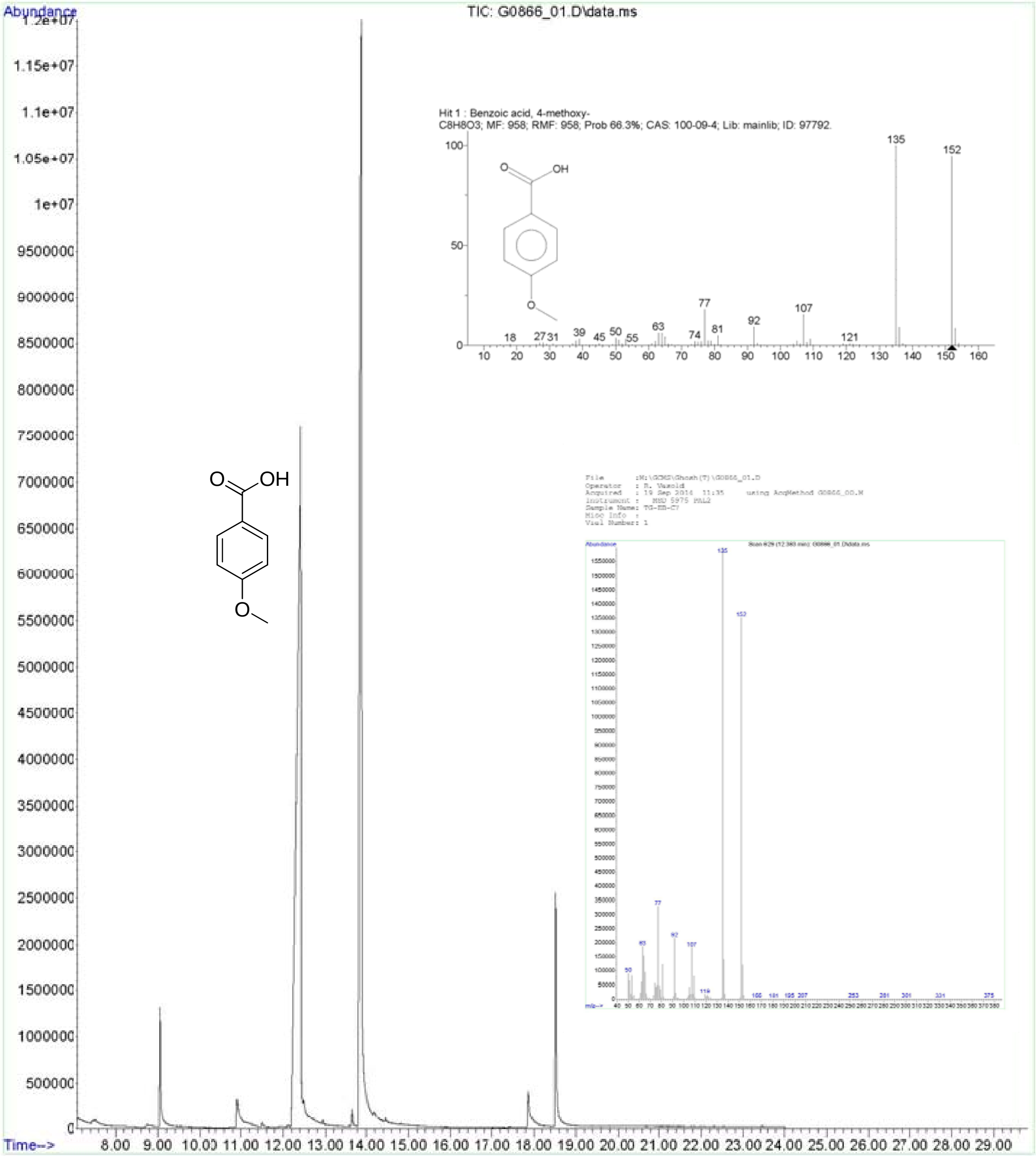

Reaction conditions: Compound **1b**, *N*-methylpyrrole, $Ru(bpy)_3Cl_2$. 6 H_2O and H_3PO_4 in DMSO under N_2. Irradiation by blue LED (λ_{Ex} = 455 nm) for 1.5 hours.

9) References

[1] A. R. Katritzky, K. Widyan, K. Kirichenko *J. Org. Chem.* **2007**, *72*, 5802.

[2] S. De Sarkar, A. Studer *Org. Lett.*, **2010**, *12*, 1992.

[3] Z. Zhong, J. Hu, X. Wang, J. Liu, L. Zhang *Synth. Commun.* **2011**, *41*, 2461.

[4] N. D. Arote, K. G. Akamanchi *Tetrahedron Lett.* **2007**, *48*, 5661.

[5] T. Shingaki, Nippon Kagaku Zasshi **1959**, *80*, 55.

[6] Z. Zhong, J. Hu, X. Wang, J. Liu, L. Zhang *Synth. Commun.* **2011**, *41*, 2461.

[7] N. D. Arote, K. G. Akamanchi *Tetrahedron Lett.* **2007**, *48*, 5661.

[8] D. Blanger, X. Tong, S. Soumar, Y. L. Dory, Y. Zhao *Chem. Eur. J.* **2009**, *15*, 4428.

[9] A. Carrër, J.-C. Florent, E. Auvrouin , P. Rousselle , E. Bertounesque *J. Org. Chem.*, **2011**, *76*, 2502.

[10] M. Sukigara, K. Honda, S. Kikuchi, *J. Soc. Photographic Sci. Techn. Japan,* **1971**, *34*, 209.

[11] L. J. Leyshon, A. Reiser *J. Chem. Soc., Faraday Trans. 2*, **1972**, *68*, 1918.
